# Synthesis of tetracyclic spiroindolines by an interrupted Bischler–Napieralski reaction: total synthesis of akuammicine†

**DOI:** 10.1039/d1ob01966j

**Published:** 2021-10-27

**Authors:** Matteo Faltracco, Eelco Ruijter

**Affiliations:** Department of Chemistry & Pharmaceutical Sciences, Amsterdam Institute of Molecular & Life Sciences (AIMMS), Vrije Universiteit Amsterdam De Boelelaan 1108 1081 HZ Amsterdam The Netherlands e.ruijter@vu.nl

## Abstract

Judicious substrate design allows interruption of the classical Bischler–Napieralski reaction, providing access to a range of diversely substituted tetracyclic spiroindolines. These complex polycyclic scaffolds are valuable building blocks for the construction of indole alkaloids, as showcased in a concise total synthesis of (±)-akuammicine.

## Abstract 2

The interrupted Bischler–Napieralski reaction of β,γ-unsaturated tryptamides affords tetracyclic spiro pyrroloindolines, which can be used in the total synthesis of the Strychnos alkaloid, akuammicine.

## Graphical abstract

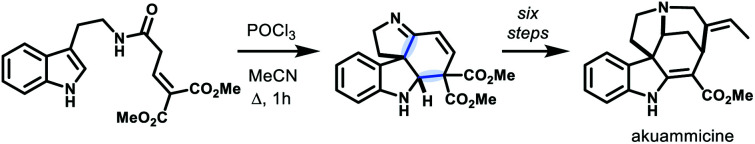

Cascade reactions allow the rapid generation of molecular complexity through multiple sequential bond-forming (and bond-breaking) events in a one-pot process. These exceptional advantages over traditional stepwise procedures make them particularly suited to efficiently access complex molecules such as natural products.^1^ Consequently, the use of cascade reactions in total synthesis is a well-established and prolific field that is constantly expanding in both methodology and scope, covering polyketides,^2^ alkaloids,^3^ terpenes,^4^ and steroids.^5^ Owing to their abundance, structural variety, and diverse biological activities, indole alkaloids occupy a prominent position among natural products (Fig. 1).^6^ As a result, they have attracted considerable interest from the synthetic community, leading to numerous novel approaches to this compound class in recent years.^7^

**Fig. 1:**
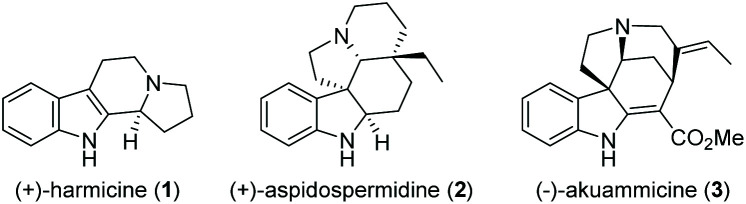
Representative indole alkaloids.

Synthetic approaches toward β-carboline alkaloids (such as harmicine (**1**), Fig. 1) continue to rely on the classical Pictet- Spengler and Bischler–Napieralski reactions and their variations. Discovered as early as 1893, the Bischler–Napieralski reaction^8^ (together with its contemporary variations) is still an object of intensive study in natural product synthesis.^9^

Following our interest in indole alkaloids and related com-pounds,^10^ we recently discovered that reaction of styrylacetamides **4** under typical Bischler–Napieralski conditions (POCl_3_, MeCN, Δ) leads to near-quantitative formation of carbazoles **5** instead of the expected dihydro-β-carbolines (Scheme 1A).^11^ Our ensuing mechanistic investigation revealed a highly complex cascade pathway (Scheme 2) which proceeds *via* several intriguing intermediates.^11^ In particular, the tetracyclic spiroindoline **10** caught our interest, owing to its wide-spread presence in indole alkaloids.^7*i*^ Unfortunately, our efforts to isolate **10** (R^1^ = H) were futile, frustrated by an elimination step (Scheme 2A) that always follows. Indeed, we only succeeded in diverting the cascade towards a different carbazole product if R_3_ = Br (**6**, Scheme 1A). This strongly suggests that interrupting the cascade at the stage of tetracyclic scaffold **10** is challenging due to the high thermodynamic driving force for the system to evolve towards aromatic products. Moreover, the intermediate **10** could revert back to **9** by retro-Mannich reaction and subsequently undergo Plancher rearrangement, irreversibly leading to β-carbolines.^12^

**Scheme 1:**
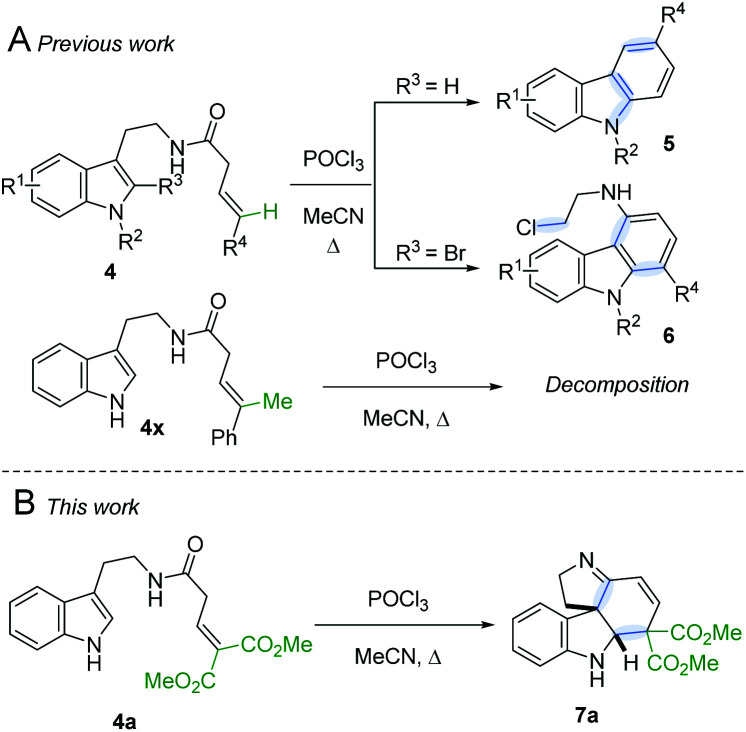
A. Previously reported carbazole formation B. Cascade interrupted at the tetracyclic intermediate.

**Scheme 2:**
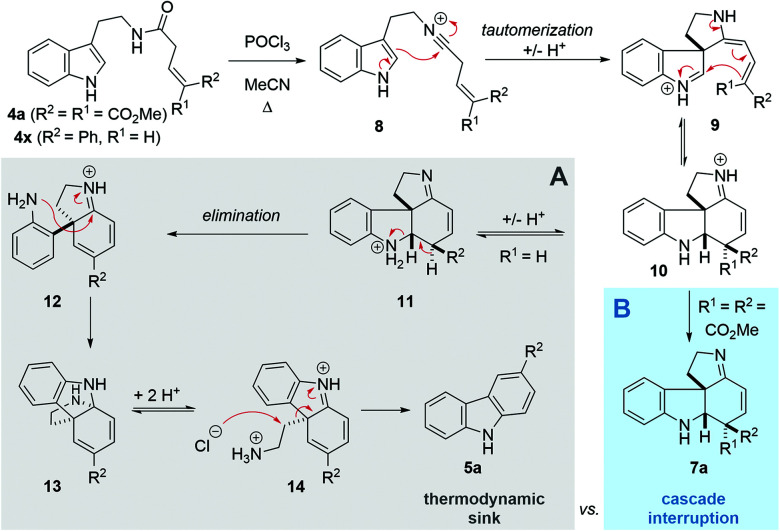
Previous cascade mechanism towards carbazoles (A) and interruption at the tetracyclic intermediate (B).

A potentially viable strategy to achieve the desired interruption would be to employ a γ,γ-disubstituted vinylacetamide as the substrate, as the elimination cannot occur in this case. Since the introduction of a methyl group at this position only led to decomposition (Scheme 1A), and given our success in employing ester R^4^ substituents,^11^ we identified malonate-derived tryptamide **4a** as a promising substrate. To our delight, upon subjecting **4a** to the cyclization conditions (Scheme 1B), we were able to isolate imine **7a** in 27% yield after basic workup. Optimization of the conditions (Table 1) showed that only POCl_3_ is able to promote the reaction (entries 1–5). Conveniently, we could avoid the strictly anhydrous conditions required for (both reactions and storage of) the highly reactive Tf_2_O that is often employed for related transformations.^9*a*–*d*^

**Table 1:** Reaction optimization

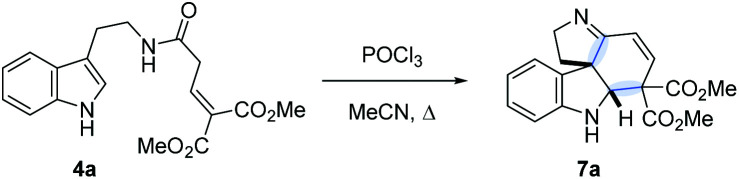				
Entry^a^	Reagent	Solvent	*T* (°C)	Yield^b^ (%)
1	POCl_3_	MeCN	Reflux	27^c^
2	Tf_2_O/2-Cl-Py	MeCN	Reflux	—
3	Tf_2_O/3-CN-Py	MeCN	Reflux	—
4	Ac_2_O	MeCN	Reflux	—
5	TFAA	MeCN	Reflux	—
6	POCl_3_	Toluene	90	16
7^d^	POCl_3_	MeCN	Reflux	20
8^e^	POCl_3_	MeCN	Reflux	18
9^f^	POCl_3_	MeCN	Reflux	37
10^g^	POCl_3_	MeCN	Reflux	58^c^
11^h^	POCl_3_	MeCN	Reflux	48
12^g^	POCl_3_	MeCN^i^	Reflux	58^c^

a Reaction conditions **4a** (0.2 mmol), reagent (0.3 mmol) solvent (1 mL).

b Determined by ^1^H NMR using 2,5-dimethylfuran as an internal standard.

c Isolated yield.

d Performed with 0.4 mmol of POCl_3_.

e Performed with 0.5 mmol of POCl_3_.

f Additional POCl_3_ (0.1 mmol) added after 30 min (1 h total reaction time).

g Additional POCl_3_ (0.1 mmol) added after 30 min and 1 h (1.5 h total reaction time).

h Additional POCl_3_ (0.1 mmol) added after 30 min, 1 h and 1.5 h (2 h total reaction time).

i Non-anhydrous MeCN was used.

Small adjustments of POCl_3_ stoichiometry and reaction time (entries 6–10) allowed us to increase the isolated yield of **7a** to 58%. The reaction proceeded with nearly identical efficiency on a 1.5 g scale.

We then focused on the generality of the process, subjecting differently substituted amide precursors to the optimized conditions (Scheme 3). Substituents on the benzenoid ring do not significantly affect the reactivity: reaction of the 5-F- and 5-MeO-substituted tryptamides afforded the corresponding tetracyclic scaffolds **7b** and **7d** in nearly identical yield (51–52%). The best result was obtained using the Cl-substituted amide **4c**, producing the tetracyclic product **7c** in 66% yield. Ethyl esters are also well tolerated, affording the desired product **7e** in the same yield as the benchmark product **7a**. The introduction of an N1-substituent on the indole ring proved to be beneficial, possibly because of the more reactive iminium ion intermediate (*cf.***9**, Scheme 2) favoring the cyclization step. Substituents on the R^5^ position are also tolerated: amide **4g**, derived from tryptophanol, was converted to **7g** in good yield (54%) as a single diastereomer. Replacing the ethylene linker with an *ortho*-phenylene linker also furnished the desired spiro product **7h**, albeit in lower yield (24%). Gratifyingly, the homotryptamine derivative **4i** was smoothly converted to the corresponding tetracycle **7i** in 55% yield. Interestingly, when substituents are present on the C2 position, a different type of product was observed (**15j–l**). Indeed, when R^3^ ≠ H, the C2-position of the indole becomes too hindered to undergo ring closure. In this case, enamine **9** can only undergo cycloaromatization (after *E*/*Z* isomerization) by attack on one of the two ester moieties to give the corresponding 2-pyridones **15**. Curiously, when R^3^ = Me, we observed the highest yield (**15j**, 89%), possibly owing to the higher stability of the product. On the other hand, the bromide-substituted product **15k** was obtained in lower yield. This is hardly surprising, considering the typical lability of imidoyl bromides. Similarly, the intriguing, but rather strained polycycle **15l** was isolated in 59% yield. Indeed, for both **15k** and **15l** decomposition was already observed during purification, accounting for the lower yields.

**Scheme 3:**
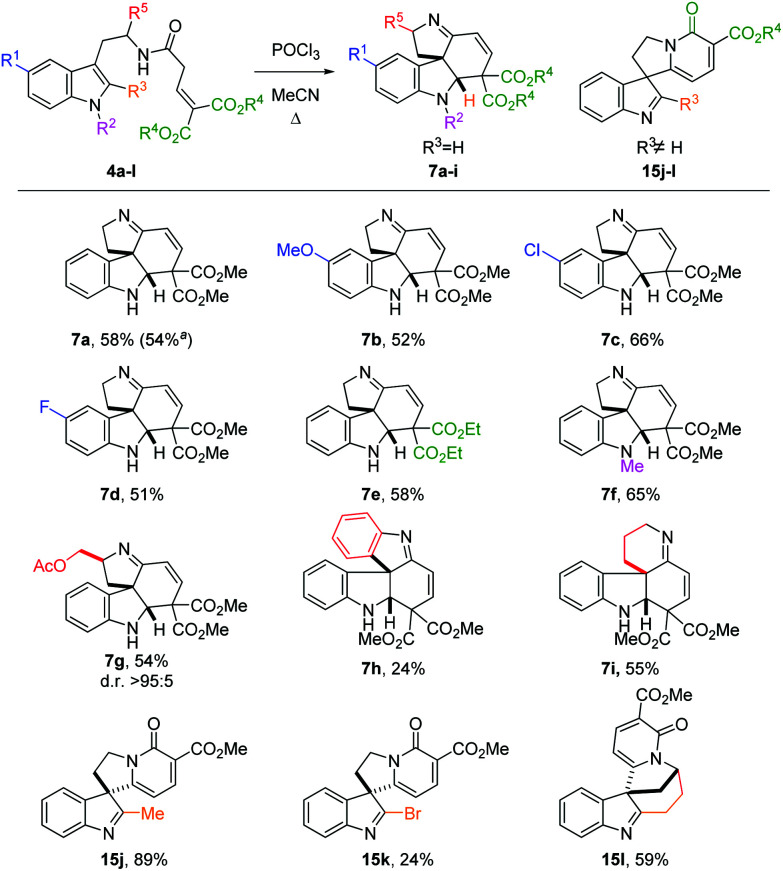
Scope of the cyclization. Reaction conditions: **4** (0.2 mmol), POCl_3_ (0.3 mmol), MeCN (1.0 mL), 1.5 h, reflux. Additional portions of POCl_3_ (0.1 mmol) were added after 30 min and 1 h. ^*a*^Reaction performed on 4.49 mmol scale.

Once we established the scope and limitation of this trans-formation, we set out to investigate the utility of these valuable intermediates in the total synthesis of indole alkaloids. Notably, we envisioned the transformation of **7a** to akuammicine (Scheme 4). Unfortunately, **7a** proved recalcitrant towards selective 1,2-reduction under various conditions (NaBH_4_, LiBH_4_, NaBH_3_CN, NaHB(OAc)_3_, or Et_3_SiH, both in the presence and absence of acidic promotors). Presumably, the conjugated imine is too rigid and/or sterically congested to undergo efficient 1,2-reduction. To circumvent this issue, we first performed a conjugate addition with thiophenol to give imine **16**, which could then be smoothly reduced by treatment with NaBH_4_ to give the desired amine **17** in 86% yield. Subsequent alkylation with **18** afforded the tertiary amine **19**. In order to reinstall the double bond, the thioether was first oxidized and then eliminated in a two-step procedure, producing the olefin **20** in 81% yield. Subsequently, the use of KOH (1.0 equiv.) in a MeOH/THF/H_2_O mixture at 0 °C afforded **21***via* one-pot saponification, decarboxylation and double bond migration.^13^ Finally, known intermediate **21** was converted to (±)-akuammicine (**3**) by intramolecular Heck reaction as reported previously.^14^

**Scheme 4:**
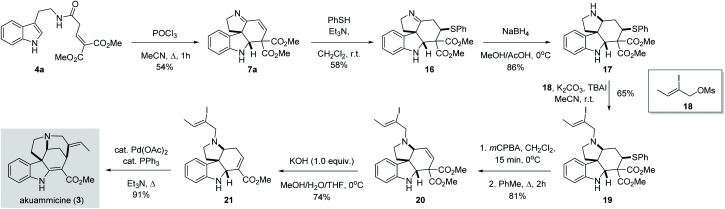
Total synthesis of (±)-akuammicine.

In conclusion, we report an alternative, interrupted variation of our previously serendipitously discovered diverted Bischler–Napieralski cascade reaction. The reaction generates complex polycyclic scaffolds in a single step and is compatible with a wide range of substituents, allowing straightforward access to highly functionalized and versatile intermediates. Moreover, tetracyclic indoline **7a** could be converted in only six steps to akuammicine, constituting a very short and efficient total synthesis of this Strychnos-type alkaloid. The scope and variability of the interrupted Bischler–Napieralski cyclization likely allow access to various other natural products as well.

## Conflicts of interest

The authors declare no competing financial interest.

## Supplementary Material

OB-019-D1OB01966J-s001
