# Supplementary material for: Synthesis of tetracyclic spiroindolines by an interrupted Bischler–Napieralski reaction: total synthesis of akuammicine
Source: Org Biomol Chem. 2021 Oct 27;19(44):9641–4. doi: 10.1039/d1ob01966j (PMC8600370; doi:10.1039/d1ob01966j)
Supplement: OB-019-D1OB01966J-s001 [file OB-019-D1OB01966J-s001.pdf]

Electronic Supplementary Material (ESI) for Organic & Biomolecular Chemistry.
This journal is © The Royal Society of Chemistry 2021

Supplementary Information

Synthesis of Tetracyclic Spiroindolines by an Interrupted Bischler-Napieralski Reaction: Total Synthesis of Akuammicine

Matteo Faltracco and Eelco Ruijter*

Table of contents

Optimization 3

General procedures 4

Compound Characterization 5

NMR data 12

References 54

General methods

Commercially available reagents were purchased from Sigma-Aldrich, Fischer, Strem Chemicals or Fluorochem and were used as purchased unless mentioned otherwise. Solvents were purchased from VWR Chemicals or Sigma-Aldrich and used without purification, unless stated otherwise. Anhydrous, air-free solvents were obtained from a PureSolv MD 5 solvent purification system. Infrared (IR) spectra were recorded neat using a Shimadzu FTIR-8400s spectrophotometer and wavelengths are reported in cm-1. Nuclear magnetic resonance (NMR) spectra were recorded on a Bruker Avance 600, Bruker Avance 500 or Bruker Avance 300 using the residual $CHCl_3$ as internal standard (^{1}H: δ 7.26 ppm, $^{13}C\{1H\}$: δ 77.16 ppm). Chemical shifts (δ) are given in ppm and coupling constants (J) are quoted in hertz (Hz). Resonances are described as s (singlet), d (doublet), t (triplet), q (quartet), br (broad singlet) and m (multiplet) or combinations thereof. Electrospray Ionization (ESI) high-resolution mass spectrometry was carried out using a Bruker micrOTOF-Q instrument in positive ion mode (capillary potential of 4500 V). Flash chromatography was performed on Silicycle Silica-P Flash Silica Gel (particle size 40-63 µm, pore diameter 60 Å) using the indicated eluent. Thin Layer Chromatography (TLC) was performed using TLC plates from Merck (SiO_2, Kieselgel 60 F254 neutral, on aluminium with fluorescence indicator) and compounds were visualized by UV detection (254 nm) and/or $KMnO_4$ stain. Mass spectrometry analyses were performed using a Shimadzu LCMS-2020 mass spectrometer. The data were acquired in full-scan APCI mode (MS) from m/z 100 to 800 in positive ionisation mode. Data was processed using Shimadzu Labsolutions 5.82.

General procedures

Synthesis of 3-bromoacrylic acid

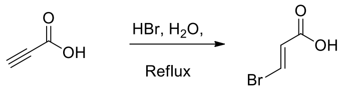

Propiolic acid (6.2 mL, 100.00 mmol, 1.0 eq.) was desolved in aqueous HBr (48%, 39.5 mL) and refluxed for 1h. The mixture was then slowly cooled to room temperature, then to 0°C. The white crystals were filtered and washed with cold water, dried, and used without further purification (Y: 92%, 13.894 g, 92.0 mmol).

Synthesis of amide precursors I (GP-A)

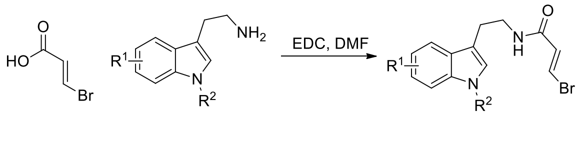

To a solution of 3-bromoacrylic acid (754.8 mg, 5.00 mmol, 1.00 eq.) in DMF (0.3 M, 16.7 mL), EDC (1006.4 mg, 5.25 mmol, 1.05 eq) was added at 0°C. After 5 min, the corresponding tryptamine (5.00 mmol, 1.00 eq.) was added ,and the reaction was stirred overnight at room temperature. The solvent was removed under reduced pressure and the crude was desolved in EtOAc (50 mL) and HCl 1M (20 mL). The organic layer was then washed with brine (7x20 mL), dried over Na_2SO_4, the solvent was removed under reduced pressure and the crude was purified by silica gel column chromatography as described in the corresponding synthetic procedure.

Synthesis of amide precursors II (GP-B)

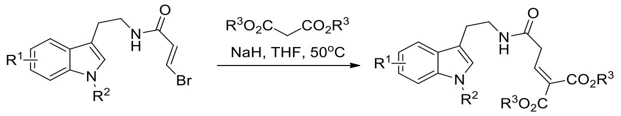

To a solution malonate (1.40 mmol, 1.40 eq.) in THF (3.3 mL, 0.3 M), 60% sodium hydride dispersion on mineral oil (56.0 mg, 1.40 mmol, 1.40 eq.) was added at 0 °C and kept at this temperature for 15 min. Then, a solution of amide (1.00 mmol, 1.0 eq.) in THF 15 (3.3 mL, 0.3 M) was added slowly, and the mixture was stirred at 50 °C for 6 hours. Once cooled to 0 °C, EtOAc (10 mL) and HCl 1M (5 mL) were added and the aqueous layer was extracted with EtOAc (2x10 mL). The combined organic layers were dried over Na_2SO_4, the solvent was removed under reduced pressure and the crude product was purified by silica gel column chromatography as described in the corresponding synthetic procedure.

Synthesis of tetracyclic scaffolds (GP-C)

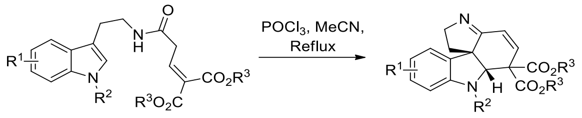

To a solution of amide (0.2 mmol, 1.0 eq.) in acetonitrile (1.0 mL, 0.2 M) was added $POCl_3$ (27 µL, 0.3 mmol, 1.5 eq.) and the mixture was refluxed adding $POCl_3$ (9 µL, 0.1 mmol, 0.5 eq.) after 30 min and 1h. After 30 more min, the mixture was cooled to 0°C, diluted with EtOAc (10 mL) and a saturated solution of Na_2CO_3 (10 mL) was added. The aqueous layer was extracted with EtOAc (2x10 mL) and the combined organic layers were dried over Na_2SO_4, the solvent was removed under reduced pressure and the crude product was purified by silica gel column chromatography as described in the corresponding synthetic procedure.

Compound Characterization

(*E*)-N-(2-(1H-indol-3-yl)ethyl)-3-bromoacrylamide (S1a).

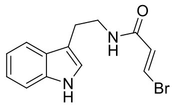

Prepared according to **GP-A (18.7 mmol scale)**. Purification of the crude material was performed by silica gel column chromatography (30% EtOAc/*c*Hex) providing the title compound as a white solid in 24% yield (1.316 g, 4.49 mmol). **R_F** = 0.72 (50% EtOAc/*c*Hex). **^{1}H NMR** (500 MHz, $CDCl_3$) δ 8.13 (s, 1H), 7.62 (dd, J = 7.9, 1.1 Hz, 1H), 7.46 (d, J = 13.4 Hz, 1H), 7.41 (dt, J = 8.1, 1.0 Hz, 1H), 7.28 – 7.21 (m, 1H), 7.16 (ddd, J = 8.0, 7.0, 1.0 Hz, 1H), 7.06 (d, J = 2.3 Hz, 1H), 6.38 (d, J = 13.4 Hz, 1H), 5.58 (s, 1H), 3.68 (q, J = 6.5 Hz, 2H), 3.03 (t, J = 6.6 Hz, 2H). **^{13}C NMR** (126 MHz, $CDCl_3$) δ 163.5, 136.6, 131.0, 127.4, 122.7, 122.5, 122.2, 119.8, 118.8, 112.8, 111.5, 40.0, 25.3. **HRMS (ESI)**: *m/z* calculated for $C_{13}H_{14}N_2OBr^+$ $[M+H]^+$ 293.0284, found 293.0278.

(*E*)-3-bromo-N-(2-(5-methoxy-1H-indol-3-yl)ethyl)acrylamide (S1b).

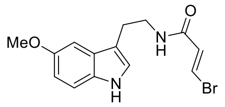

Prepared according to **GP-A**. Purification of the crude material was performed by silica gel column chromatography (40% EtOAc/*c*Hex) providing the title compound as a white solid in 33% yield (533.2 mg, 1.65 mmol). **R_F** = 0.45 (50% EtOAc/*c*Hex). **^{1}H NMR** (500 MHz, $CDCl_3$) δ 7.98 (s, 1H), 7.45 (d, J = 13.4 Hz, 1H), 7.27 (d, J = 9.0 Hz, 1H), 7.03 – 7.01 (m, 2H), 6.88 (dd, J = 8.8, 2.4 Hz, 1H), 6.37 (d, J = 13.4 Hz, 1H), 5.56 (s, 1H), 3.86 (s, 3H), 3.65 (d, J = 6.6 Hz, 2H), 2.98 (t, J = 6.6 Hz, 2H). **^{13}C NMR** (126 MHz, $CDCl_3$) δ 163.5, 154.3, 131.6, 131.0, 127.8, 123.0, 122.7, 112.8, 112.6, 112.2, 100.5, 56.0, 40.0, 25.3. **HRMS (ESI)**: *m/z* calculated for $C_{14}H_{16}N_2O_2Br^+$ $[M+H]^+$ 323.0390, found 323.0401.

(*E*)-3-bromo-N-(2-(5-chloro-1H-indol-3-yl)ethyl)acrylamide (S1c).

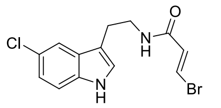

Prepared according to **GP-A**. Purification of the crude material was performed by silica gel column chromatography (35% EtOAc/*c*Hex) providing the title compound as a white solid in 32% yield (524.2 mg, 1.60 mmol). **R_F** = 0.25 (33% EtOAc/*c*Hex). **^{1}H NMR** (500 MHz, $CDCl_3$) δ 8.11 (s, 1H), 7.55 (d, J = 1.9 Hz, 1H), 7.46 (d, J = 13.4 Hz, 1H), 7.30 (dd, J = 8.6, 0.6 Hz, 1H), 7.17 (dd, J = 8.6, 2.0 Hz, 1H), 7.07 (d, J = 2.3 Hz, 1H), 6.39 (d, J = 13.4 Hz, 1H), 5.52 (s, 1H), 3.64 (q, J = 6.7 Hz, 2H), 2.97 (t, J = 6.7 Hz, 2H). **^{13}C NMR** (126 MHz, $CDCl_3$) δ 163.6, 134.8, 130.9, 128.5, 125.6, 123.6, 122.9, 122.8, 118.4, 112.7, 112.5, 39.9, 25.29. **HRMS (ESI)**: *m/z* calculated for $C_{13}H_{13}N_2OClBr^+$ $[M+H]^+$ 326.9894, found 326.9903.

(*E*)-3-bromo-N-(2-(5-fluoro-1H-indol-3-yl)ethyl)acrylamide (S1d-S1d').

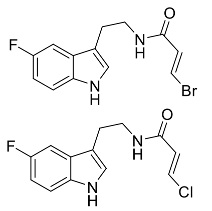

Prepared according to **GP-A**. Purification of the crude material was performed by silica gel column chromatography (40% EtOAc/*c*Hex) providing the title compound as a white solid in 23% yield (524.2 mg, 1.15 mmol). **R_F** = 0.49 (50% EtOAc/*c*Hex). The product is present as a 1:1 mixture with the corresponding chloro-acrylamide. All signals are herby reported. **^{1}H NMR** (500 MHz, $CDCl_3$) δ 8.35 (s, 2H), 7.43 (d, J = 13.4 Hz, 1H), 7.30 – 7.24 (m, 3H), 7.20 (dd, J = 9.6, 2.5 Hz, 2H), 7.05 (s, 2H), 6.95 (td, J = 9.0, 2.5 Hz, 2H), 6.40 (d, J = 13.4 Hz, 1H), 6.13 (d, J = 13.1 Hz, 1H), 5.89 – 5.72 (m, 2H), 3.65 – 3.57 (m, 4H), 2.94 (t, J = 6.8 Hz, 4H). **^{13}C NMR** (126 MHz, $CDCl_3$) δ 163.7, 163.5, 157.9 (d, J = 235.0 Hz, 2C), 134.1, 133.0 (2C), 131.0, 127.73 (d, J = 0.9 Hz), 127.65 (d, J = 0.9 Hz), 126.9, 124.0 (2C), 122.8, 112.84 (d, J = 5.3 Hz), 112.82 (d, J = 5.3 Hz), 112.1 (d, J = 9.6 Hz, 2C), 110.8 (d, J = 26.3 Hz, 2C), 103.6 (d, J = 23.4 Hz, 2C), 39.8 (2C), 25.23, 25.20. **HRMS (ESI) (Br)**: *m/z* calculated for $C_{13}H_{13}N_2OFBr^+$ $[M+H]^+$ 311.0190, found 311.0206. **HRMS (ESI) (Cl)**: *m/z* calculated for $C_{13}H_{13}N_2OFCl^+$ $[M+H]^+$ 267.0695, found 267.0704.

(*E*)-3-bromo-N-(2-(1-methyl-1H-indol-3-yl)ethyl)acrylamide (S1e).

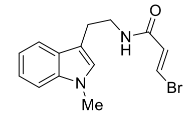

Prepared according to **GP-A**. Purification of the crude material was performed by silica gel column chromatography (30% EtOAc/*c*Hex) providing the title compound as a green solid in 22% yield (337.9 mg, 1.10 mmol). **R_F** = 0.58 (50% EtOAc/*c*Hex). **^{1}H NMR** (500 MHz, $CDCl_3$) δ 7.58 (dt, J = 7.9, 1.0 Hz, 1H), 7.44 (d, J = 13.4 Hz, 1H), 7.32 (dt, J = 8.3, 0.9 Hz, 1H), 7.27 – 7.24 (m, 1H), 7.13 (ddd, J = 8.0, 6.9, 1.1 Hz, 1H), 6.89 (s, 1H), 6.37 (d, J = 13.4 Hz, 1H), 5.79 (t, J = 7.0 Hz, 1H), 3.76 (s, 3H), 3.62 (q, J = 6.5 Hz, 2H), 2.98 (t, J = 6.7 Hz, 2H). **^{13}C NMR** (126 MHz, $CDCl_3$) δ 163.6, 137.2, 131.0, 127.7, 127.0, 122.6, 122.0, 119.2, 118.8, 111.2, 109.5, 40.1, 32.8, 25.1. **HRMS (ESI)**: *m/z* calculated for $C_{14}H_{16}N_2OBr^+$ $[M+H]^+$ 307.0441, found 307.0454.

(*E*)-2-(3-bromoacrylamido)-3-(1H-indol-3-yl)propyl acetate (S1f).

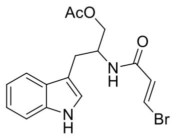

Prepared according to **GP-A**. Purification of the crude material was performed by silica gel column chromatography (30% EtOAc/*c*Hex) providing the title compound as a white solid in 35% yield (639.1 mg, 1.75 mmol). **R_F** = 0.50 (50% EtOAc/*c*Hex). **^{1}H NMR** (500 MHz, $CDCl_3$) δ 8.26 (s, 1H), 7.64 (d, J = 8.0 Hz, 1H), 7.46 (d, J = 13.4 Hz, 1H), 7.36 (dt, J = 8.1, 0.9 Hz, 1H), 7.21 (ddd, J = 8.1, 7.0, 1.2 Hz, 1H), 7.14 (ddd, J = 8.0, 7.0, 1.1 Hz, 1H), 7.00 (d, J = 2.3 Hz, 1H), 6.43 (d, J = 13.5 Hz, 1H), 5.90 (d, J = 8.5 Hz, 1H), 4.61 – 4.53 (m, 1H), 4.14 (dd, J = 11.4, 6.0 Hz, 1H), 4.07 (dd, J = 11.4, 4.5 Hz, 1H), 3.08 (dd, J = 14.7, 5.5 Hz, 1H), 2.99 (dd, J = 14.7, 7.6 Hz, 1H), 2.08 (s, 3H). **^{13}C NMR** (126 MHz, $CDCl_3$) δ 171.4, 163.4, 136.3, 130.9, 127.6, 123.1, 122.9, 122.4, 119.9, 118.8, 111.4, 110.7, 64.9, 49.2, 26.9, 21.0. **HRMS (ESI)**: *m/z* calculated for $C_{16}H_{18}N_2O_3Br^+$ $[M+H]^+$ 365.0495, found 365.0512.

(*E*)-N-(2-(1H-indol-3-yl)phenyl)-3-bromoacrylamide (S1g).

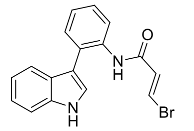

Prepared according to **GP-A** (1 mmol scale). Purification of the crude material was performed by silica gel column chromatography (10% EtOAc/*c*Hex) providing the title compound as a white foam in 98% yield (334.4 mg, 0.98 mmol). **R_F** = 0.54 (20% EtOAc/*c*Hex). **^{1}H NMR** (600 MHz, $CDCl_3$) δ 8.72 (s, 1H), 8.47 (d, J = 7.6 Hz, 1H), 7.54 (s, 1H), 7.49 (d, J = 8.2 Hz, 1H), 7.47 – 7.43 (m, 2H), 7.42 – 7.38 (m, 2H), 7.30 (ddd, J = 8.2, 7.0, 1.1 Hz, 1H), 7.25 (d, J = 2.5 Hz, 1H), 7.22 (t, J = 7.5 Hz, 1H), 7.19 – 7.16 (m, 1H), 6.23 (d, J = 13.4 Hz, 1H). **^{13}C NMR** (151 MHz, $CDCl_3$) δ 161.5, 136.4, 135.4, 131.5, 131.2, 128.1, 126.0, 125.2, 124.7, 123.72, 123.68, 123.2, 121.1, 120.9, 119.6, 113.0, 111.9. **HRMS (ESI)**: *m/z* calculated for $C_{17}H_{14}N_2OBr^+$ $[M+H]^+$ 341.0284, found 341.0301.

(*E*)-N-(3-(1H-indol-3-yl)propyl)-3-bromoacrylamide (S1h).

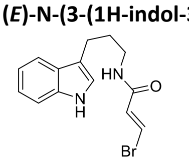

Prepared according to **GP-A**. Purification of the crude material was performed by silica gel column chromatography (40% EtOAc/*c*Hex) providing the title compound as a white solid in 25% yield (384.0 mg, 1.25 mmol). **R_F** = 0.44 (50% EtOAc/*c*Hex). **^{1}H NMR** (500 MHz, $CDCl_3$) δ 7.98 (s, 1H), 7.58 (dd, J = 7.9, 1.0 Hz, 1H), 7.39 – 7.33 (m, 2H), 7.21 (ddd, J = 8.1, 7.0, 1.2 Hz, 1H), 7.12 (ddd, J = 8.0, 7.1, 1.1 Hz, 1H), 7.02 (d, J = 2.4 Hz, 1H), 6.33 (d, J = 13.4 Hz, 1H), 5.41 (s, 1H), 3.39 (q, J = 6.8 Hz, 2H), 2.83 (t, J = 7.2 Hz, 2H), 1.97 (p, J = 7.1 Hz, 2H). **^{13}C NMR** (126 MHz, $CDCl_3$) δ 164.9, 136.5, 131.0, 127.3, 122.5, 122.3, 121.6, 119.5, 118.9, 115.5, 111.4, 39.7, 29.7, 22.8. **HRMS (ESI)**: *m/z* calculated for $C_{14}H_{16}N_2OBr^+$ $[M+H]^+$ 307.0441, found 307.0457.

(*E*)-3-bromo-N-(2-(2-methyl-1H-indol-3-yl)ethyl)acrylamide (S1i).

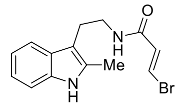

Prepared according to **GP-A**. Purification of the crude material was performed by silica gel column chromatography (30% EtOAc/*c*Hex) providing the title compound as a white solid in 41% yield (629.7 mg, 2.05 mmol). **R_F** = 0.36 (33% EtOAc/*c*Hex). **^{1}H NMR** (500 MHz, $CDCl_3$) δ 7.95 (s, 1H), 7.47 (d, J = 7.5 Hz, 1H), 7.42 (d, J = 13.4 Hz, 1H), 7.27 (dt, J = 7.9, 1.0 Hz, 1H), 7.14 (td, J = 8.1, 7.6, 1.3 Hz, 1H), 7.09 (td, J = 7.5, 7.1, 1.2 Hz, 1H), 6.30 (d, J = 13.4 Hz, 1H), 5.59 (d, J = 6.6 Hz, 1H), 3.55 (q, J = 6.5 Hz, 2H), 2.94 (t, J = 6.7 Hz, 2H), 2.36 (s, 3H). **^{13}C NMR** (126 MHz, $CDCl_3$) δ 163.6, 135.4, 132.2, 131.0, 128.6, 122.6, 121.4, 119.7, 117.8, 110.5, 108.3, 40.2, 24.0, 11.7. **HRMS (ESI)**: *m/z* calculated for $C_{14}H_{16}N_2OBr^+$ $[M+H]^+$ 307.0441, found 307.0450.

(*E*)-3-bromo-N-(2-(2-bromo-1H-indol-3-yl)ethyl)acrylamide (S1j).

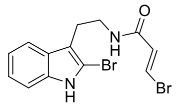

To a solution of amide **20a** (1465.8 mg, 5.0 mmol, 1.0 eq.) in a 1:1 mixure of DCM:THF (20 mL, 0.25M) was cooled to 0°C. Then, piridinium tribromide (1918.9 mg, 6.0 mmol, 1.2 eq.) was added slowly and stirred for 2h at the same temperature. A saturated solution (100 mL) of Na_2SO_3 and $NaHCO_3$ (1:1) was added and the aqueous phase was extracted with DCM (2x50 mL); the combined organic layers were dried over Na_2SO_4 and the solvent was removed under reduced pressure. Purification of the crude material was performed by silica gel column chromatography (60% EtOAc/*c*Hex) providing the title compound as a red oil in 61% yield (1134.8 mg, 3.05 mmol). **R_F** = 0.78 (50% EtOAc/*c*Hex). **^{1}H NMR** (500 MHz, $CDCl_3$) δ 8.25 (s, 1H), 7.51 (d, J = 7.9 Hz, 1H), 7.45 (d, J = 13.4 Hz, 1H), 7.31 (dt, J = 8.1, 0.9 Hz, 1H), 7.20 (ddd, J = 8.2, 7.2, 1.2 Hz, 1H), 7.13 (ddd, J = 8.1, 7.1, 1.0 Hz, 1H), 6.36 (d, J = 13.4 Hz, 1H), 5.59 (s, 1H), 3.61 (q, J = 6.4 Hz, 2H), 2.98 (t, J = 6.6 Hz, 2H). **^{13}C NMR** (126 MHz, $CDCl_3$) δ 163.7, 136.3, 130.9, 127.7, 122.8 (2C), 120.6, 118.1, 112.4, 110.8, 108.9, 39.6, 24.8. **HRMS (ESI)**: *m/z* calculated for $C_{13}H_{13}N_2OBr_2^+$ $[M+H]^+$ 370.9389, found 370.9402.

(*E*)-3-bromo-N-(2,3,4,9-tetrahydro-1H-carbazol-3-yl)acrylamide (S1k).

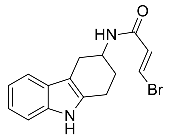

Prepared according to **GP-A**. Purification of the crude material was performed by silica gel column chromatography (30% EtOAc/*c*Hex) providing the title compound as a white solid in 14% yield (223.4 mg, 0.70 mmol). **R_F** = 0.62 (50% EtOAc/*c*Hex). **^{1}H NMR** (500 MHz, $CDCl_3$) δ 7.87 (s, 1H), 7.47 (d, J = 13.4 Hz, 1H), 7.43 (d, J = 7.7 Hz, 1H), 7.30 (d, J = 8.0 Hz, 1H), 7.16 (td, J = 7.1, 1.0 Hz, 1H), 7.10 (td, J = 7.1, 1.0 Hz, 1H), 6.42 (d, J = 13.4 Hz, 1H), 5.70 (d, J = 7.4 Hz, 1H), 4.54 – 4.47 (m, 1H), 3.10 (ddt, J = 15.5, 5.1 Hz, 1.6 Hz, 1H), 2.89 – 2.83 (m, 1H), 2.78 (dt, J = 15.0, 6.5 Hz, 1H), 2.66 (dd, J = 15.3, 6.0 Hz, 1H), 2.12 – 2.05 (m, 2H). **^{13}C NMR** (126 MHz, $CDCl_3$) δ 163.2, 136.2, 132.8, 131.1, 127.6, 122.9, 121.8, 119.6, 117.8, 110.7, 107.1, 45.4, 27.8, 27.7, 20.4. **HRMS (ESI)**: *m/z* calculated for $C_{15}H_{16}N_2OBr^+$ $[M+H]^+$ 319.0441, found 319.0452.

(*E*)-N-(2-(1H-indol-3-yl)ethyl)-3-bromoacrylamide (4a).

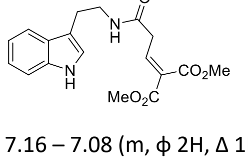

Prepared according to **GP-B (6.735 mmol scale).** Purification of the crude material was performed by silica gel column chromatography (50% EtOAc/*c*Hex) providing the title compound as a white foam in 52% yield (804.1 mg, 2.34 mmol). **R_F** = 0.31 (60% EtOAc/*c*Hex). The product is present at the NMR as a mixture of two tautomers in a 1:0.4 ratio, the major one will be indicated with ϕ the minor one will be indicated with Δ. **^{1}H NMR** (500 MHz, $CDCl_3$) δ 8.02 (s, ϕ 1H, Δ 1H), 7.57 – 7.46 (m, ϕ 1H, Δ 1H), 7.35-7.23 (m, ϕ 1H, Δ 1H), 7.16 – 7.08 (m, ϕ 2H, Δ 1H), 7.06 – 7.02 (m, ϕ 1H, Δ 1H), 6.96 (d, J = 2.2 Hz, Δ 1H), 6.94 (d, J = 2.2 Hz, ϕ 1H), 6.82 (dd, J = 15.4, 8.7 Hz, Δ 1H), 6.03 (s, ϕ 1H), 5.83 (dd, J = 15.4, 1.1 Hz, Δ 1H), 5.59 (s, Δ 1H), 4.07 (dd, J = 8.3, 1.1 Hz, Δ 1H), 3.72 (s, ϕ 3H), 3.68 (s, ϕ 3H), 3.67 (s, Δ 3H), 3.62 (s, Δ 3H), 3.60 (q, J = 6.3 Hz, Δ 2H) 3.51 (q, J = 6.3 Hz, ϕ 2H), 3.15 (d, J = 8.1 Hz, ϕ 2H), 2.93 (t, J = 6.7 Hz, Δ 2H), 2.90 (t, J = 6.7 Hz, ϕ 2H). **^{13}C NMR** (126 MHz, $CDCl_3$) δ Δ 168.9, Δ 168.6, ϕ 167.6, Δ 167.2, ϕ 165.42, ϕ 164.39, ϕ 144.0, ϕΔ 136.4, Δ 133.3, ϕΔ 129.6, Δ 128.7, ϕ 127.3, Δ 122.3, ϕ 122.22, ϕ 122.19, Δ 122.16, ϕ 119.6, Δ 119.5, Δ 118.73, ϕ 118.70, Δ 112.84, ϕ 112.75, Δ 111.3, ϕ 111.2, Δ 54.3, Δ 52.8, ϕ 52.59, ϕ 52.56, Δ 52.3, ϕ 40.0, Δ 39.8, ϕ 37.9, Δ 25.2, ϕ 25.1. **HRMS (ESI)**: *m/z* calculated for $C_{18}H_{21}N_2O_5^+$ $[M+H]^+$ 345.1145, found 345.1153.

dimethyl 2-(3-((2-(5-methoxy-1H-indol-3-yl)ethyl)amino)-3-oxopropylidene)malonate (4b).

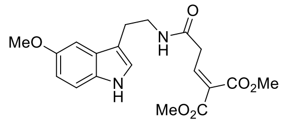

Prepared according to **GP-B**. Purification of the crude material was performed by silica gel column chromatography (50% EtOAc/*c*Hex) providing the title compound as a white solid in 57% yield (213.4 mg, 0.57 mmol). $\mathbf{R_F}$ = 0.42 (66% EtOAc/*c*Hex). The product is present at the NMR as a mixture of two tautomers in a 1:0.4 ratio, the signal of the major one are reported. **^{1}H NMR** (500 MHz, $CDCl_3$) δ 8.02 (s, 1H), 7.26 – 7.20 (m, 2H), 7.02 (d, J = 2.4 Hz, 1H), 6.99 (d, J = 1.7 Hz, 1H), 6.86 (dd, J = 8.8, 2.5 Hz, 1H), 6.15 (d, J = 6.8 Hz, 1H), 3.86 (s, 3H), 3.79 (s, 3H), 3.77 (s, 3H), 3.58 (q, J = 6.8 Hz, 2H), 3.24 (d, J = 8.1 Hz, 2H), 2.94 (t, J = 6.7 Hz, 2H). **^{13}C NMR** (126 MHz, $CDCl_3$) δ 167.7, 165.6, 164.2, 154.2, 144.1, 131.6, 129.7, 127.7, 123.1, 112.6, 112.5, 112.1, 100.5, 56.1, 52.72, 52.70, 40.0, 38.0, 25.3. **HRMS (ESI)**: *m/z* calculated for $C_{19}H_{23}N_2O_6^+$ $[M+H]^+$ 375.1551, found 373.1564.

dimethyl 2-(3-((2-(5-chloro-1H-indol-3-yl)ethyl)amino)-3-oxopropylidene)malonate (4c).

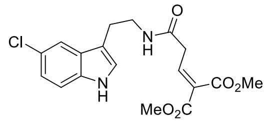

Prepared according to **GP-B**. Purification of the crude material was performed by silica gel column chromatography (40% EtOAc/*c*Hex) providing the title compound as a pale oil in 39% yield (147.7 mg, 0.39 mmol). $\mathbf{R_F}$ = 0.53 (66% EtOAc/*c*Hex). The product is present at the NMR as a mixture of two tautomers in a 1:0.4 ratio, the signal of the major one are reported. **^{1}H NMR** (500 MHz, $CDCl_3$) δ 8.35 (s, 1H), 7.54 (d, J = 2.0 Hz, 1H), 7.27 (d, J = 8.5 Hz, 1H), 7.22 (t, J = 8.1 Hz, 1H), 7.13 (dd, J = 8.6, 1.8 Hz, 1H), 7.02 (d, J = 2.2 Hz, 1H), 6.25 (d, J = 6.7 Hz, 1H), 3.78 (s, 3H), 3.77 (s, 3H), 3.55 (q, J = 6.7 Hz, 2H), 3.23 (d, J = 8.1 Hz, 2H), 2.91 (t, J = 6.9 Hz, 2H). **^{13}C NMR** (126 MHz, $CDCl_3$) δ 167.9, 167.4, 165.6, 144.1, 134.8, 129.7, 128.5, 125.3, 123.8, 122.5, 118.3, 112.6, 112.4, 52.7 (2C), 40.1, 38.0, 25.1. **HRMS (ESI)**: *m/z* calculated for $C_{18}H_{20}N_2ClO_5^+$ $[M+H]^+$ 379.1055, found 379.1061.

dimethyl 2-(3-((2-(5-fluoro-1H-indol-3-yl)ethyl)amino)-3-oxopropylidene)malonate (4d).

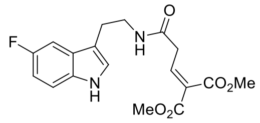

Prepared according to **GP-B**. Purification of the crude material was performed by silica gel column chromatography (60% EtOAc/*c*Hex) providing the title compound as a pale oil in 48% yield (173.9 mg, 0.48 mmol). $\mathbf{R_F}$ = 0.33 (66% EtOAc/*c*Hex). The product is present at the NMR as a mixture of two tautomers in a 1:0.4 ratio, the signal of the major one are reported. **^{1}H NMR** (500 MHz, $CDCl_3$) δ 8.42 (s, 1H), 7.29 – 7.24 (m, 1H), 7.22 – 7.16 (m, 2H), 7.03 (d, J = 2.0 Hz, 1H), 6.92 (td, J = 9.1, 2.6 Hz, 1H), 6.28 (t, J = 5.9 Hz, 1H), 3.78 (s, 3H), 3.76 (s, 3H), 3.54 (q, J = 6.8 Hz, 2H), 3.23 (d, J = 8.1 Hz, 2H), 2.90 (t, J = 6.9 Hz, 2H). **^{13}C NMR** (126 MHz, $CDCl_3$) δ 167.9, 167.4, 165.6, 157.8 (d, J = 234.7 Hz), 144.1, 133.0, 129.7, 127.7 (d, J = 9.6 Hz), 124.2, 112.8 (d, J = 4.7 Hz), 112.0 (d, J = 9.6 Hz), 110.5 (d, J = 26.3 Hz), 103.6 (d, J = 23.3 Hz), 52.70, 52.68, 40.1, 37.9, 25.2. **HRMS (ESI)**: *m/z* calculated for $C_{18}H_{20}N_2FO_5^+$ $[M+H]^+$ 363.1351, found 363.1368.

diethyl 2-(3-((2-(1H-indol-3-yl)ethyl)amino)-3-oxopropylidene)malonate (4e).

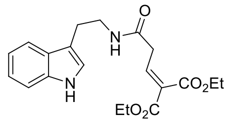

Prepared according to **GP-B**. Purification of the crude material was performed by silica gel column chromatography (40% EtOAc/*c*Hex) providing the title compound as a pale oil in 35% yield (130.3 mg, 0.35 mmol). $\mathbf{R_F}$ = 0.45 (66% EtOAc/*c*Hex). The product is present at the NMR as a mixture of two tautomers in a 1:0.4 ratio, the signal of the major one are reported. **^{1}H NMR** (600 MHz, $CDCl_3$) δ 8.38 (s, 1H), 7.58 (d, J = 7.9 Hz, 1H), 7.35 (d, J = 8.1 Hz, 1H), 7.20 – 7.14 (m, 2H), 7.10 (t, J = 7.4 Hz, 1H), 6.98 (s, 1H), 6.27 (t, J = 5.5 Hz, 1H), 4.29 – 4.15 (m, 4H), 3.57 (q, J = 6.5 Hz, 2H), 3.22 (d, J = 8.1 Hz, 2H), 2.97 (dt, J = 13.6, 6.8 Hz, 2H), 1.33 – 1.17 (m, 6H). **^{13}C NMR** (151 MHz, $CDCl_3$) δ 168.0, 165.2, 163.8, 142.9, 136.5, 130.5, 127.3, 122.4, 122.1, 119.4, 118.7, 112.6, 111.4, 61.8, 61.7, 40.2, 37.8, 25.2, 14.15, 14.12. **HRMS (ESI)**: *m/z* calculated for $C_{20}H_{25}N_2O_5^+$ $[M+H]^+$ 373.1758, found 373.1761.

dimethyl 2-(3-((2-(1-methyl-1H-indol-3-yl)ethyl)amino)-3-oxopropylidene)malonate (4f).

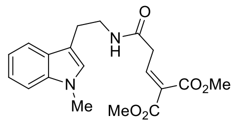

Prepared according to **GP-B**. Purification of the crude material was performed by silica gel column chromatography (50% EtOAc/*c*Hex) providing the title compound as a pale oil in 47% yield (168.4 mg, 0.47 mmol). $\mathbf{R_F}$ = 0.23 (50% EtOAc/*c*Hex). The product is present at the NMR as a mixture of two tautomers in a 1:0.4 ratio, the signal of the major one are reported. **^{1}H NMR** (500 MHz, $CDCl_3$) δ 7.60 – 7.57 (m, 1H), 7.34 – 7.29 (m, 1H), 7.28 – 7.22 (m, 2H), 7.12 (ddd, J = 8.0, 6.9, 1.1 Hz, 1H), 6.89 (s, 1H), 6.16 (t, J = 6.0 Hz, 1H), 3.81 (s, 3H), 3.79 (s, 3H), 3.76 (s, 3H), 3.57 (q, J = 6.8 Hz, 2H), 3.24 (d, J = 8.0 Hz, 2H), 2.97 (t, J = 6.8 Hz, 2H). **^{13}C NMR** (126 MHz, $CDCl_3$) δ 167.7, 165.5, 164.1, 144.1, 137.2, 129.7, 127.8, 127.0, 121.8, 119.0, 118.9, 111.2, 109.4, 52.65, 52.63, 40.3, 37.9, 32.7, 25.2. **HRMS (ESI)**: *m/z* calculated for $C_{19}H_{23}N_2O_5^+$ $[M+H]^+$ 359.1601, found 359.1614.

dimethyl 2-(3-((1-acetoxy-3-(1H-indol-3-yl)propan-2-yl)amino)-3-oxopropylidene)malonate (4g).

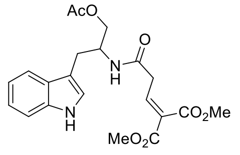

Prepared according to **GP-B**. Purification of the crude material was performed by silica gel column chromatography (50% EtOAc/*c*Hex) providing the title compound as a pale oil in 62% yield (258.2 mg, 0.62 mmol). $\mathbf{R_F}$ = 0.38 (66% EtOAc/*c*Hex). The product is present at the NMR as a mixture of two tautomers in a 1:0.6 ratio, the signal of the major one are reported. The minor tautomer is present as 1:1 mixture of rotamers. **^{1}H NMR** (500 MHz, $CDCl_3$) δ 8.20 (s, 1H), 7.64 (d, J = 7.9 Hz, 1H), 7.35 (d, J = 8.1 Hz, 1H), 7.23 – 7.17 (m, 2H), 7.12 (t, J = 7.5 Hz, 1H), 7.01 – 7.01 (m, 1H), 6.39 (d, J = 8.3 Hz, 1H), 4.52 – 4.46 (m, 1H), 4.12 – 4.07 (m, 2H), 3.80 (s, 3H), 3.80 (s, 3H), 3.29 – 3.20 (m, 2H), 3.06 – 2.97 (m, 2H), 2.08 (s, 3H). **^{13}C NMR** (126 MHz, $CDCl_3$) δ 171.2, 167.6, 165.7, 164.1, 143.9, 136.3, 129.8, 127.6, 122.9, 122.3, 119.8, 118.8, 111.3, 111.0, 64.9, 52.75, 52.72, 49.3, 38.0, 27.0, 20.9. **HRMS (ESI)**: *m/z* calculated for $C_{21}H_{25}N_2O_7^+$ $[M+H]^+$ 417.1656, found 417.1670.

dimethyl 2-(3-((2-(1H-indol-3-yl)phenyl)amino)-3-oxopropylidene)malonate (4h).

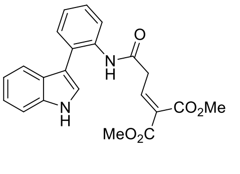

Prepared according to **GP-B**. Purification of the crude material was performed by silica gel column chromatography (30% EtOAc/*c*Hex) providing the title compound as a pale oil in 43% yield (168.7 mg, 0.43 mmol). **R_F** = 0.45 (50% EtOAc/*c*Hex). The product is present at the NMR as a mixture of two tautomers in a 1:0.3 ratio, the signal of the major one are reported. **^{1}H NMR** (500 MHz, $CDCl_3$) δ 8.72 (s, 1H), 8.33 (d, J = 8.1 Hz, 1H), 7.98 (s, 1H), 7.52 – 7.31 (m, 4H), 7.23 – 7.07 (m, 5H), 3.78 (s, 3H), 3.55 (s, 3H), 3.27 (d, J = 8.1 Hz, 2H). **^{13}C NMR** (126 MHz, $CDCl_3$) δ 165.8, 164.8, 164.2, 143.1, 136.4, 135.8, 131.2, 129.8, 127.9, 126.6, 125.4, 124.5, 123.8, 122.8, 121.3, 120.6, 119.6, 112.9, 111.6, 52.7, 52.5, 39.0. **HRMS (ESI)**: *m/z* calculated for $C_{22}H_{21}N_2O_5^+$ [M+H]$^+$ 393.1445, found 393.1460.

dimethyl 2-(3-((3-(1H-indol-3-yl)propyl)amino)-3-oxopropylidene)malonate (4i).

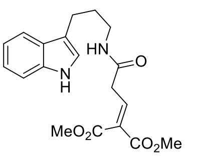

Prepared according to **GP-B**. Purification of the crude material was performed by silica gel column chromatography (40% EtOAc/*c*Hex) providing the title compound as a white solid in 56% yield (200.7 mg, 0.56 mmol). **R_F** = 0.52 (66% EtOAc/*c*Hex). The product is present at the NMR as a mixture of two tautomers in a 1:0.3 ratio, the signal of the major one are reported. **^{1}H NMR** (500 MHz, $CDCl_3$) δ 8.07 (s, 1H), 7.58 (d, J = 7.7 Hz, 1H), 7.35 (d, J = 8.1 Hz, 1H), 7.23 (t, J = 8.2 Hz, 1H), 7.20 – 7.16 (m, 1H), 7.13 – 7.06 (m, 1H), 7.00 (d, J = 1.9 Hz, 1H), 6.21 (d, J = 6.9 Hz, 1H), 3.85 (s, 3H), 3.79 (s, 3H), 3.31 (q, J = 6.9 Hz, 2H), 3.22 (d, J = 8.2 Hz, 2H), 2.80 (t, J = 7.3 Hz, 2H), 1.95 – 1.89 (m, 2H). **^{13}C NMR** (126 MHz, $CDCl_3$) δ 167.7, 165.9, 164.1, 144.2, 136.5, 129.8, 127.4, 122.1, 121.6, 119.3, 118.9, 115.5, 111.3, 52.8, 52.7, 39.8, 38.2, 29.7, 22.6. **HRMS (ESI)**: *m/z* calculated for $C_{19}H_{23}N_2O_5^+$ [M+H]$^+$ 359.1601, found 359.1616.

dimethyl 2-(3-((2-(2-methyl-1H-indol-3-yl)ethyl)amino)-3-oxopropylidene)malonate (4j).

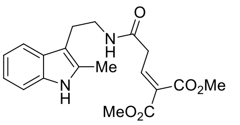

Prepared according to **GP-B**. Purification of the crude material was performed by silica gel column chromatography (50% EtOAc/*c*Hex) providing the title compound as a pale oil in 57% yield (204.3 mg, 0.56 mmol). **R_F** = 0.27 (50% EtOAc/*c*Hex). The product is present at the NMR as a mixture of two tautomers in a 1:0.3 ratio, the signal of the major one are reported. **^{1}H NMR** (600 MHz, $CDCl_3$) δ 8.08 (s, 1H), 7.47 (d, J = 7.7 Hz, 1H), 7.27 – 7.23 (m, 1H), 7.22 (t, J = 7.9 Hz, 1H), 7.13 – 7.08 (m, 1H), 7.08 – 7.01 (m, 1H), 6.07 (t, J = 6.0 Hz, 1H), 3.78 (s, 3H), 3.75 (s, 3H), 3.49 (q, J = 6.6 Hz, 2H), 3.20 (d, J = 7.9 Hz, 2H), 2.90 (t, J = 6.9 Hz, 2H), 2.34 (s, 3H). **^{13}C NMR** (151 MHz, $CDCl_3$) δ 167.8, 165.4, 164.1, 143.9, 135.4, 132.2, 129.6, 128.6, 121.2, 119.4, 117.8, 110.4, 108.2, 52.63, 52.58, 40.4, 37.7, 24.1, 11.6. **HRMS (ESI)**: *m/z* calculated for $C_{19}H_{23}N_2O_5^+$ [M+H]$^+$ 359.1601, found 359.1618.

dimethyl 2-(3-((2-(2-bromo-1H-indol-3-yl)ethyl)amino)-3-oxopropylidene)malonate (4k).

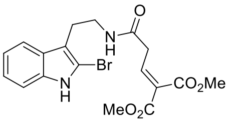

Prepared according to **GP-B**. Purification of the crude material was performed by silica gel column chromatography (40% EtOAc/*c*Hex) providing the title compound as a pail oil in 45% yield (190.5 mg, 0.45 mmol). **R_F** = 0.41 (50% EtOAc/*c*Hex). The product is present at the NMR as a mixture of two tautomers in a 1:0.25 ratio, the signal of the major one are reported. **^{1}H NMR** (600 MHz, $CDCl_3$) δ 8.32 (s, 1H), 7.58 – 7.54 (m, 1H), 7.35 – 7.29 (m, 1H), 7.25 (t, J = 8.0 Hz, 1H), 7.23 – 7.16 (m, 1H), 7.13 (m, 1H), 6.16 (s, 1H), 3.81 (s, 3H), 3.77 (s, 3H), 3.57 – 3.50 (m, 2H), 3.26 (d, J = 8.0 Hz, 2H), 2.97 (t, J = 6.7 Hz, 2H). **^{13}C NMR** (151 MHz, $CDCl_3$) δ 168.0, 165.5, 164.2, 144.0, 136.3, 129.7, 127.7, 122.6, 120.4, 118.1, 112.4, 110.7, 108.9, 52.7, 52.7, 39.7, 37.8, 24.8. **HRMS (ESI)**: *m/z* calculated for $C_{18}H_{20}BrN_2O_5^+$ [M+H]$^+$ 423.0550, found 423.0566.

dimethyl 2-(3-oxo-3-((2,3,4,9-tetrahydro-1H-carbazol-3-yl)amino)propylidene)malonate (4l).

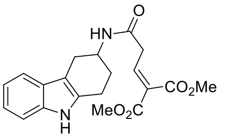

Prepared according to **GP-B**. Purification of the crude material was performed by silica gel column chromatography (50% EtOAc/*c*Hex) providing the title compound as a pale oil in 52% yield (192.6 mg, 0.52 mmol). **R_F** = 0.36 (66% EtOAc/*c*Hex). The product is present at the NMR as a mixture of two tautomers in a 1:0.8 ratio, the signal of the major one are reported. The minor tautomer is present as 1:1 mixture of rotamers. **^{1}H NMR** (500 MHz, $CDCl_3$) δ 7.91 (s, 1H), 7.42 (d, J = 7.9 Hz, 1H), 7.34 – 7.27 (m, 2H), 7.17 – 7.11 (m, 1H), 7.11 – 7.03 (m, 1H), 6.28 (d, J = 7.9 Hz, 1H), 4.44 – 4.33 (m, 1H), 3.79 (s, 3H), 3.77 (s, 3H), 3.27 (d, J = 7.9 Hz, 2H), 3.10 (dt, J = 9.9, 5.0 Hz, 1H), 2.89 – 2.74 (m, 2H), 2.71 – 2.55 (m, 1H), 2.13 – 2.05 (m, 1H), 2.02 – 1.94 (m, 1H). **^{13}C NMR** (126 MHz, $CDCl_3$) δ 167.5, 165.6, 164.1, 144.2, 136.3, 132.8, 129.6, 127.7, 121.6, 119.5, 117.8, 110.7, 107.3, 52.71, 52.70, 45.7, 38.1, 28.1, 27.7, 20.6. **HRMS (ESI)**: *m/z* calculated for $C_{20}H_{23}N_2O_5^+$ [M+H]$^+$ 371.1601, found 371.1610.

dimethyl 1,2,6a,7-tetrahydro-6H-pyrrolo[2,3-d]carbazole-6,6-dicarboxylate (7a).

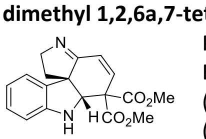

Prepared according to **GP-C**. Purification of the crude material by silica gel column chromatography (40% EtOAc/*c*Hex) providing the title compound as a yellow powder in 58% yield (37.9 mg, 0.116 mmol). **R_F** = 0.33 (50% EtOAc/*c*Hex). **^{1}H NMR** (500 MHz, $CDCl_3$) δ 7.02 (td, J = 7.6, 1.2 Hz, 1H), 6.88 (dd, J = 7.5, 1.2 Hz, 1H), 6.77 (dd, J = 10.1, 1.2 Hz, 1H), 6.69 (td, J = 7.5, 1.0 Hz, 1H), 6.64 (d, J = 10.0 Hz, 1H), 6.56 (d, J = 7.8 Hz, 1H), 4.93 (dd, J = 5.2, 1.4 Hz, 1H), 4.18 – 4.09 (m, 3H), 3.81 (s, 3H), 3.69 (s, 3H), 2.59 (dt, J = 12.2, 9.6 Hz, 1H), 2.40 (ddd, J = 12.1, 5.5, 2.4 Hz, 1H). **^{13}C NMR** (126 MHz, $CDCl_3$) δ 171.1, 168.8, 168.6, 148.7, 134.9, 133.4, 128.9, 128.1, 122.9, 120.1, 110.4, 67.4, 62.9, 61.9, 59.4, 53.4, 53.3, 41.7. **IR (neat):** νmax (cm^{-1}): 1728, 1242, 1213, 1205, 1188, 1087, 1027, 1010, 742, 623, 473. **HRMS (ESI)**: *m/z* calculated for $C_{18}H_{19}N_2O_4^+$ [M+H]$^+$ 327.1339, found 327.1341.

Prepared according to **GP-C** on 4.490 mmol scale. Purification of the crude material by silica gel column chromatography (40% EtOAc/*c*Hex) providing the title compound as a yellow powder in 54% yield (791.3 mg, 2.425 mmol).

dimethyl 10-methoxy-6a,7-dihydro-1H-pyrrolo[2,3-d]carbazole-6,6(2H)-dicarboxylate (7b).

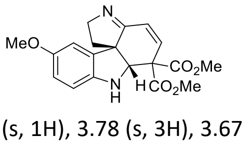

Prepared according to **GP-C**. Purification of the crude material by silica gel column chromatography (50% EtOAc/*c*Hex) providing the title compound as a yellow solid in 52% yield (37.1 mg, 0.104 mmol). $\mathbf{R_F}$ = 0.42 (66% EtOAc/*c*Hex). **^{1}H NMR** (500 MHz, $CDCl_3$) δ 6.77 (dd, J = 10.1, 1.3 Hz, 1H), 6.63 (d, J = 10.1 Hz, 1H), 6.59 (dd, J = 8.5, 2.5 Hz, 1H), 6.49 (d, J = 8.5 Hz, 1H), 6.45 (d, J = 2.6 Hz, 1H), 4.90 (s, 1H), 4.15 – 4.08 (m, 2H), 3.94 (s, 1H), 3.78 (s, 3H), 3.67 (s, 3H), 3.67 (s, 3H), 2.58 (dt, J = 12.0, 9.5 Hz, 1H), 2.43 – 2.38 (m, 1H). **^{13}C NMR** (126 MHz, $CDCl_3$) δ 171.1, 168.59, 168.56, 154.5, 142.5, 135.1, 134.7, 127.7, 114.2, 111.4, 108.9, 67.9, 63.3, 61.8, 59.2, 56.0, 53.3, 53.1, 41.3. **HRMS (ESI)**: *m/z* calculated for $C_{19}H_{21}N_2O_5^+$ $[M+H]^+$ 357.1445, found 357.1463.

dimethyl 10-chloro-6a,7-dihydro-1H-pyrrolo[2,3-d]carbazole-6,6(2H)-dicarboxylate (7c).

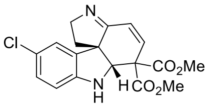

Prepared according to **GP-C**. Purification of the crude material by silica gel column chromatography (30% EtOAc/*c*Hex) providing the title compound as a yellow solid in 66% yield (47.6 mg, 0.132 mmol). $\mathbf{R_F}$ = 0.48 (50% EtOAc/*c*Hex). **^{1}H NMR** (500 MHz, $CDCl_3$) δ 6.95 (dd, J = 8.3, 2.1 Hz, 1H), 6.79 (d, J = 2.0 Hz, 1H), 6.77 (d, J = 10.1 Hz, 1H), 6.63 (d, J = 10.1 Hz, 1H), 6.44 (d, J = 8.3 Hz, 1H), 4.93 (d, J = 5.1 Hz, 1H), 4.17 – 4.08 (m, 3H), 3.79 (s, 3H), 3.67 (s, 3H), 2.58 (dt, J = 12.2, 9.6 Hz, 1H), 2.38 (dt, J = 12.2, 3.9 Hz, 1H). **^{13}C NMR** (126 MHz, $CDCl_3$) δ 170.4, 168.5, 168.3, 147.3, 135.0, 134.9, 128.7, 127.8, 124.4, 123.0, 111.0, 67.6, 62.7, 61.7, 59.1, 53.4, 53.3, 41.3. **HRMS (ESI)**: *m/z* calculated for $C_{18}H_{18}ClN_2O_4^+$ $[M+H]^+$ 361.0950, found 361.0962.

dimethyl 10-fluoro-6a,7-dihydro-1H-pyrrolo[2,3-d]carbazole-6,6(2H)-dicarboxylate (7d).

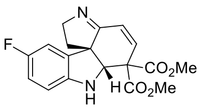

Prepared according to **GP-C**. Purification of the crude material by silica gel column chromatography (50% EtOAc/*c*Hex) providing the title compound as a yellow solid in 51% yield (35.1 mg, 0.102 mmol). $\mathbf{R_F}$ = 0.31 (50% EtOAc/*c*Hex). **^{1}H NMR** (500 MHz, $CDCl_3$) δ 6.76 (d, J = 10.1 Hz, 1H), 6.70 (td, J = 8.8, 2.6 Hz, 1H), 6.62 (d, J = 10.1 Hz, 1H), 6.56 (dd, J = 8.4, 2.5 Hz, 1H), 6.46 (dd, J = 8.5, 4.3 Hz, 1H), 4.93 (d, J = 6.2 Hz, 1H), 4.16 – 4.08 (m, 2H), 4.02 (d, J = 5.6 Hz, 1H), 3.79 (s, 3H), 3.67 (s, 3H), 2.61 – 2.55 (m, 1H), 2.40 – 2.35 (m, 1H). **^{13}C NMR** (126 MHz, $CDCl_3$) δ 170.6, 168.6, 168.4, 157.6 (d, J = 237.2 Hz), 144.6 (d, J = 1.4 Hz), 135.1, 134.8, 127.8, 115.2 (d, J = 23.4 Hz), 110.9 (d, J = 8.2 Hz), 110.1 (d, J = 24.2 Hz), 67.93, 63.06, 61.77, 59.17, 53.42, 53.24, 41.37. **HRMS (ESI)**: *m/z* calculated for $C_{18}H_{18}FN_2O_4^+$ $[M+H]^+$ 345.1245, found 345.1252.

diethyl 6a,7-dihydro-1H-pyrrolo[2,3-d]carbazole-6,6(2H)-dicarboxylate (7e).

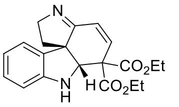

Prepared according to **GP-C**. Purification of the crude material by silica gel column chromatography (30% EtOAc/*c*Hex) providing the title compound as a pale oil in 58% yield (41.1 mg, 0.116 mmol). $\mathbf{R_F}$ = 0.68 (50% EtOAc/*c*Hex). **^{1}H NMR** (500 MHz, $CDCl_3$) δ 7.03 (td, J = 7.6, 1.2 Hz, 1H), 6.89 (dd, J = 7.6, 1.2 Hz, 1H), 6.81 (d, J = 9.9 Hz, 1H), 6.70 – 6.64 (m, 2H), 6.56 (d, J = 7.8 Hz, 1H), 4.95 (dd, J = 5.1, 1.4 Hz, 1H), 4.33 – 4.26 (m, 2H), 4.21 – 4.10 (m, 5H), 2.62 (dt, J = 12.1, 9.5 Hz, 1H), 2.42 (ddd, J = 12.0, 6.0, 1.9 Hz, 1H), 1.33 (t, J = 7.1 Hz, 3H), 1.23 (t, J = 7.1 Hz, 3H). **^{13}C NMR** (126 MHz, $CDCl_3$) δ 171.2, 168.1, 168.0, 148.6, 135.3, 133.1, 128.7, 127.6, 122.8, 119.8, 110.1, 67.0, 62.7, 62.3, 62.1, 62.0, 59.0, 41.4, 14.2, 13.9. **HRMS (ESI)**: *m/z* calculated for $C_{20}H_{23}N_2O_4^+$ $[M+H]^+$ 355.1652, found 355.1667.

dimethyl 7-methyl-6a,7-dihydro-1H-pyrrolo[2,3-d]carbazole-6,6(2H)-dicarboxylate (7f).

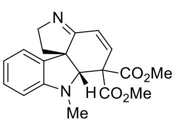

Prepared according to **GP-C**. Purification of the crude material by silica gel column chromatography (50% EtOAc/*c*Hex) providing the title compound as a yellow solid in 65% yield (44.2 mg, 0.130 mmol). $\mathbf{R_F}$ = 0.30 (50% EtOAc/*c*Hex). **^{1}H NMR** (500 MHz, $CDCl_3$) δ 7.08 (td, J = 7.7, 1.3 Hz, 1H), 6.78 (dd, J = 7.5, 1.3 Hz, 1H), 6.70 (d, J = 10.0 Hz, 1H), 6.67 (td, J = 7.4, 1.0 Hz, 1H), 6.57 (d, J = 10.1 Hz, 1H), 6.50 (d, J = 7.9 Hz, 1H), 4.55 (d, J = 1.3 Hz, 1H), 4.14 – 4.08 (m, 2H), 3.80 (s, 3H), 3.67 (s, 3H), 2.84 (s, 3H), 2.59 (dt, J = 12.2, 9.5 Hz, 1H), 2.37 (dt, J = 12.1, 4.2 Hz, 1H). **^{13}C NMR** (126 MHz, $CDCl_3$) δ 171.1, 168.8, 168.4, 152.3, 136.0, 133.5, 129.0, 127.2, 122.3, 119.9, 110.4, 76.3, 63.2, 61.0, 58.9, 53.3, 52.8, 41.4, 40.5. **HRMS (ESI)**: *m/z* calculated for $C_{19}H_{21}N_2O_4^+$ $[M+H]^+$ 341.1496, found 341.1507.

dimethyl 2-(acetoxymethyl)-6a,7-dihydro-1H-pyrrolo[2,3-d]carbazole-6,6(2H)-dicarboxylate (7g).

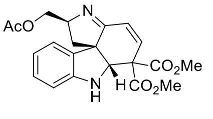

Prepared according to **GP-C**. Purification of the crude material by silica gel column chromatography (50% EtOAc/*c*Hex) providing the title compound as a yellow solid in 54% yield (43.0 mg, 0.108 mmol). $\mathbf{R_F}$ = 0.25 (50% EtOAc/*c*Hex). **^{1}H NMR** (500 MHz, $CDCl_3$) δ 7.02 (td, J = 7.6, 1.2 Hz, 1H), 6.89 (d, J = 7.4 Hz, 1H), 6.79 (dt, J = 10.1, 1.1 Hz, 1H), 6.68 (td, J = 7.5, 1.0 Hz, 1H), 6.62 (dd, J = 10.1, 1.2 Hz, 1H), 6.54 (d, J = 7.8 Hz, 1H), 4.91 (dd, J = 5.4, 1.1 Hz, 1H), 4.69 – 4.60 (m, 1H), 4.39 (dd, J = 11.2, 4.5 Hz, 1H), 4.17 (dd, J = 11.2, 7.6 Hz, 1H), 4.08 (d, J = 5.4 Hz, 1H), 3.81 (s, 3H), 3.66 (s, 3H), 2.49 (dd, J = 12.2, 6.3 Hz, 1H), 2.30 (dd, J = 12.2, 9.6 Hz, 1H), 2.14 (s, 3H). **^{13}C NMR** (126 MHz, $CDCl_3$) δ 171.5, 171.4, 168.5, 168.1, 148.8, 135.3, 132.7, 129.0, 127.9, 122.9, 120.1, 110.4, 69.4, 67.4, 67.1, 63.4, 61.7, 53.33, 53.26, 44.1, 21.1. **HRMS (ESI)**: *m/z* calculated for $C_{21}H_{23}N_2O_6^+$ $[M+H]^+$ 399.1551, found 399.1559.

dimethyl 5H-indolo[2,3-d]carbazole-6,6(5aH)-dicarboxylate (7h).

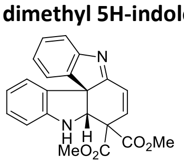

Prepared according to **GP-C**. Purification of the crude material by silica gel column chromatography (20% EtOAc/*c*Hex) providing the title compound as a red oil in 26% yield (19.5 mg, 0.052 mmol). $\mathbf{R_F}$ = 0.56 (50% EtOAc/*c*Hex). **^{1}H NMR** (500 MHz, $CDCl_3$) δ 7.66 (d, J = 7.7 Hz, 1H), 7.36 (td, J = 7.5, 1.4 Hz, 1H), 7.27 – 7.23 (m, 1H), 7.20 (td, J = 7.4, 1.0 Hz, 1H), 7.00 (td, J = 7.6, 1.3 Hz, 1H), 6.89 (s, 2H), 6.66 (d, J = 7.8 Hz, 1H), 6.53 (td, J = 7.5, 1.0 Hz, 1H), 6.23 (dd, J = 7.6, 1.2 Hz, 1H), 5.24 (d, J = 5.4 Hz, 1H), 4.35 (d, J = 5.5 Hz, 1H), 3.89 (s, 3H), 3.47 (s, 3H). **^{13}C NMR** (126 MHz, $CDCl_3$) δ 177.9, 168.7, 167.8, 154.9, 150.1, 145.7, 136.1, 128.9, 128.6, 128.5, 128.0, 126.8, 122.9, 122.1, 121.2, 120.1, 110.9, 67.4, 61.9, 60.9, 53.38, 53.35. **HRMS (ESI)**: *m/z* calculated for $C_{22}H_{19}N_2O_4^+$ $[M+H]^+$ 375.1339, found 375.1355.

dimethyl 2,3,7a,8-tetrahydropyrido[2,3-d]carbazole-7,7(1H)-dicarboxylate (7i).

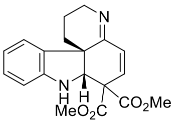

Prepared according to **GP-C**. Purification of the crude material by silica gel column chromatography (50% EtOAc/*c*Hex) providing the title compound as a yellow solid in 55% yield (37.4 mg, 0.110 mmol). $\mathbf{R_F}$ = 0.26 (66% EtOAc/*c*Hex). **^{1}H NMR** (500 MHz, $CDCl_3$) δ 7.00 (td, J = 7.6, 1.3 Hz, 1H), 6.93 (dd, J = 7.6, 1.2 Hz, 1H), 6.67 (td, J = 7.5, 1.0 Hz, 1H), 6.59 – 6.51 (m, 2H), 6.27 (d, J = 10.0 Hz, 1H), 4.56 (dd, J = 4.3, 1.3 Hz, 1H), 4.26 (d, J = 4.3 Hz, 1H), 4.09 (dd, J = 19.1, 5.5 Hz, 1H), 3.79 – 3.72 (m, 1H), 3.69 (s, 3H), 3.67 (s, 3H), 2.20 – 2.12 (m, 2H), 2.05 – 1.98 (m, 1H), 1.76 – 1.68 (m, 1H). **^{13}C NMR** (126 MHz, $CDCl_3$) δ 169.1, 168.8, 163.4, 149.1, 133.7, 133.6, 130.2, 128.5, 124.8, 119.0, 110.3, 67.7, 60.4, 53.2, 53.0, 49.1, 48.9, 33.5, 18.8. **HRMS (ESI)**: *m/z* calculated for $C_{19}H_{21}N_2O_4^+$ [M+H]$^+$ 341.1496, found 341.1504.

methyl 2-methyl-5'-oxo-3',5'-dihydro-2'H-spiro[indole-3,1'-indolizine]-6'-carboxylate (15j).

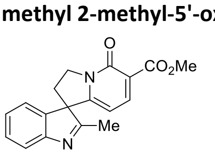

Prepared according to **GP-C**. Purification of the crude material by silica gel column chromatography (5% MeOH/DCM) providing the title compound as a white solid in 89% yield (54.9 mg, 0.178 mmol). $\mathbf{R_F}$ = 0.62 (10% MeOH/DCM). **^{1}H NMR** (600 MHz, $CDCl_3$) δ 8.00 (d, J = 7.5 Hz, 1H), 7.53 (d, J = 7.8 Hz, 1H), 7.37 – 7.33 (m, 1H), 7.20 – 7.17 (m, 2H), 5.50 (d, J = 7.4 Hz, 1H), 4.56 (ddd, J = 13.9, 8.4, 5.7 Hz, 1H), 4.47 – 4.42 (m, 1H), 3.81 (s, 3H), 2.57 – 2.51 (m, 2H), 2.20 (s, 3H). **^{13}C NMR** (151 MHz, $CDCl_3$) δ 179.8, 165.4, 158.8, 154.7, 154.6, 146.1, 140.9, 129.6, 126.7, 122.1, 120.6, 118.9, 99.3, 68.5, 52.2, 48.5, 30.1, 16.4. **HRMS (ESI)**: *m/z* calculated for $C_{18}H_{17}N_2O_3^+$ [M+H]$^+$ 309.1234, found 309.1251.

methyl 2-bromo-5'-oxo-3',5'-dihydro-2'H-spiro[indole-3,1'-indolizine]-6'-carboxylate (15k)

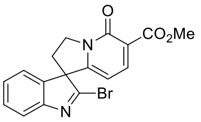

Prepared according to **GP-C**. Purification of the crude material by silica gel column chromatography (5% MeOH/DCM) providing the title compound as an orange solid in 24% yield (17.9 mg, 0.048 mmol). $\mathbf{R_F}$ = 0.61 (10% MeOH/DCM). **^{1}H NMR** (500 MHz, $CDCl_3$) δ 8.10 (d, J = 7.5 Hz, 1H), 7.31 (td, J = 7.7, 1.5 Hz, 1H), 7.12 – 7.05 (m, 3H), 5.77 (d, J = 7.5 Hz, 1H), 4.58 – 4.52 (m, 2H), 3.86 (s, 3H), 2.76 (ddd, J = 13.1, 6.4, 4.6 Hz, 1H), 2.57 – 2.51 (m, 1H). **^{13}C NMR** (126 MHz, $CDCl_3$) δ 177.1, 165.7, 159.0, 155.4, 146.2, 141.7, 130.1, 129.4, 123.9, 123.6, 118.9, 111.1, 100.4, 60.2, 52.4, 48.6, 33.2. **HRMS (ESI)**: *m/z* calculated for $C_{17}H_{14}BrN_2O_3^+$ [M+H]$^+$ 373.0182, found 373.0192.

methyl 4-oxo-4,6,7,8-tetrahydro-6,13b-methanopyrido[1',2':1,2]azepino[4,3-b]indole-3-carboxylate (15l).

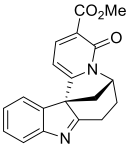

Prepared according to **GP-C**. Purification of the crude material by silica gel column chromatography (5% MeOH/DCM) providing the title compound as a yellow solid in 59% yield (37.8 mg, 0.118 mmol). $\mathbf{R_F}$ = 0.11 (EtOAc). **^{1}H NMR** (500 MHz, $CDCl_3$) δ 8.08 (dd, J = 7.3, 1.6 Hz, 1H), 7.65 (d, J = 7.9 Hz, 1H), 7.48 (tt, J = 7.6, 1.4 Hz, 1H), 7.43 (d, J = 7.4 Hz, 1H), 7.32 (tt, J = 7.5, 1.2 Hz, 1H), 5.70 (dd, J = 7.3, 1.2 Hz, 1H), 5.34 – 5.26 (m, 1H), 3.89 (s, 3H), 3.05 – 2.98 (m, 2H), 2.71 (ddt, J = 11.8, 6.4, 2.3 Hz, 1H), 2.54 (dddd, J = 17.3, 10.9, 9.1, 1.5 Hz, 1H), 2.08 – 2.00 (m, 1H), 1.98 (dd, J = 11.6, 1.1 Hz, 1H). **^{13}C NMR** (126 MHz, $CDCl_3$) δ 179.50, 165.67, 158.34, 157.24, 154.35, 146.11, 133.93, 130.10, 126.36, 123.38, 121.26, 120.12, 98.46, 66.01, 56.18, 52.51, 42.97, 26.20, 24.88. **HRMS (ESI)**: *m/z* calculated for $C_{19}H_{17}N_2O_3^+$ [M+H]$^+$ 321.1234, found 321.1248.

dimethyl-5-(phenylthio)-1,2,4,5,6a,7-hexahydro-6H-pyrrolo[2,3-d]carbazole-6,6-dicarboxylate (16)

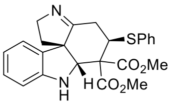

To a solution of **7a** (411.5 mg, 1.261 mmol, 1.0 eq) in DCM (6.3 mL, 0.2 M) at 0 °C, TEA (351 µL, 2.522 mmol, 2.0 eq.) and tiophenol (257 µL, 2.522 mmol, 2.0 eq.) were added. The mixture was stirred at room temperature for 2 hours. Then, it was diluted with DCM (20 mL) and water (20 mL), the phases were separted and the aqueous layer was extracted with DCM (2x20 mL). The combined organic layers were dried over Na_2SO_4, filtered and the solvent was removed under reduced pressure. Purification of the crude material was performed by silica gel column chromatography (40% EtOAc/*c*Hex) providing the title compound as a yellow foam in 58% yield (297.3 mg, 0.681 mmol). $\mathbf{R_F}$ = 0.36 (50% EtOAc/*c*Hex). **^{1}H NMR** (500 MHz, $CDCl_3$) δ 7.37 – 7.33 (m, 2H), 7.26 – 7.18 (m, 3H), 6.98 (td, J = 7.6, 1.4 Hz, 1H), 6.66 (dd, J = 7.3, 1.3 Hz, 1H), 6.60 (td, J = 7.4, 1.0 Hz, 1H), 6.55 (d, J = 7.8 Hz, 1H), 4.65 (s, 1H), 4.45 (s, 1H), 3.99 – 3.92 (m, 2H), 3.72 – 3.65 (m, 1H), 3.63 (s, 3H), 3.60 – 3.55 (m, 1H), 3.29 (s, 3H), 2.86 (dd, J = 15.8, 4.1 Hz, 1H), 2.22 (ddd, J = 12.3, 10.2, 8.5 Hz, 1H), 2.06 (dd, J = 12.2, 6.4 Hz, 1H). **^{13}C NMR** (126 MHz, $CDCl_3$) δ 170.1, 169.9, 148.2, 135.90, 135.89, 132.0 (2C), 129.1 (2C), 127.9, 127.5, 121.3, 119.2, 109.4, 66.5, 61.0, 60.5, 54.3, 53.1, 52.5, 47.4, 44.3, 37.1, 32.9. **IR (neat):** vmax (cm^{-1}): 1994, 1728, 1605, 1467, 1433, 1242, 1172, 1087, 1037, 1016, 1001, 743, 730, 688, 651. **HRMS (ESI)**: *m/z* calculated for $C_{24}H_{25}N_2O_4S^+$ [M+H]$^+$ 437.1530, found 437.1515.

dimethyl-5-(phenylthio)-1,2,3,3a,4,5,6a,7-octahydro-6H-pyrrolo[2,3-d]carbazole-6,6-dicarboxylate (17)

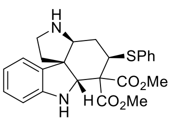

To a solution of **14** (297.3 mg, 0.681 mmol, 1.0 eq.) in MeOH:AcOH (4:1, 6.8 mL, 0.1 M) at 0 °C, three portion of $NaBH_4$ (each of 128.8 mg, 3.405 mmol, 5.0 eq., every 30 min) were added. The mixture was basified to pH = 10 with a saturated solution of Na_2CO_3 and extracted with EtOAc (4x20 mL). The combined organic layers were dried over Na_2SO_4, filtered and the solvent was removed under reduced pressure. Purification of the crude material was performed by silica gel column chromatography (50% EtOAc/*c*Hex, 1% TEA) providing the title compound as a pale foam in 86% yield (257.0 mg, 0.586 mmol). $\mathbf{R_F}$ = 0.11 (EtOAc). **^{1}H NMR** (500 MHz, $CDCl_3$) δ 7.34 – 7.30 (m, 2H), 7.18 – 7.14 (m, 2H), 7.13 – 7.09 (m, 1H), 6.91 (dd, J = 7.6, 1.3 Hz, 1H), 6.80 (dd, J = 7.4, 1.2 Hz, 1H), 6.58 (td, J = 7.4, 1.0 Hz, 1H), 6.49 (d, J = 7.5 Hz, 1H), 4.42 (d, J = 1.7 Hz, 1H), 4.38 (d, J = 1.7 Hz, 1H), 3.82 – 3.78 (m, 1H), 3.54 (s, 3H), 3.34 (s, 3H), 3.20 (dd, J = 8.5, 5.8 Hz, 1H), 3.06 – 2.98 (m, 2H), 2.44 – 2.39 (m, 1H), 2.13 – 2.08 (m, 1H), 1.97 – 1.92 (m, 1H), 1.85 – 1.80 (m, 1H). **^{13}C NMR** (126 MHz, $CDCl_3$) δ 170.0, 169.9, 148.2, 136.6, 135.8, 131.9 (2C), 129.1 (2C), 127.9, 127.5, 121.3, 119.2, 109.4, 66.5, 61.0, 60.4, 54.3, 53.1, 52.4, 47.4, 44.2, 37.2, 32.9. **IR (neat):** vmax (cm^{-1}): 2033, 1996, 1726, 1209, 690, 609, 582, 563, 453. **HRMS (ESI)**: *m/z* calculated for $C_{24}H_{27}N_2O_4S^+$ [M+H]$^+$ 439.1686, 439.1697.

dimethyl-3-((Z)-2-iodobut-2-en-1-yl)-5-(phenylthio)-1,2,3,3a,4,5,6a,7-octahydro-6H-pyrrolo[2,3-d]carbazole-6,6-dicarboxylate (19)

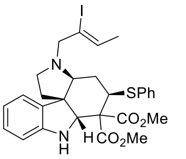

To a solution of **15** (257.0 mg, 0.586 mmol, 1.00 eq.), K_2CO_3 (89.1 mg, 0.645 mmol, 1.10 eq.), TBAI (10.8 mg, 0.029 mmol, 0.05 eq.) in acetonitrile (11.7 mL, 0.05M), **16**[1] (194.1 mg, 0.703 mmol, 1.2 eq.) was added and the mixture was stirred overnight. Then, it was diluted with EtOAc (20 mL) and water (20 mL), the phases were separted and the aqueous layer was extracted with EtOAc (3x20 mL). The combined organic layers were dried over Na_2SO_4, filtered and the solvent was removed under reduced pressure. Purification of the crude material was performed by silica gel column chromatography (10% EtOAc/*c*Hex) providing the title compound as a white foam in 65% yield (235.7 mg, 0.381 mmol). $\mathbf{R_F}$ = 0.69 (20 EtOAc/*c*Hex). **^{1}H NMR** (600 MHz, $CDCl_3$) δ 7.42 (d, J = 7.0 Hz, 2H), 7.29 – 7.24 (m, 2H), 7.20 (t, J = 7.3 Hz, 1H), 7.15 (dd, J = 7.4, 1.2 Hz, 1H), 7.02 (td, J = 7.6, 1.3 Hz, 1H), 6.70 (td, J = 7.4, 1.0 Hz, 1H), 6.58 (d, J = 7.7 Hz, 1H), 5.84 (q, J = 6.4 Hz, 1H), 4.45 (s, 2H), 3.93 (dd, J = 9.8, 5.3 Hz, 1H), 3.58 – 3.50 (m, 4H), 3.45 (d, J = 14.1 Hz, 1H), 3.40 (s, 3H), 3.06 (dd, J = 9.4, 5.3 Hz, 1H), 2.83 (dd, J = 8.2, 6.2 Hz, 2H), 2.42 (dt, J = 14.2, 5.3 Hz, 1H), 2.21 (dt, J = 13.1, 7.5 Hz, 1H), 2.03 – 1.91 (m, 2H), 1.74 (d, J = 6.4 Hz, 3H). **^{13}C NMR** (151 MHz, $CDCl_3$) δ 170.5, 169.8, 148.1, 136.7, 136.3, 131.4 (2C), 131.2, 129.0 (2C), 127.8, 127.1, 123.3, 119.0, 109.3, 109.1, 67.2, 64.1, 63.8, 61.4, 53.5, 53.0, 52.2, 48.9, 46.1, 36.1, 30.6, 21.8. **IR (neat):** vmax (cm^{-1}): 1724, 1479, 1463, 1433, 1245, 1209, 1173, 1142, 1121, 1101, 1087, 1070, 1022, 739, 690, 660. **HRMS (ESI)**: *m/z* calculated for $C_{28}H_{32}IN_2O_4S^+$ $[M+H]^+$ 619.1122, found 619.1118.

dimethyl-3-((Z)-2-iodobut-2-en-1-yl)-1,2,3,3a,6a,7-hexahydro-6H-pyrrolo[2,3-d]carbazole-6,6-dicarboxylate (20)

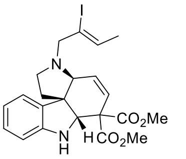

To a solution of **17** (235.7 mg, 0.381 mmol, 1.0 eq.) in DCM (3.8 mL, 0.10 M) cooled at 0 °C, was added 75% *m*-CPBA (105.2 mg, 0.457 mmol, 1.2 eq.) and stirred for 20 min. The solvent was then removed, the crude was redesolved in toluene (7.6 mL, 0.05 M) and refluxed for 2 hours. The mixture was cooled to room temperature, EtOAc (30 mL) was added and extracted with 1M NaOH (20 mL). Finally, the organic layer wes dried over Na_2SO_4, filtered and the solvent was removed under reduced pressure. Purification of the crude material was performed by silica gel column chromatography (20% EtOAc/*c*Hex) providing the title compound as a yellow foam in 81% yield (157.1 mg, 0.309 mmol). $\mathbf{R_F}$ = 0.25 (15% EtOAc/*c*Hex). **^{1}H NMR** (500 MHz, $CDCl_3$) δ 7.03 – 6.97 (m, 2H), 6.70 (t, J = 7.4 Hz, 1H), 6.57 (d, J = 7.7 Hz, 1H), 6.23 (dd, J = 10.1, 2.6 Hz, 1H), 6.02 (dd, J = 10.0, 2.0 Hz, 1H), 5.95 (q, J = 6.8 Hz, 1H), 4.58 (s, 1H), 4.20 (s, 1H), 3.80 (s, 3H), 3.76 (d, J = 14.1 Hz, 1H), 3.50 (d, J = 13.7 Hz, 1H), 3.42 (s, 1H), 3.19 (s, 3H), 3.05 – 2.96 (m, 1H), 2.66 – 2.57 (m, 1H), 2.20 – 2.13 (m, 1H), 2.02 (ddd, J = 12.3, 5.9, 1.9 Hz, 1H), 1.81 (d, J = 6.3 Hz, 3H). **^{13}C NMR** (126 MHz, $CDCl_3$) δ 170.8, 168.6, 148.1, 137.0, 134.7, 132.1, 127.9, 122.9, 122.3, 119.0, 109.4, 108.4, 69.7, 66.6, 65.7, 59.7, 53.6, 53.1, 52.8, 51.0, 42.5, 21.9. **IR (neat):** vmax (cm^{-1}): 1728, 1433, 1248, 1230, 1211, 1180, 1157, 1121, 1095, 1068, 1051, 1020, 741, 729, 465, 449. **HRMS (ESI)**: *m/z* calculated for $C_{22}H_{26}IN_2O_4^+$ $[M+H]^+$ 509.0932, found 509.0945.

methyl-3-((Z)-2-iodobut-2-en-1-yl)-2,3,3a,4,6a,7-hexahydro-1H-pyrrolo[2,3-d]carbazole-6-carboxylate (21)

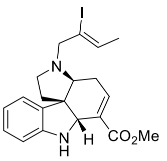

To a solution of **18** (157.1 mg, 0.309 mmol, 1.0 eq.) in THF (1.5 mL, 0.2 M) were added MeOH (200 µL), water (200 µL) and KOH (17.3 mg, 0.309 mmol, 1.0 eq.) at 0 °C and was stirred at this temperature for three hours. Then, it was diluted with EtOAc (10 mL) and water (10 mL), the phases were separted and the aqueous layer was extracted with EtOAc (2x10 mL). The combined organic layers were dried over Na_2SO_4, filtered and the solvent was removed under reduced pressure. Purification of the crude material was performed by silica gel column chromatography (20% EtOAc/*c*Hex) providing the title compound as a yellow foam in 74% yield (103.2 mg, 0.229 mmol). $\mathbf{R_F}$ = 0.39 (15% EtOAc/*c*Hex). **^{1}H NMR** (500 MHz, $CDCl_3$) δ 7.11 (dd, J = 7.4, 1.2 Hz, 1H), 7.04 – 7.00 (m, 2H), 6.71 (td, J = 7.4, 1.0 Hz, 1H), 6.57 (d, J = 7.7 Hz, 1H), 5.86 (q, J = 6.5 Hz, 1H), 4.57 (s, 1H), 4.32 (d, J = 1.7 Hz, 1H), 3.78 (s, 3H), 3.59 (d, J = 13.4 Hz, 1H), 3.31 (d, J = 14.3 Hz, 1H), 3.15 (t, J = 3.9 Hz, 1H), 3.13 – 3.07 (m, 1H), 2.69 (td, J = 9.7, 4.5 Hz, 1H), 2.38 (dt, J = 19.3, 4.3 Hz, 1H), 2.29 – 2.22 (m, 1H), 2.16 (ddd, J = 13.0, 8.5, 4.7 Hz, 1H), 1.97 (ddd, J = 12.8, 9.9, 6.5 Hz, 1H), 1.78 (d, J = 6.5 Hz, 3H). **^{13}C NMR** (126 MHz, $CDCl_3$) δ 167.5, 150.2, 139.4, 132.7, 130.8, 130.1, 128.2, 123.3, 118.6, 109.3, 109.1, 65.4, 62.6, 61.3, 53.6, 51.9, 50.5, 37.8, 25.3, 21.8. **IR (neat):** vmax (cm^{-1}): 1701, 1483, 1435, 1248, 1211, 1095, 908, 735, 644, 606, 594, 552, 536, 494. **HRMS (ESI)**: *m/z* calculated for $C_{20}H_{24}IN_2O_2^+$ $[M+H]^+$ 451.0877, found 451.0887. Data matching those reported in literature.[2]

Akuammicine (3)

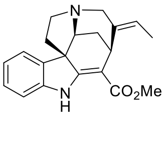

Prepared according to literature procedure from **19**. Purification of the crude material was performed by silica gel column chromatography (5% TEA/EtOAc) providing the title compound as a reddish solid in 91% yield (67.1 mg, 0.208 mmol). $\mathbf{R_F}$ = 0.08 (EtOAc). **^{1}H NMR** (500 MHz, $CDCl_3$) δ 8.99 (s, 1H), 7.22 (d, J = 7.0 Hz, 1H), 7.14 (td, J = 7.7, 1.2 Hz, 1H), 6.88 (td, J = 7.5, 1.0 Hz, 1H), 6.81 (d, J = 7.7 Hz, 1H), 5.35 (q, J = 6.8 Hz, 1H), 4.04 (dt, J = 3.6, 2.0 Hz, 1H), 3.95 – 3.92 (m, 1H), 3.89 (dq, J = 15.1, 2.0 Hz, 1H), 3.80 (s, 3H), 3.26 (dt, J = 12.6, 6.3 Hz, 1H), 3.02 (dd, J = 12.4, 6.6 Hz, 1H), 2.95 (d, J = 15.0 Hz, 1H), 2.53 – 2.49 (m, 1H), 2.41 (ddd, J = 13.4, 4.0, 2.2 Hz, 1H), 1.82 (dd, J = 12.4, 5.6 Hz, 1H), 1.60 (dt, J = 7.0, 1.7 Hz, 3H), 1.33 – 1.27 (m, 1H). **^{13}C NMR** (126 MHz, $CDCl_3$) δ 168.0, 167.9, 143.5, 139.0, 137.0, 127.9, 121.1 (2C), 120.9, 109.5, 101.4, 62.0, 57.6, 57.0, 56.4, 51.1, 46.3, 31.0, 29.8, 13.0. **IR (neat):** vmax (cm^{-1}): 3358, 1654, 1595, 1431, 1230, 1201, 1186, 1159, 1124, 1097, 1074, 1057, 1047, 746, 733, 696. **HRMS (ESI)**: *m/z* calculated for $C_{20}H_{23}N_2O_2^+$ $[M+H]^+$ 323.1754, found 323.1753. Data matching those reported in literature.[2]

NMR data

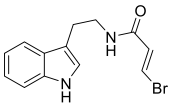

S1a

^{1}H-NMR
500 MHz
$CDCl_3$

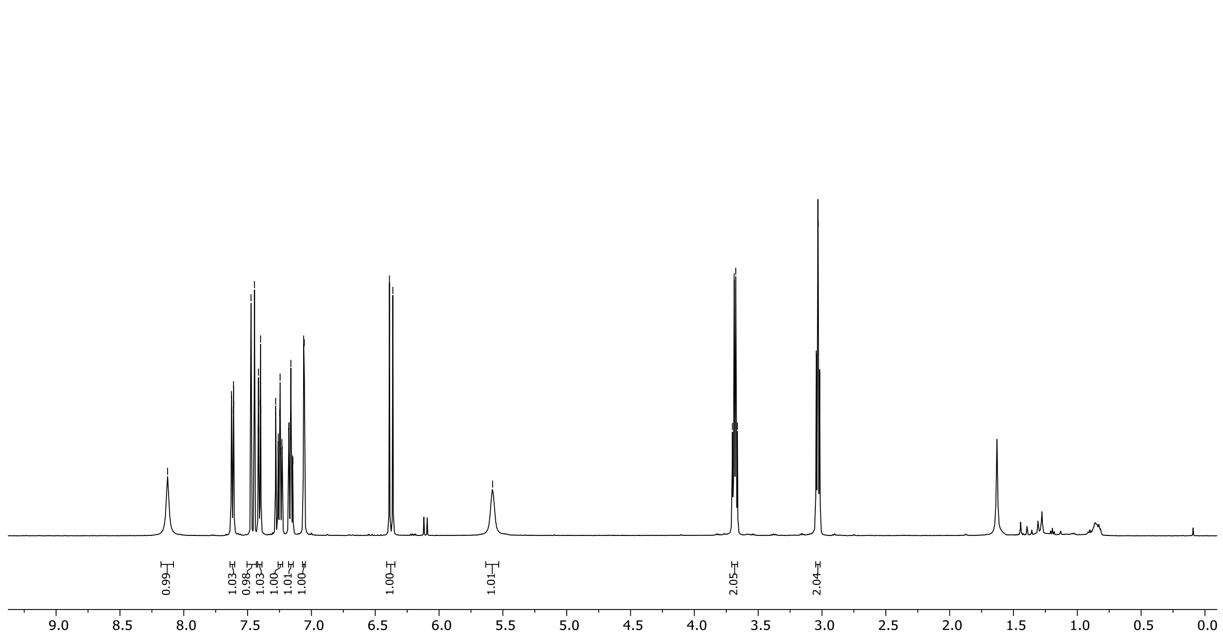

^{13}C-NMR
126 MHz
$CDCl_3$

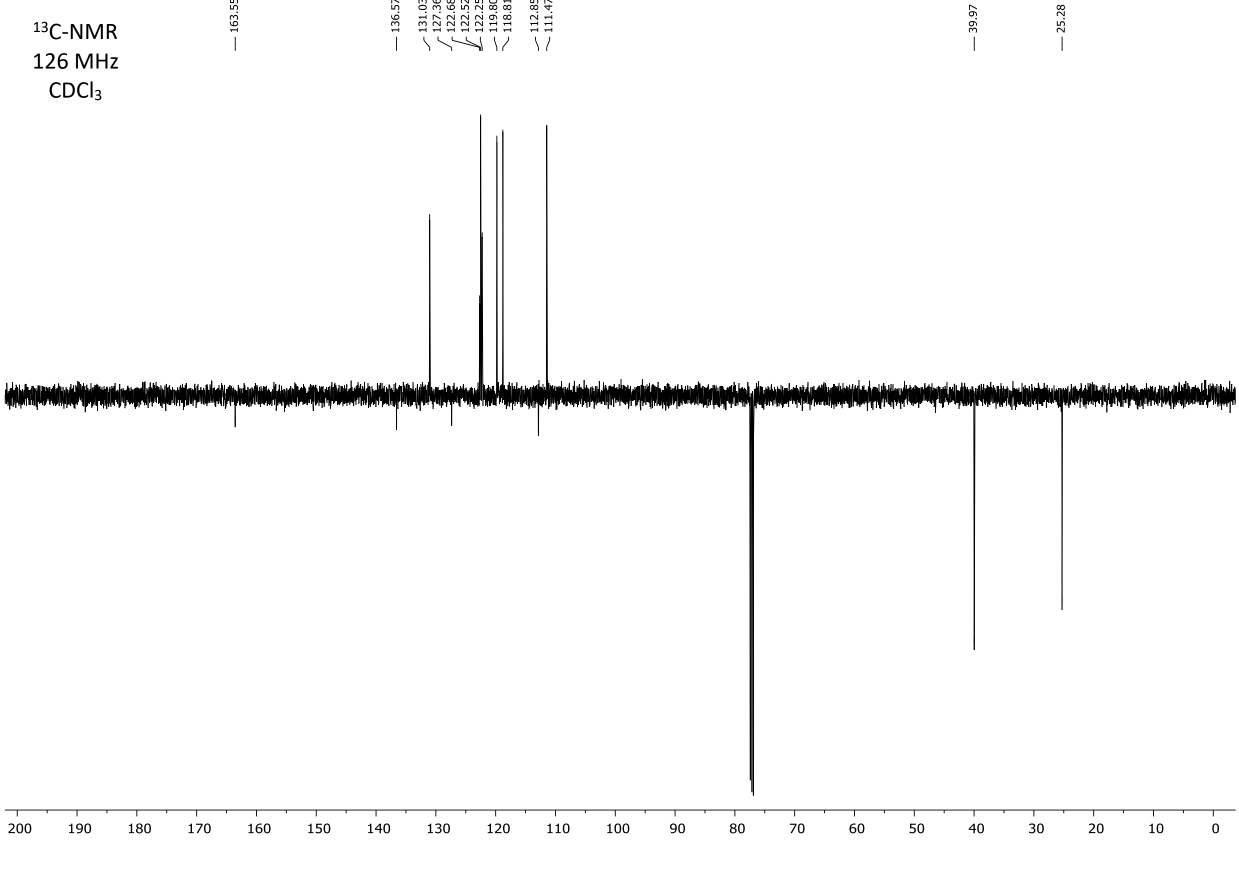

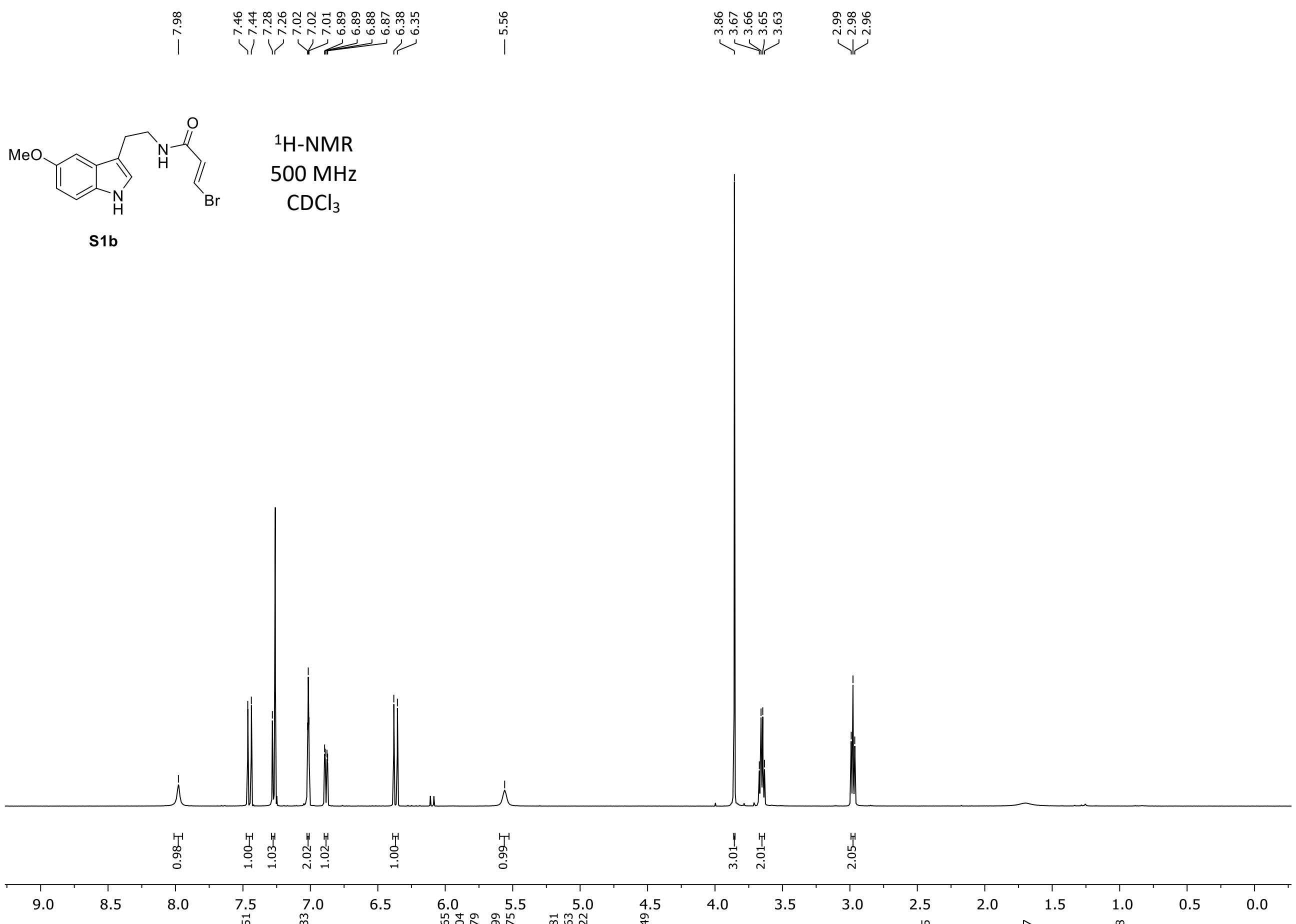

S1b
7.98
7.46 7.44 7.28 7.26 7.02 7.02 7.01 6.89 6.89 6.88 6.87 6.38 6.35
5.56
3.86 3.67 3.66 3.65 3.63
2.99 2.98 2.96
^{1}H-NMR
500 MHz
$CDCl_3$
0.98 1.00 1.03 2.02 1.02 1.00 0.99 3.01 2.01 2.05
9.0 8.5 8.0 7.5 7.0 6.5 6.0 5.5 5.0 4.5 4.0 3.5 3.0 2.5 2.0 1.5 1.0 0.5 0.0

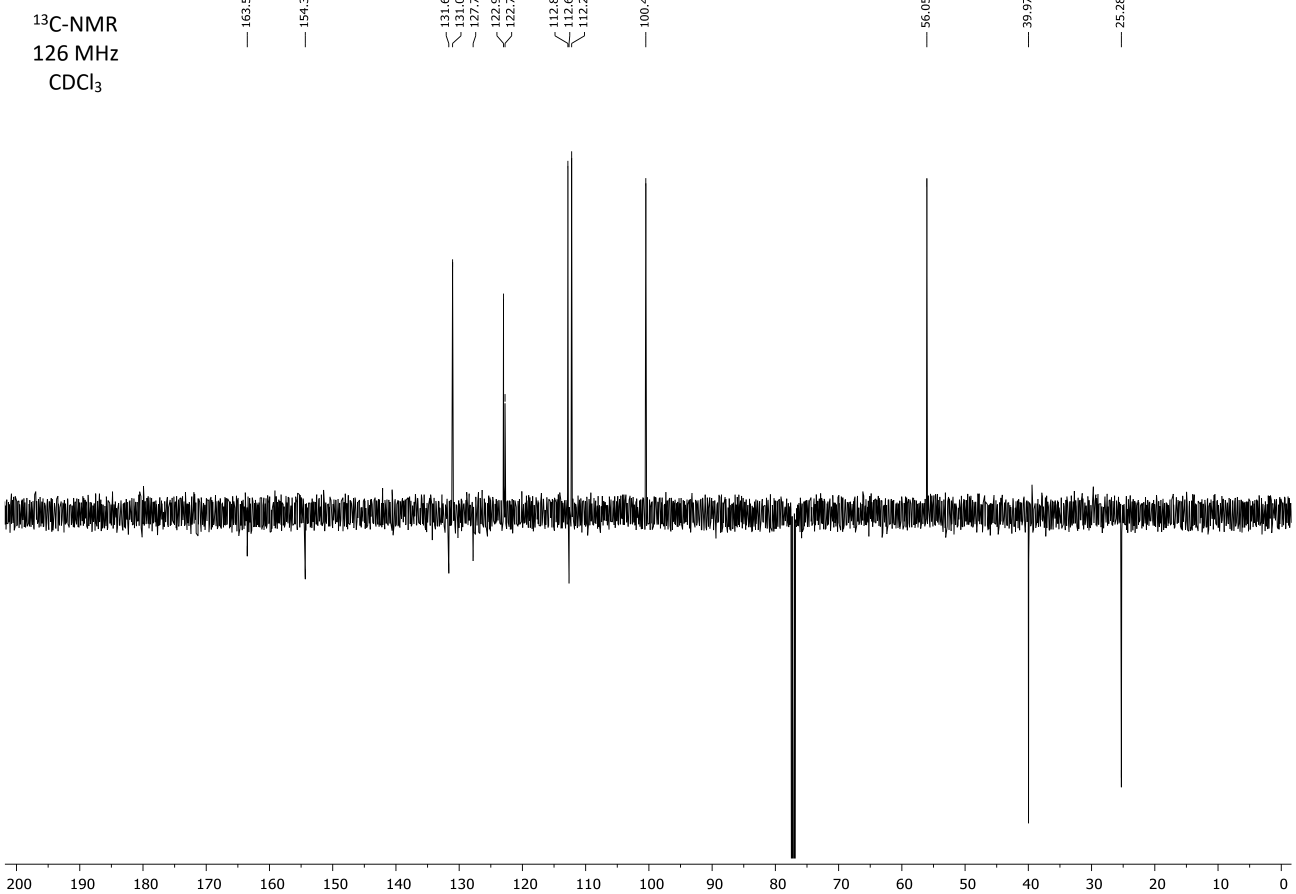

^{13}C-NMR
126 MHz
$CDCl_3$
163.51
154.33
131.65 131.04 127.79 122.99 122.75
112.81 112.63 112.22
100.49
56.05
39.97
25.28
200 190 180 170 160 150 140 130 120 110 100 90 80 70 60 50 40 30 20 10 0

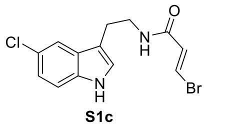

S1c

^{1}H-NMR
500 MHz
$CDCl_3$

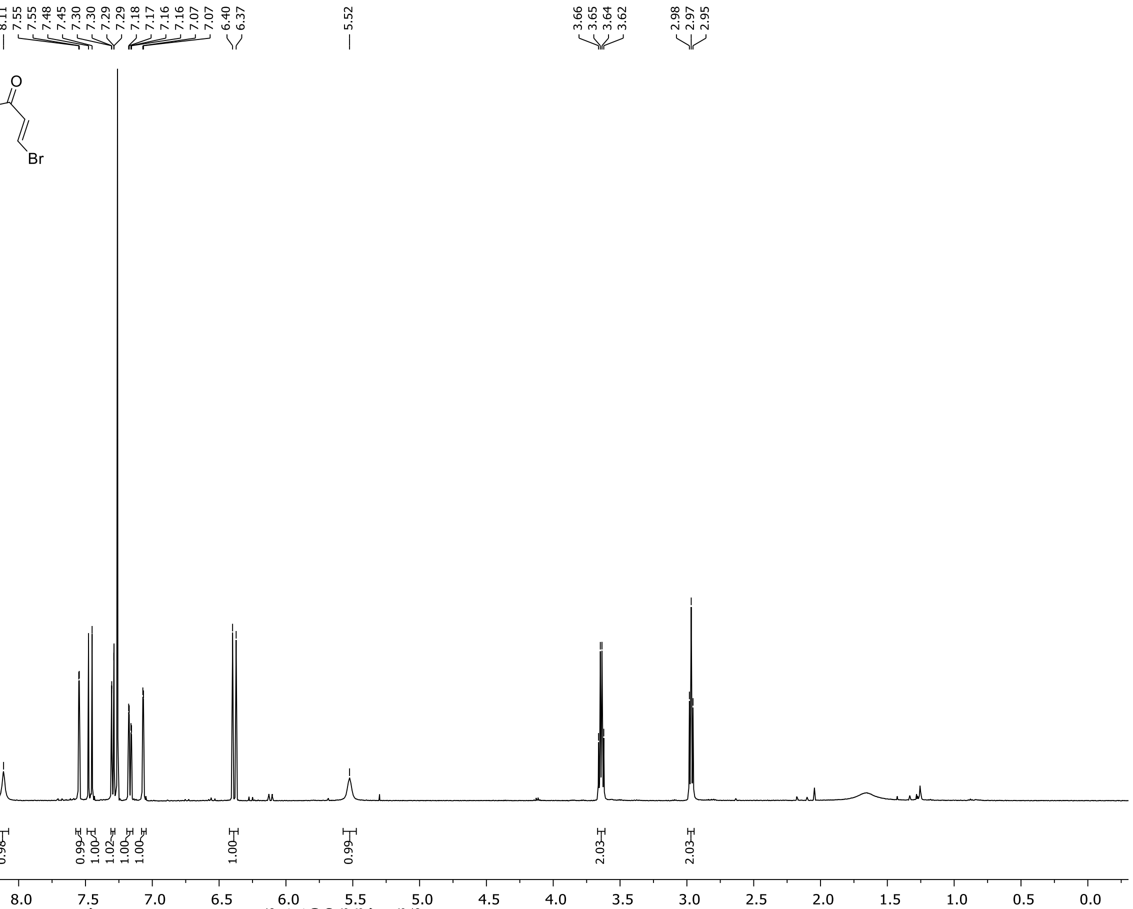

^{13}C-NMR
126 MHz
$CDCl_3$

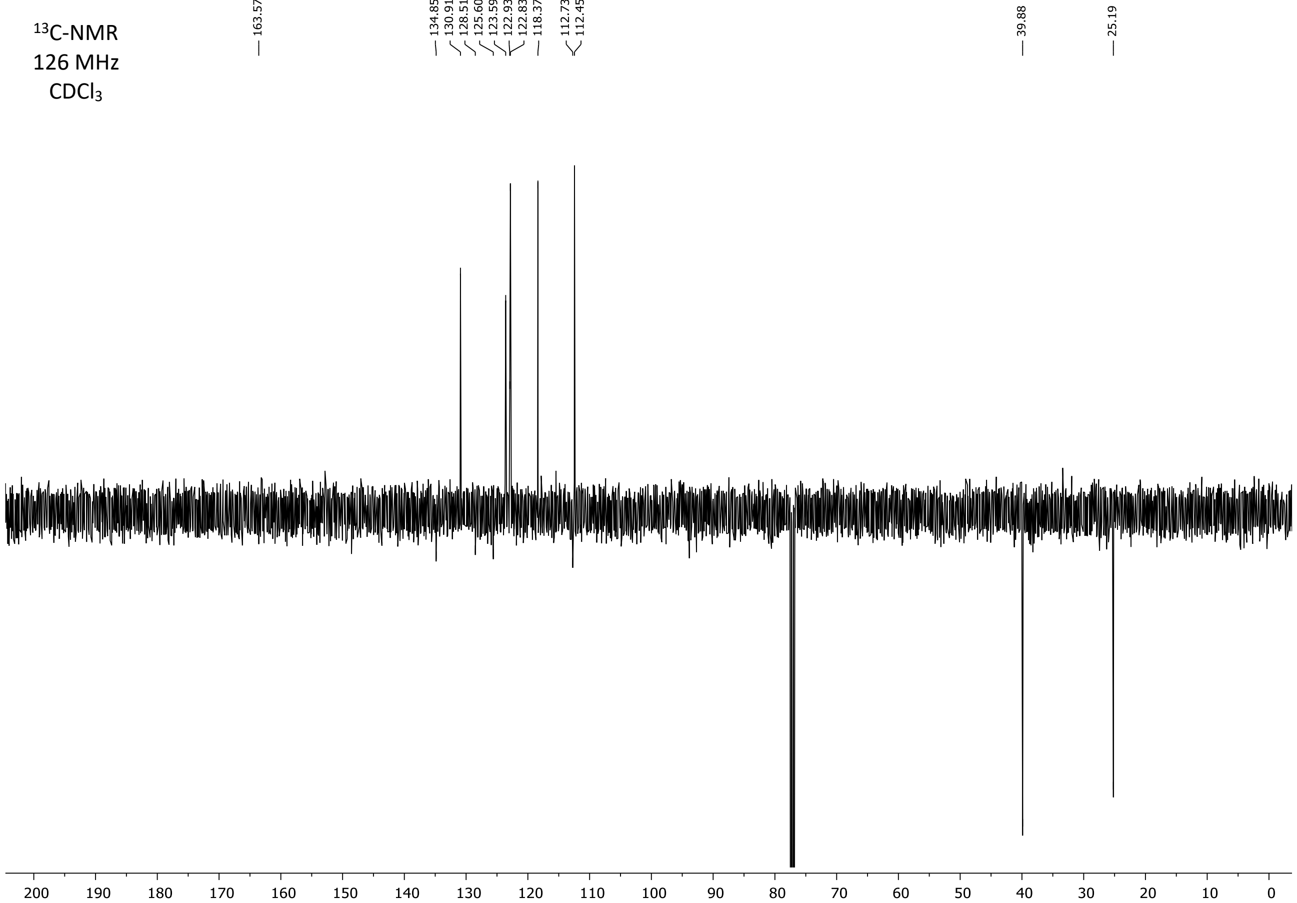

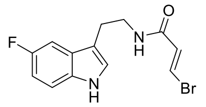
F
N
H
O
H
Br

S1d

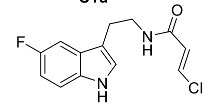
F
N
H
O
H
Cl

S1d'

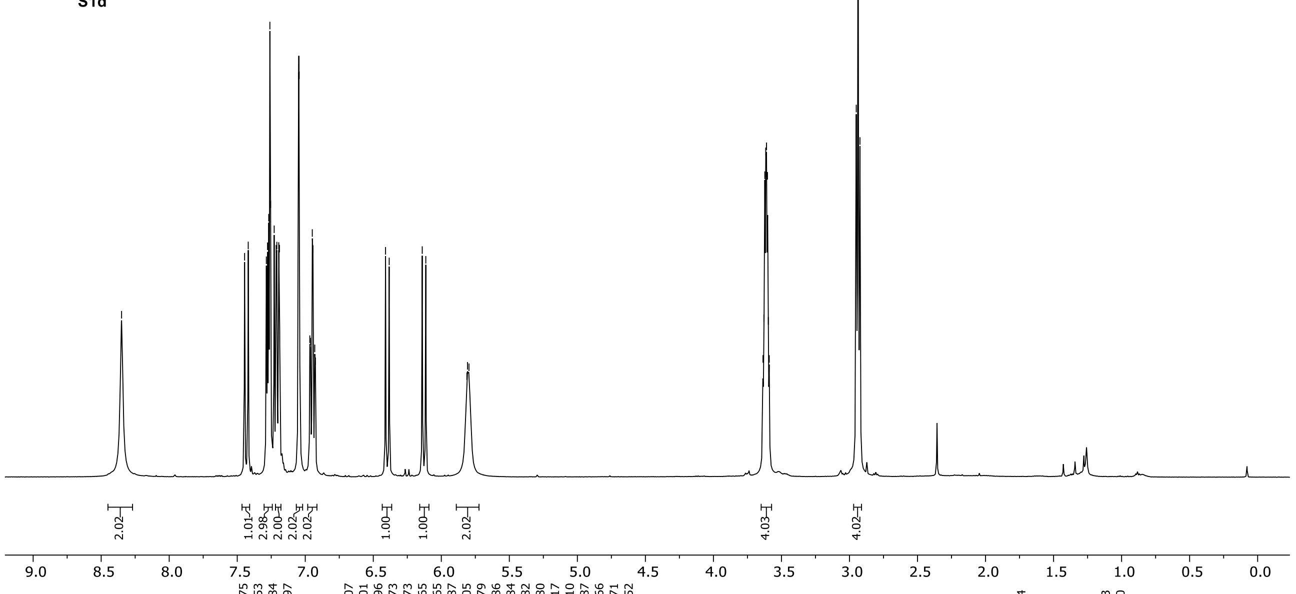
1H-NMR
500 MHz
CDCl3
8.35 7.45 7.42 7.29 7.28 7.27 7.26 7.25 7.23 7.21 7.21 7.19 7.19 7.05 7.04 6.97 6.96 6.95 6.94 6.93 6.93 6.41 6.38 6.14 6.11 5.81 5.81 5.80
3.64 3.63 3.62 3.62 3.61 3.60 3.60 3.59 2.95 2.94 2.92
2.02 1.01 2.98 2.00 2.02 2.02 1.00 1.00 2.02 4.03 4.02
9.0 8.5 8.0 7.5 7.0 6.5 6.0 5.5 5.0 4.5 4.0 3.5 3.0 2.5 2.0 1.5 1.0 0.5 0.0

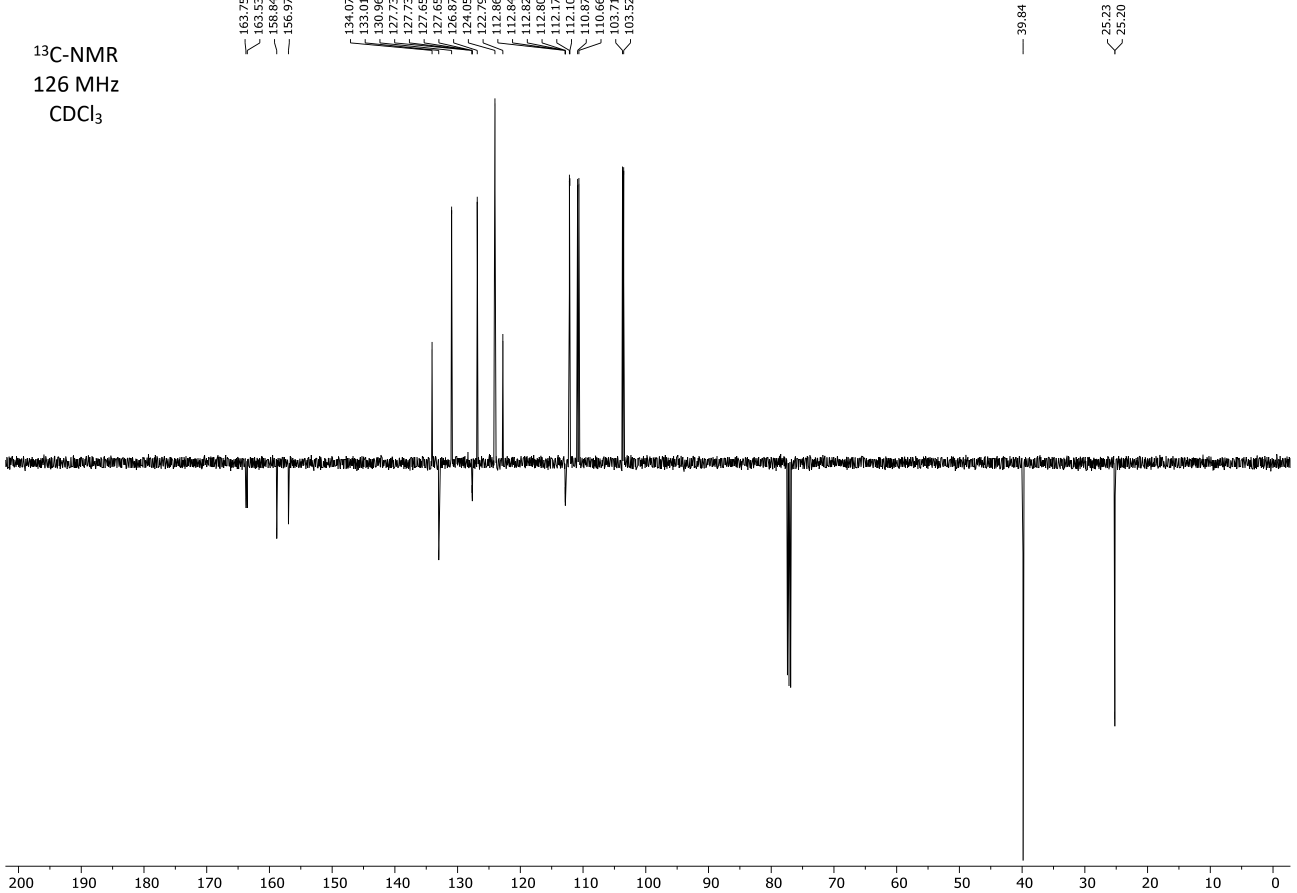
13C-NMR
126 MHz
CDCl3
163.75 163.53 158.84 156.97
134.07 133.01 130.96 127.73 127.73 127.65 126.87 124.05 122.79 112.86 112.84 112.82 112.80 112.17 112.10 110.87 110.66 103.71 103.52
39.84
25.23 25.20
200 190 180 170 160 150 140 130 120 110 100 90 80 70 60 50 40 30 20 10 0

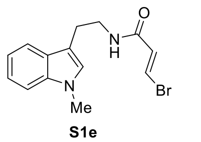

S1e

^{1}H-NMR
500 MHz
$CDCl_3$

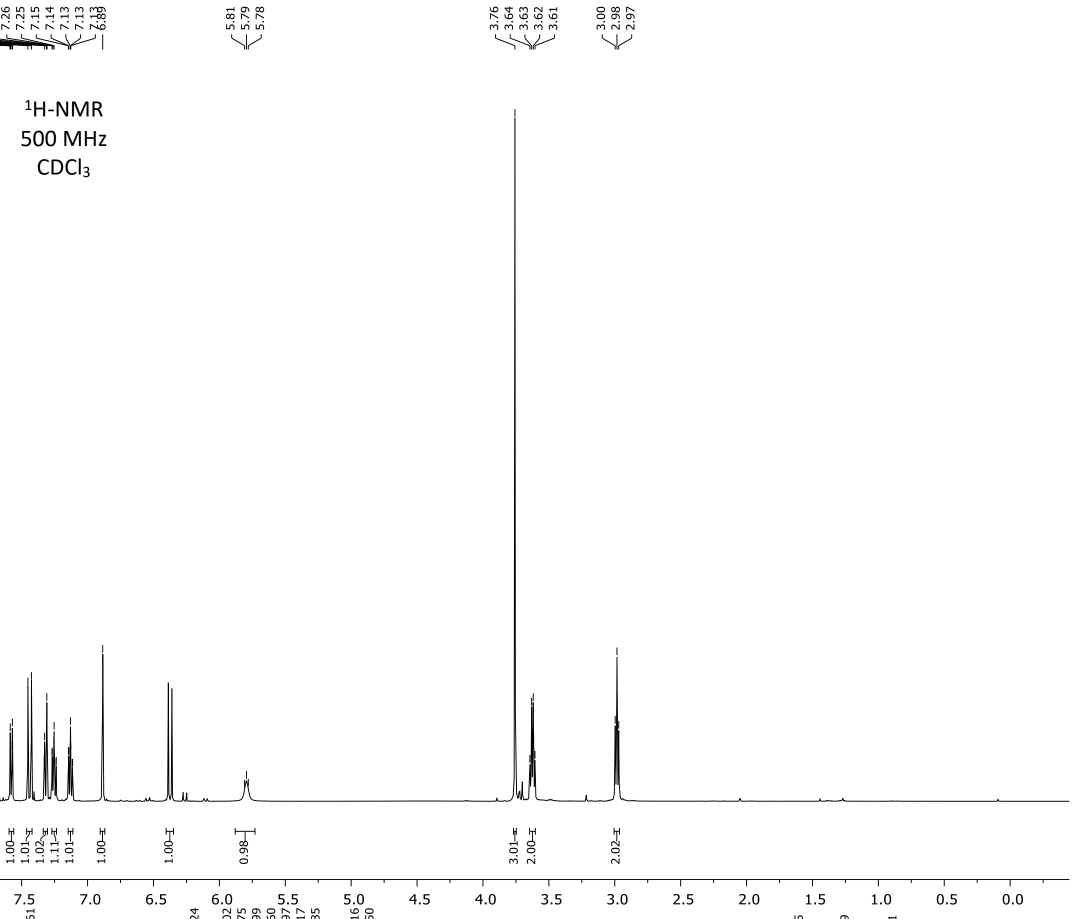

^{13}C-NMR
126 MHz
$CDCl_3$

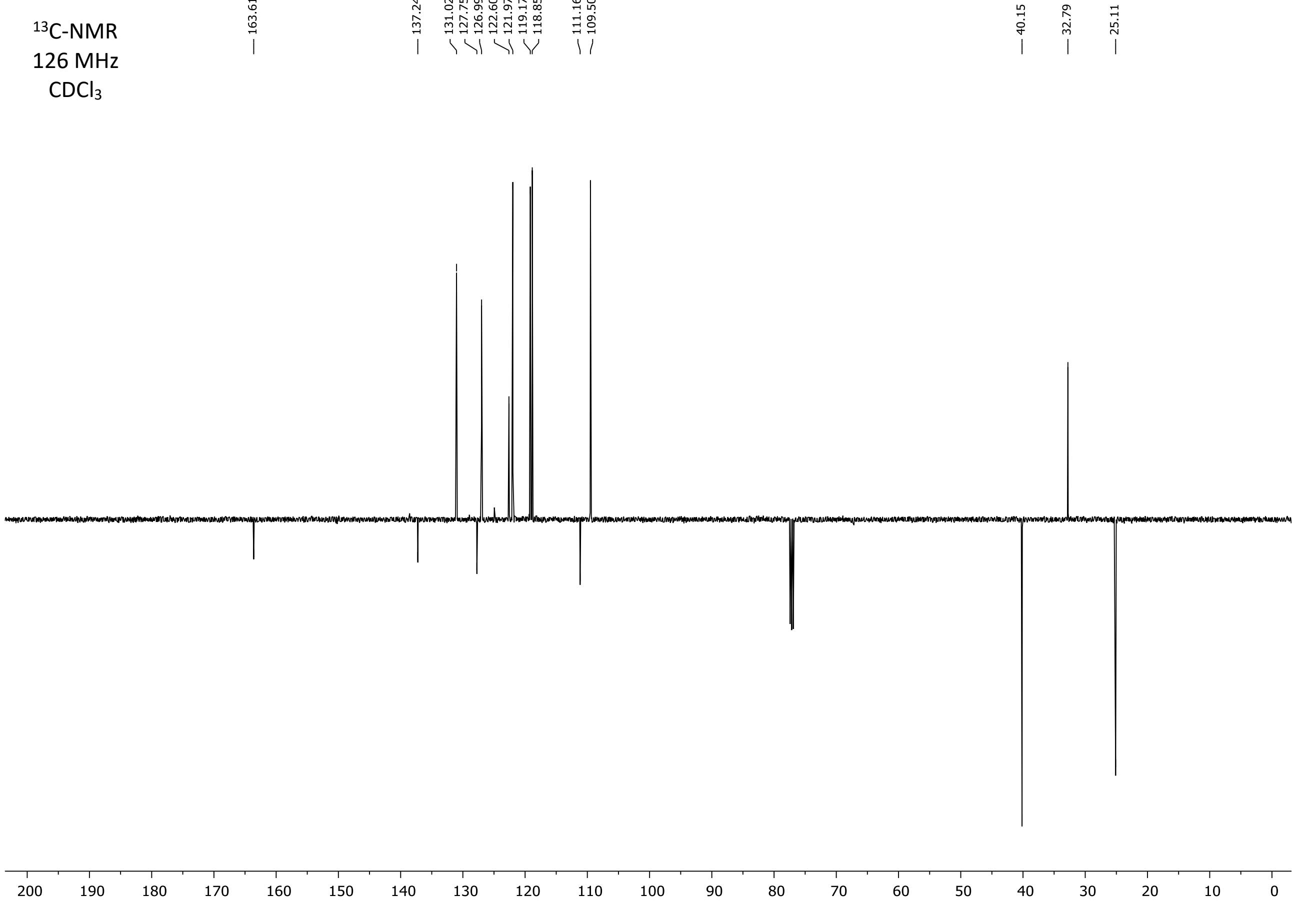

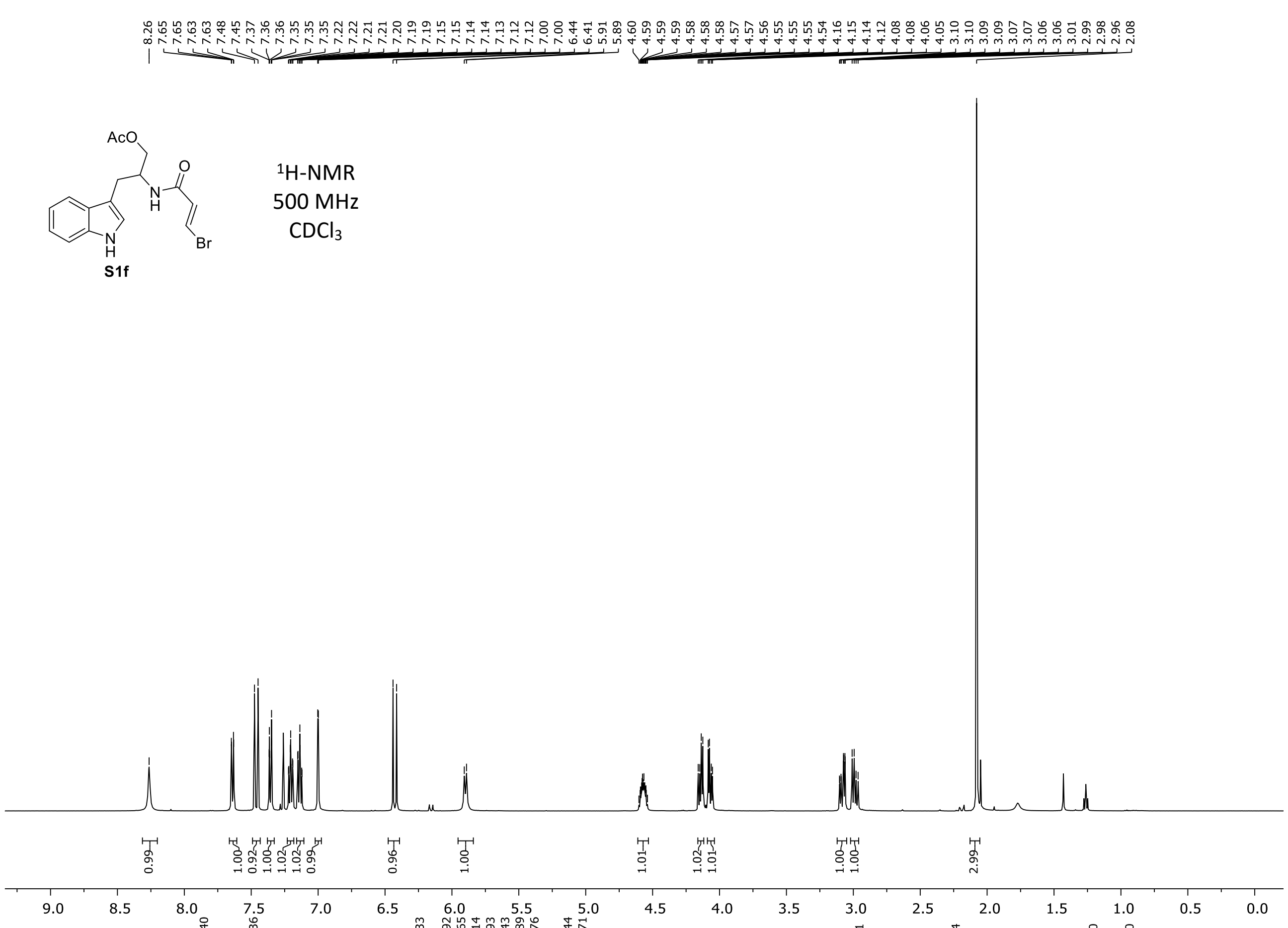
AcO
N
H
O
N
H
Br
S1f
1H-NMR
500 MHz
CDCl3
8.26 7.65 7.63 7.48 7.45 7.37 7.36 7.35 7.35 7.35 7.22 7.22 7.21 7.21 7.20 7.19 7.19 7.15 7.15 7.14 7.14 7.13 7.12 7.12 7.00 6.44 6.41 5.91 5.89 4.60 4.59 4.59 4.59 4.58 4.58 4.58 4.57 4.56 4.55 4.55 4.55 4.54 4.16 4.15 4.14 4.12 4.08 4.08 4.06 4.05 3.10 3.10 3.09 3.07 3.06 3.01 2.99 2.98 2.96 2.08
0.99 1.00 0.92 1.00 1.02 1.02 0.99 0.96 1.00 1.01 1.02 1.01 1.00 1.00 2.99
9.0 8.5 8.0 7.5 7.0 6.5 6.0 5.5 5.0 4.5 4.0 3.5 3.0 2.5 2.0 1.5 1.0 0.5 0.0

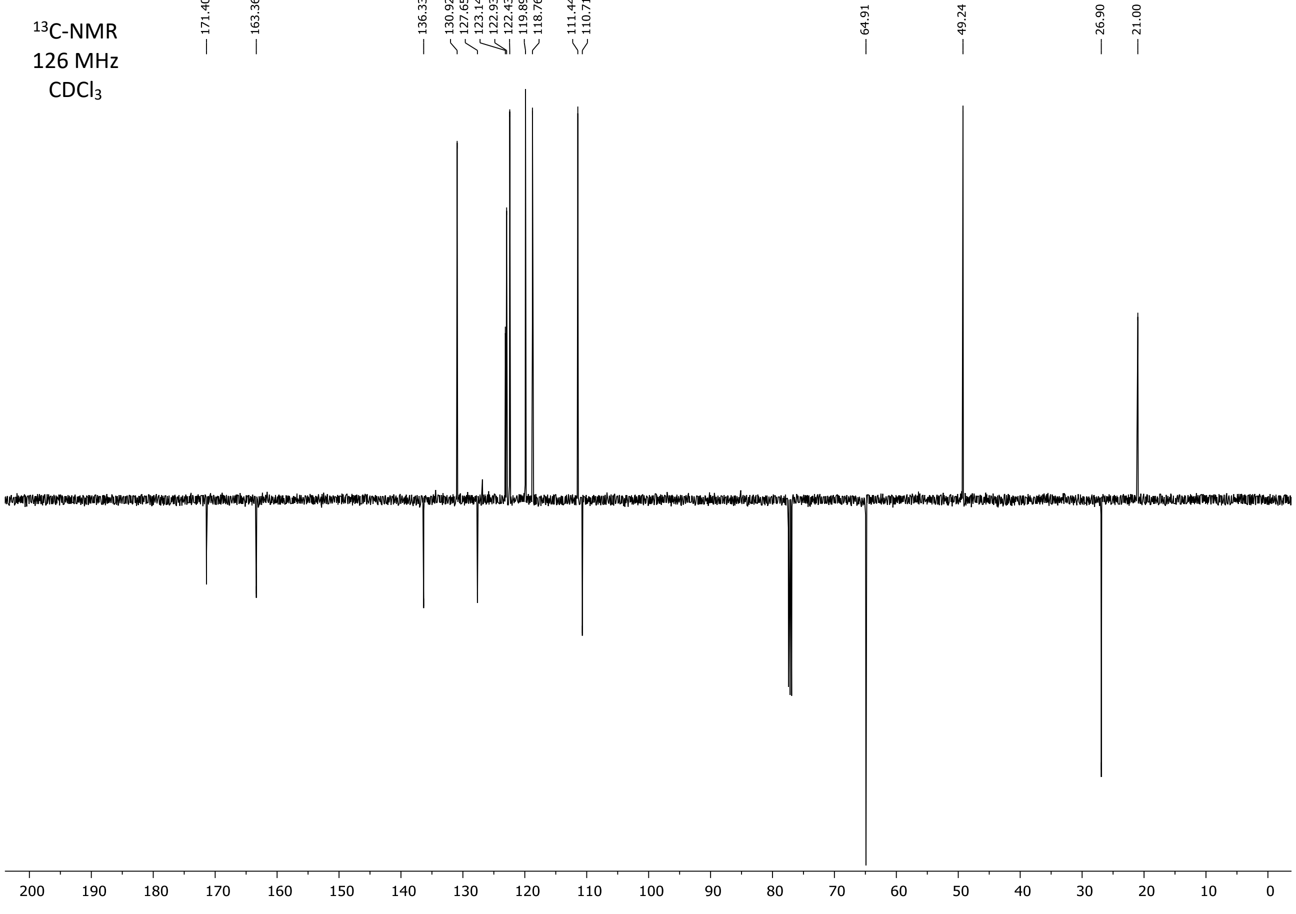
13C-NMR
126 MHz
CDCl3
171.40 163.36 136.33 130.92 127.65 123.14 122.93 122.43 119.89 118.76 111.44 110.71 64.91 49.24 26.90 21.00
200 190 180 170 160 150 140 130 120 110 100 90 80 70 60 50 40 30 20 10 0

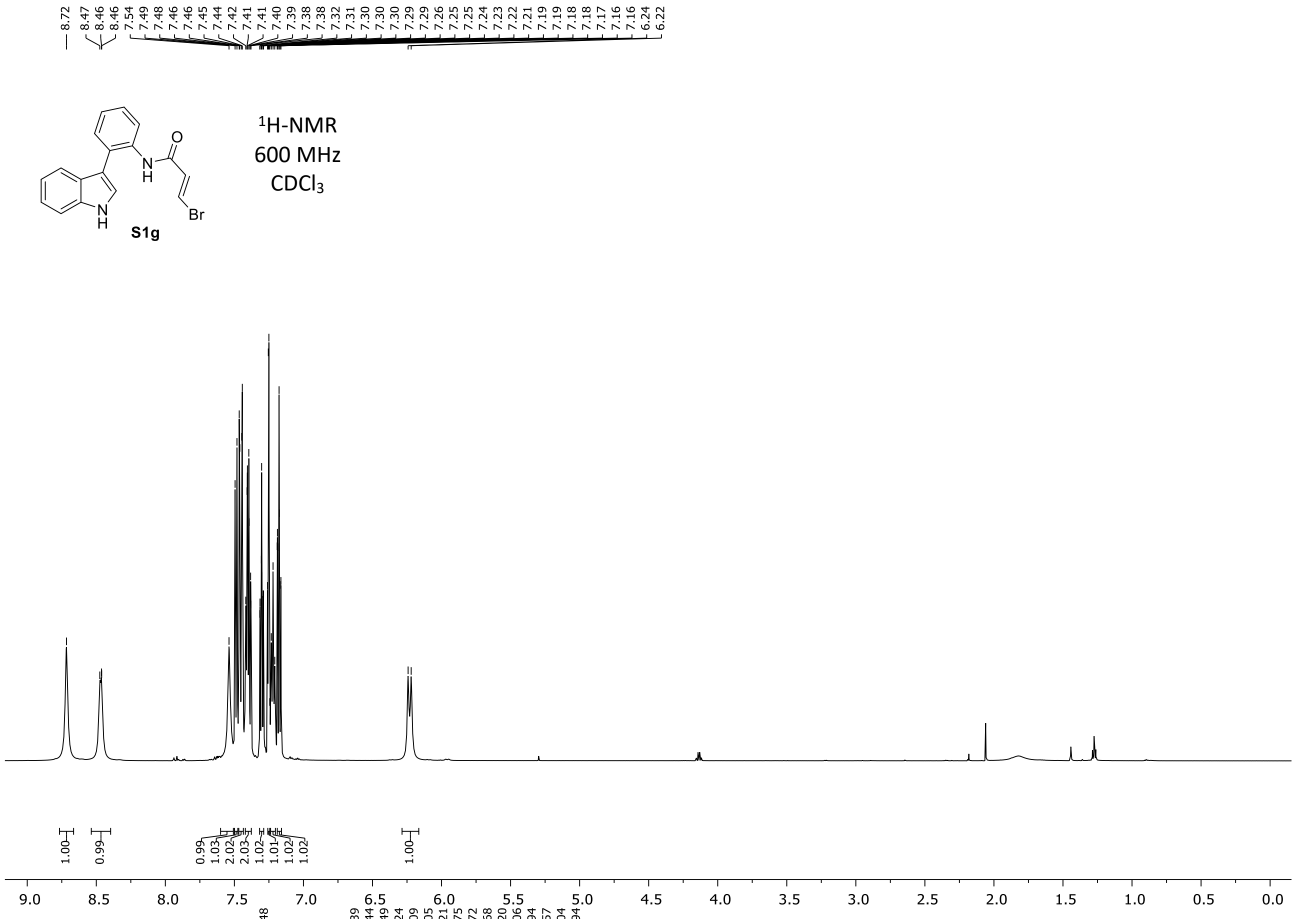

8.72
8.47
8.46
8.46
7.54
7.49
7.48
7.46
7.46
7.45
7.44
7.42
7.41
7.41
7.40
7.39
7.38
7.38
7.32
7.31
7.30
7.30
7.30
7.29
7.29
7.26
7.25
7.25
7.24
7.23
7.22
7.21
7.19
7.19
7.18
7.18
7.17
7.16
7.16
6.24
6.22
O
N
H
N
H
Br
S1g
1H-NMR
600 MHz
CDCl3
1.00
0.99
0.99
1.03
2.02
2.03
1.02
1.01
1.02
1.02
1.00
9.0
8.5
8.0
7.5
7.0
6.5
6.0
5.5
5.0
4.5
4.0
3.5
3.0
2.5
2.0
1.5
1.0
0.5
0.0

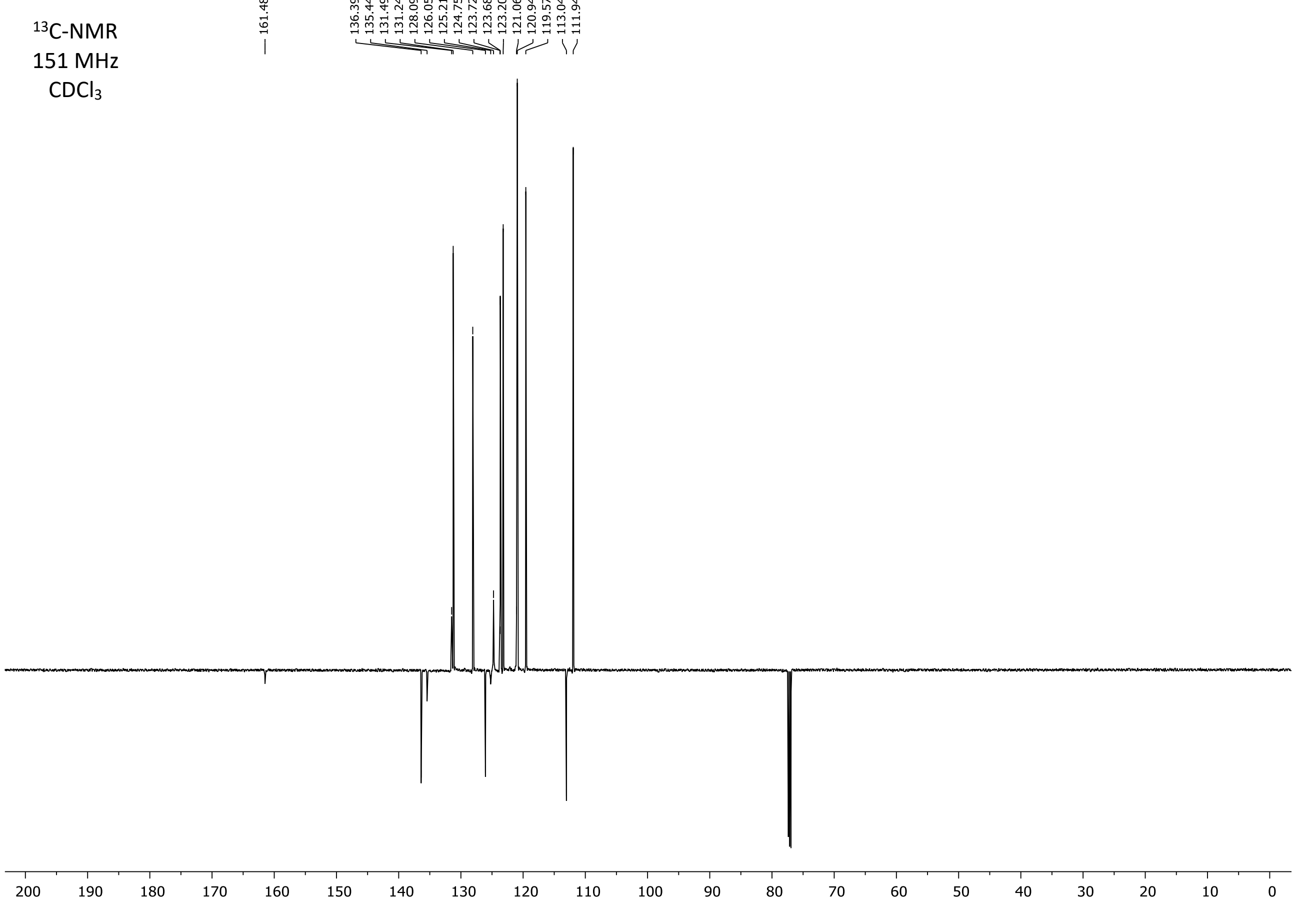

13C-NMR
151 MHz
CDCl3
161.48
136.39
135.44
131.49
131.24
128.09
126.05
125.21
124.75
123.72
123.68
123.20
121.06
120.94
119.57
113.04
111.94
200
190
180
170
160
150
140
130
120
110
100
90
80
70
60
50
40
30
20
10
0

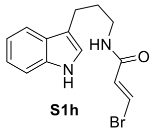

S1h

^{1}H-NMR
500 MHz
$CDCl_3$

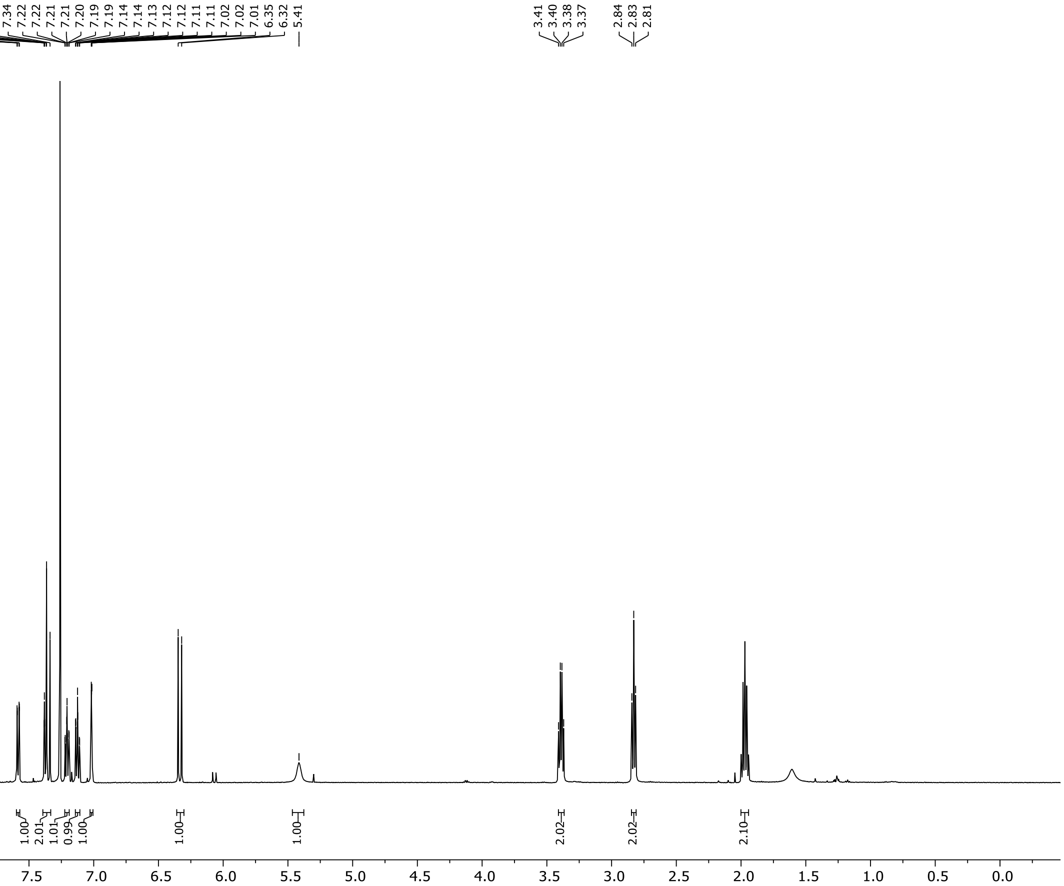

^{13}C-NMR
126 MHz
$CDCl_3$

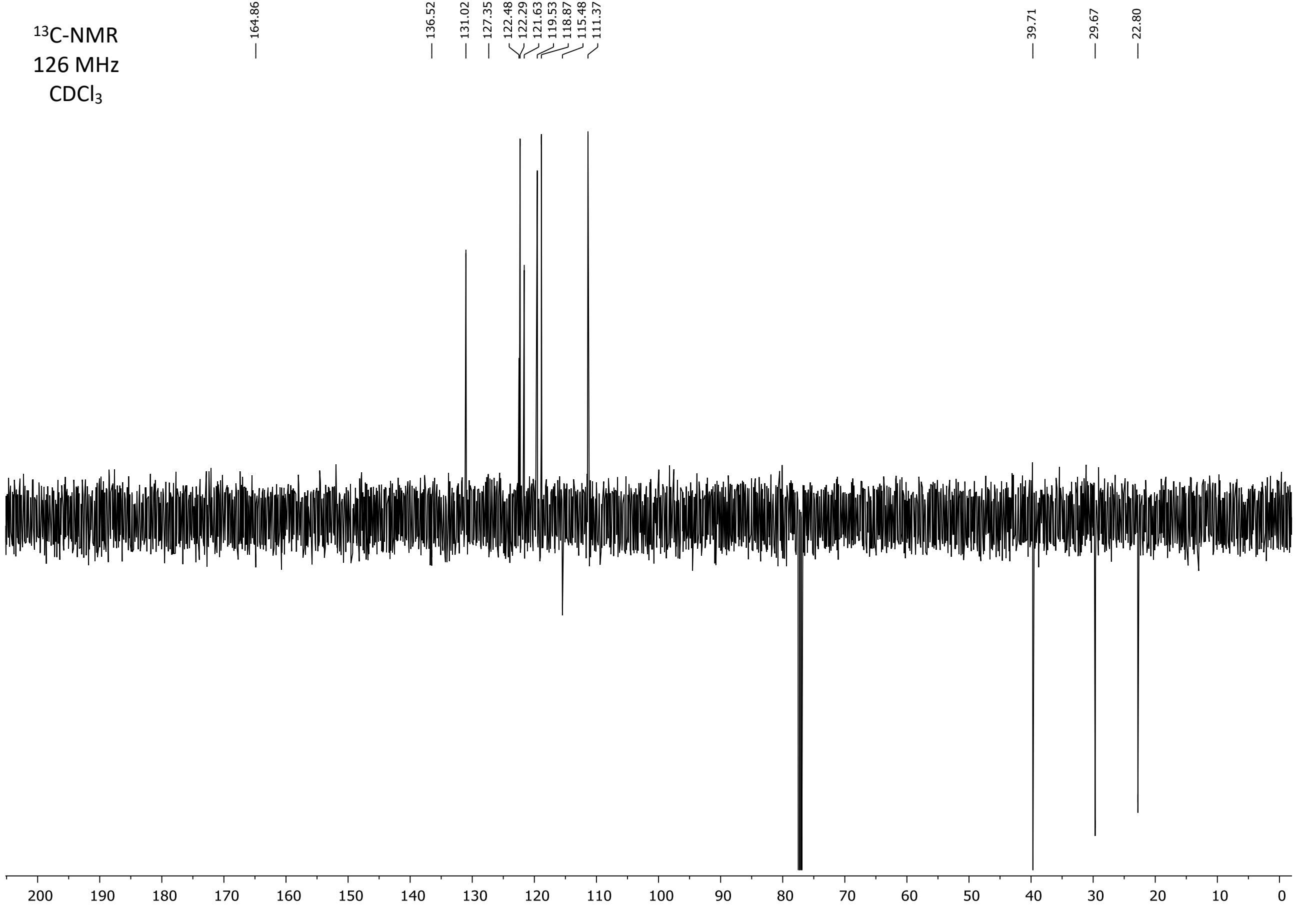

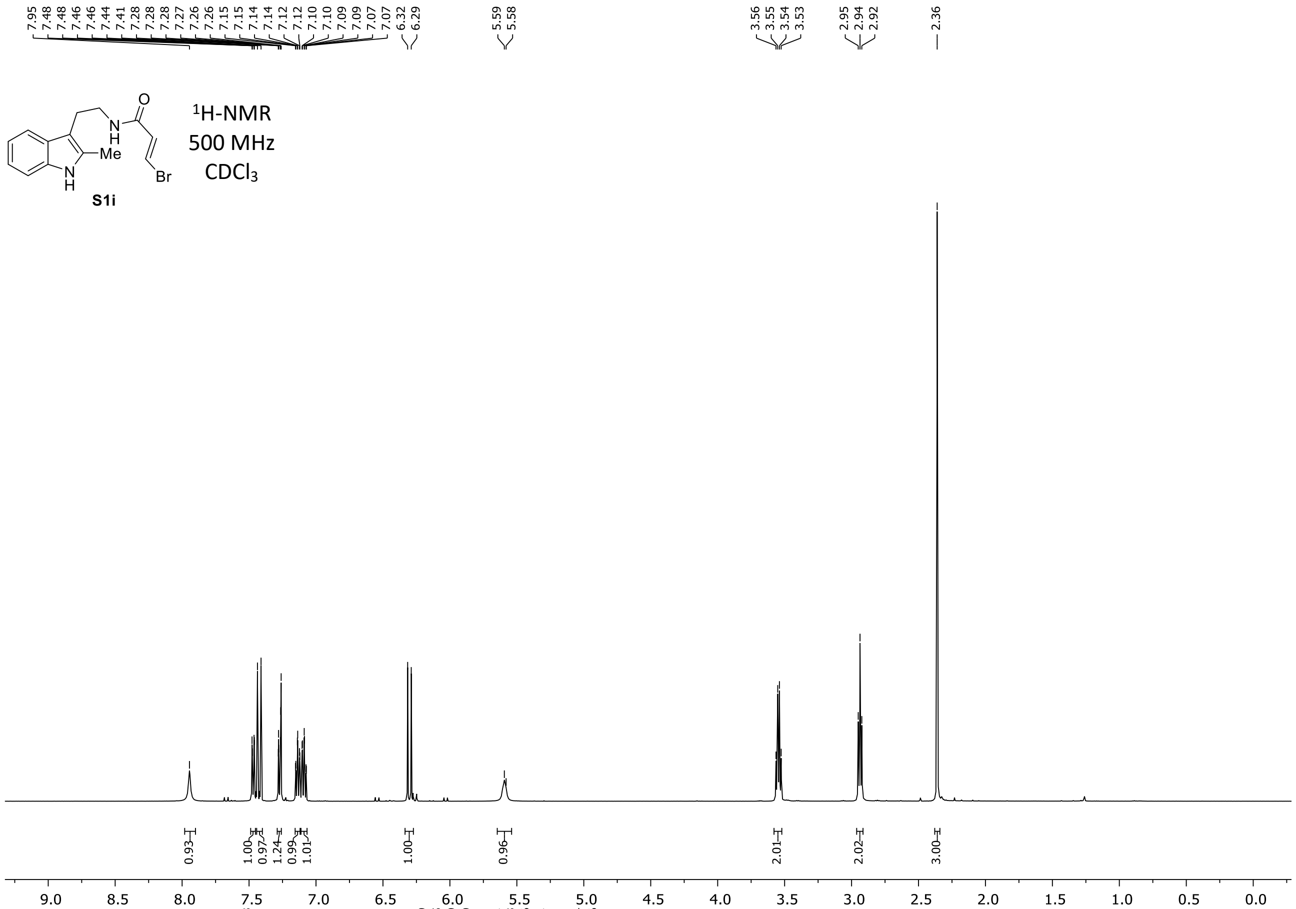

7.95 7.48 7.48 7.46 7.46 7.44 7.41 7.28 7.28 7.28 7.27 7.26 7.26 7.15 7.15 7.14 7.14 7.12 7.12 7.10 7.10 7.09 7.09 7.07 7.07 6.32 6.29
5.59 5.58
3.56 3.55 3.54 3.53
2.95 2.94 2.92
2.36
Me
Br
S1i
1H-NMR
500 MHz
CDCl3
0.93 1.00 0.97 1.24 0.99 1.01 1.00 0.96 2.01 2.02 3.00
9.0 8.5 8.0 7.5 7.0 6.5 6.0 5.5 5.0 4.5 4.0 3.5 3.0 2.5 2.0 1.5 1.0 0.5 0.0

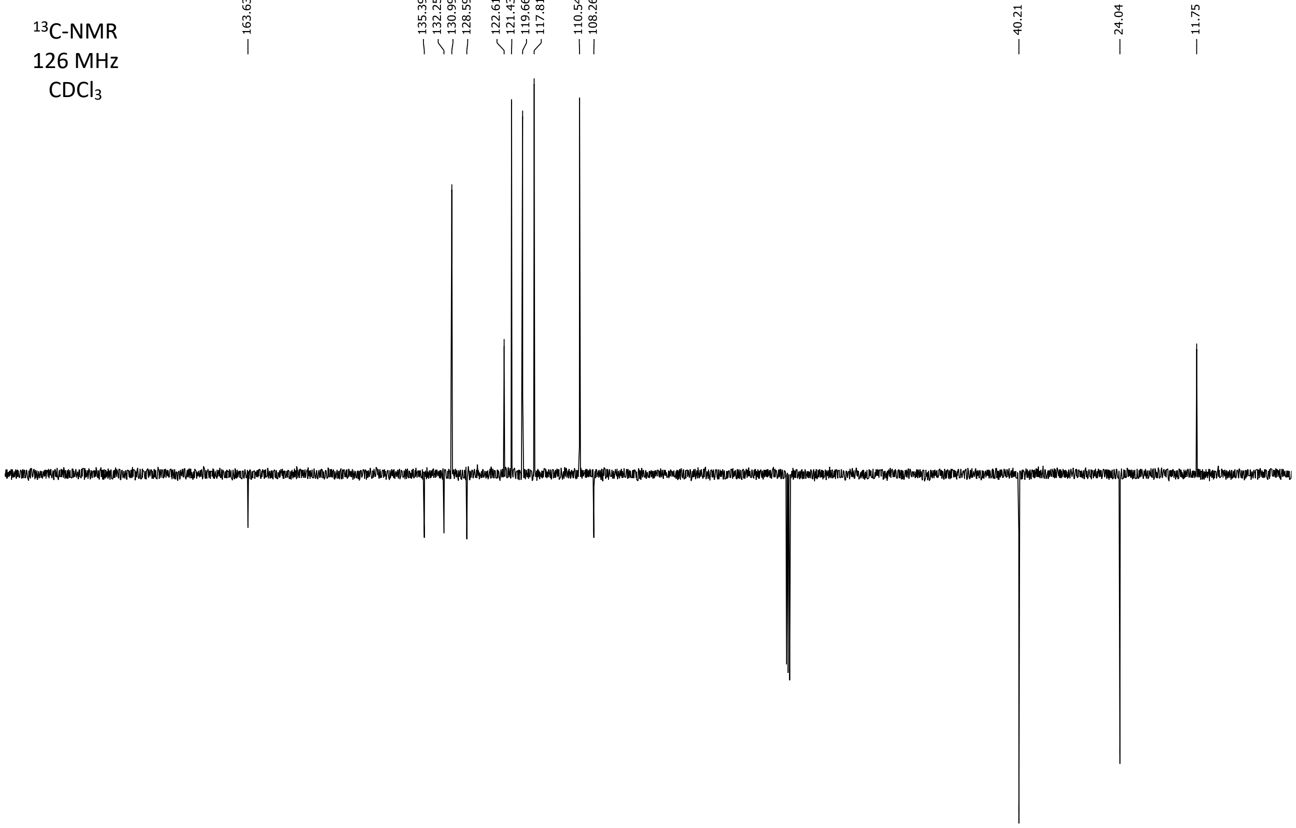

13C-NMR
126 MHz
CDCl3
163.63
135.39 132.25 130.99 128.59
122.61 121.43 119.66 117.81
110.54 108.26
40.21
24.04
11.75
200 190 180 170 160 150 140 130 120 110 100 90 80 70 60 50 40 30 20 10 0

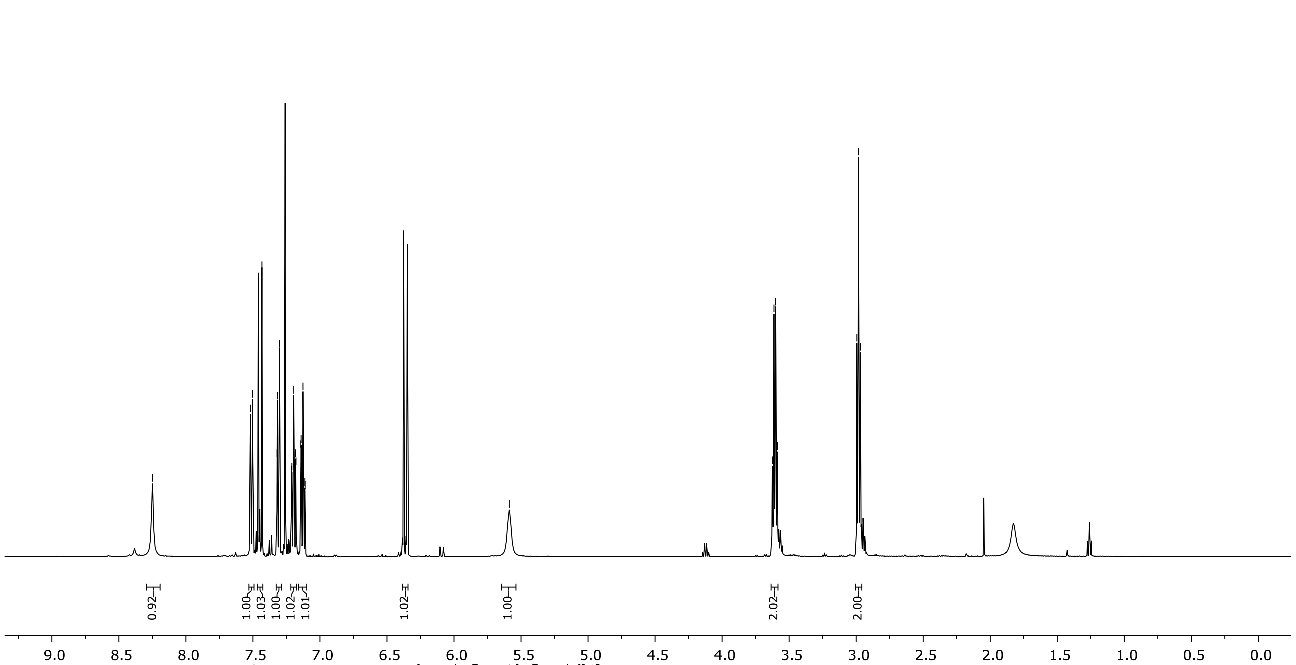

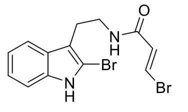

S1j

^{1}H-NMR
500 MHz
$CDCl_3$

^{13}C-NMR
126 MHz
$CDCl_3$

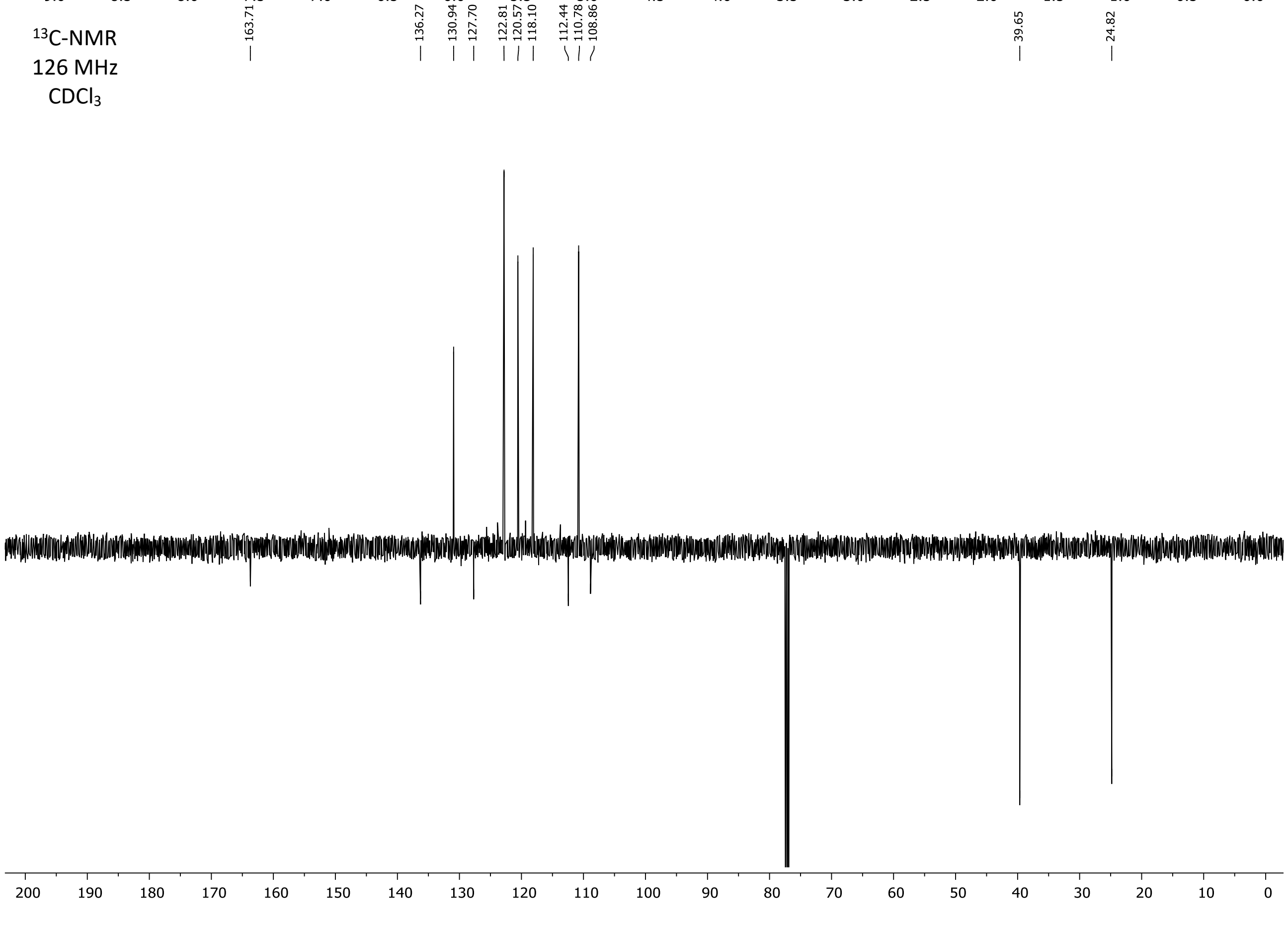

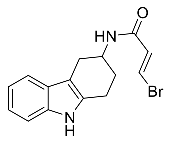

S1k

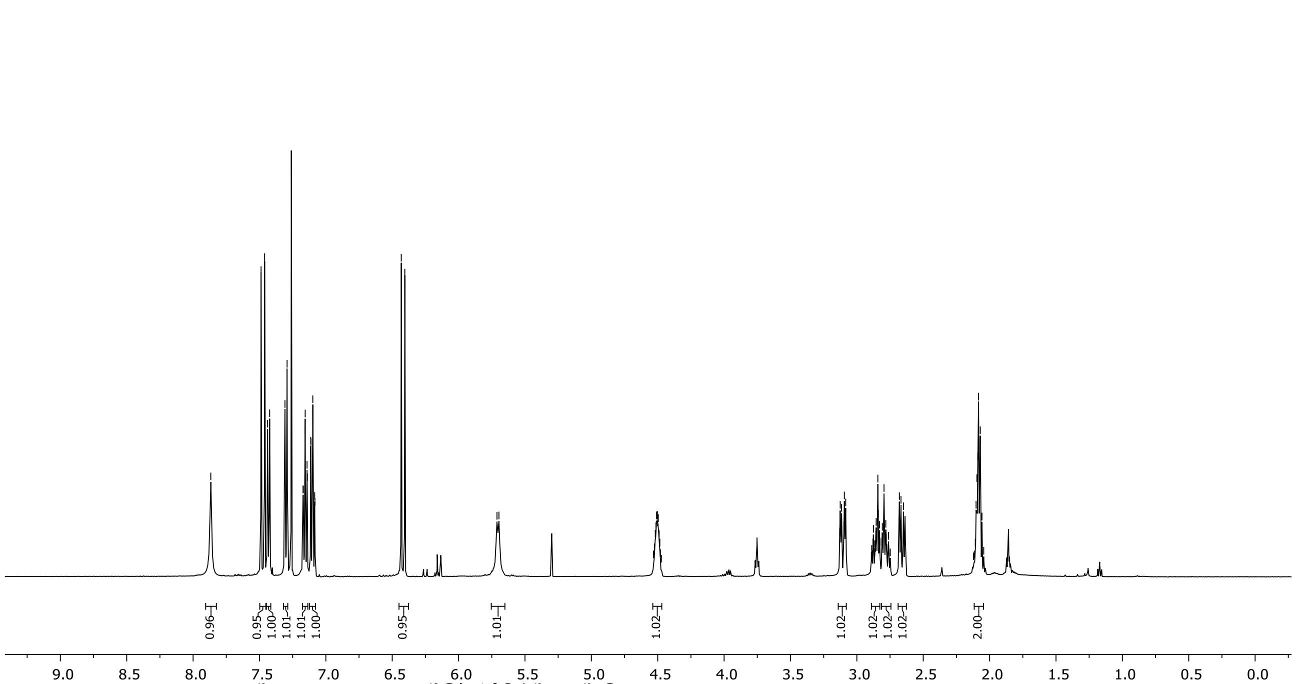
7.87 7.49 7.46 7.44 7.42 7.31 7.29 7.17 7.17 7.16 7.16 7.14 7.14 7.11 7.11 7.10 7.08 7.08 6.43 6.41 5.71 5.70 4.53 4.52 4.51 4.51 4.51 4.50 4.50 4.49 4.48 4.48 3.13 3.13 3.12 3.12 3.10 3.09 3.09 3.08 2.89 2.89 2.88 2.88 2.87 2.86 2.86 2.85 2.85 2.84 2.84 2.84 2.83 2.83 2.83 2.83 2.81 2.80 2.78 2.78 2.77 2.77 2.76 2.75 2.68 2.68 2.68 2.67 2.65 2.12 2.11 2.10 2.10 2.09 2.08 2.08 2.07 2.06 2.04
^{1}H-NMR
500 MHz
$CDCl_3$
0.96 0.95 1.00 1.01 1.01 1.00 0.95 1.01 1.02 1.02 1.02 1.02 1.02 2.00
9.0 8.5 8.0 7.5 7.0 6.5 6.0 5.5 5.0 4.5 4.0 3.5 3.0 2.5 2.0 1.5 1.0 0.5 0.0

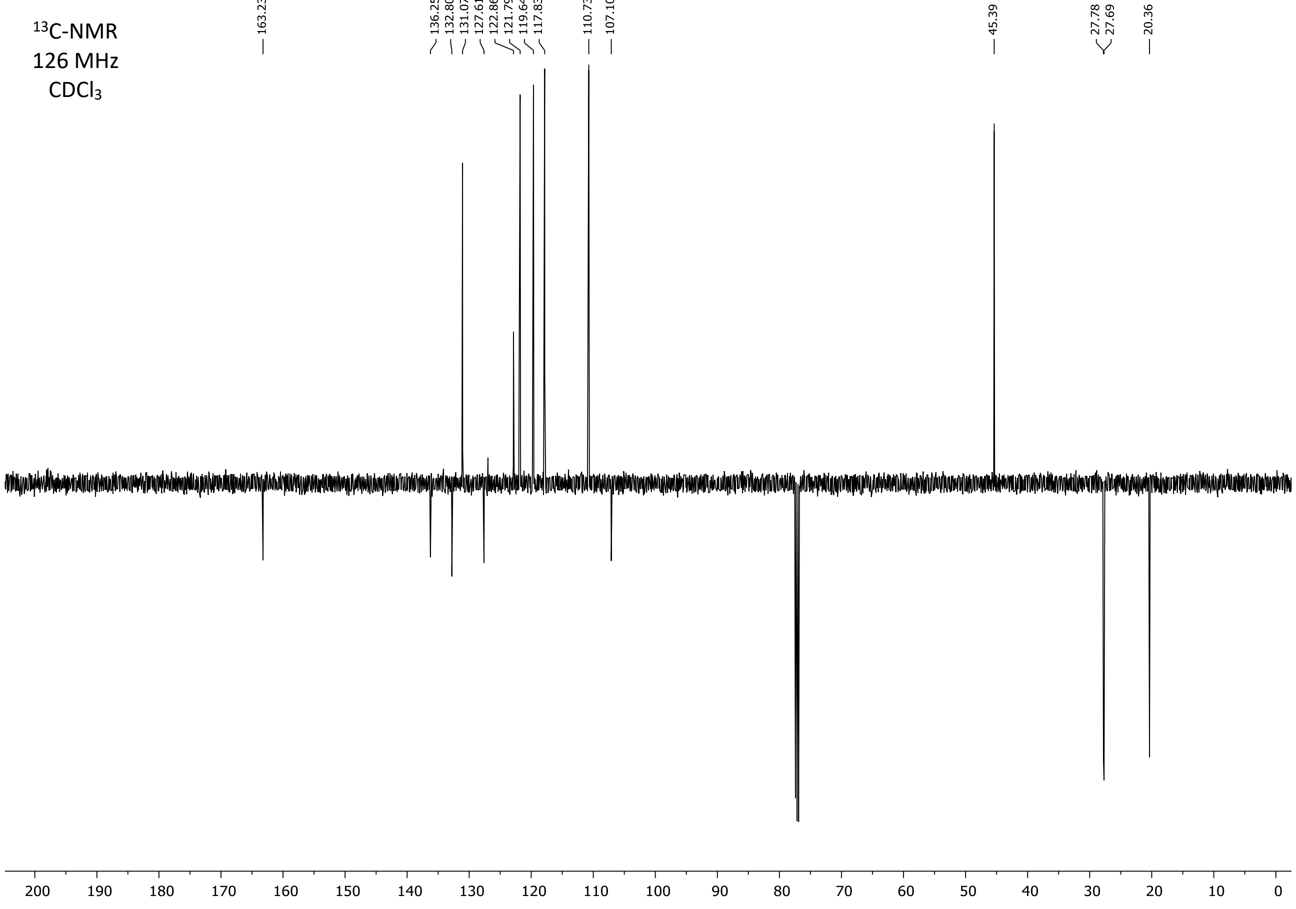
^{13}C-NMR
126 MHz
$CDCl_3$
163.23
136.25 132.80 131.07 127.61 122.86 121.79 119.64 117.83
110.73 107.10
45.39
27.78 27.69
20.36
200 190 180 170 160 150 140 130 120 110 100 90 80 70 60 50 40 30 20 10 0

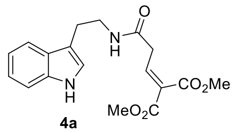

4a

^{1}H-NMR
500 MHz
$CDCl_3$

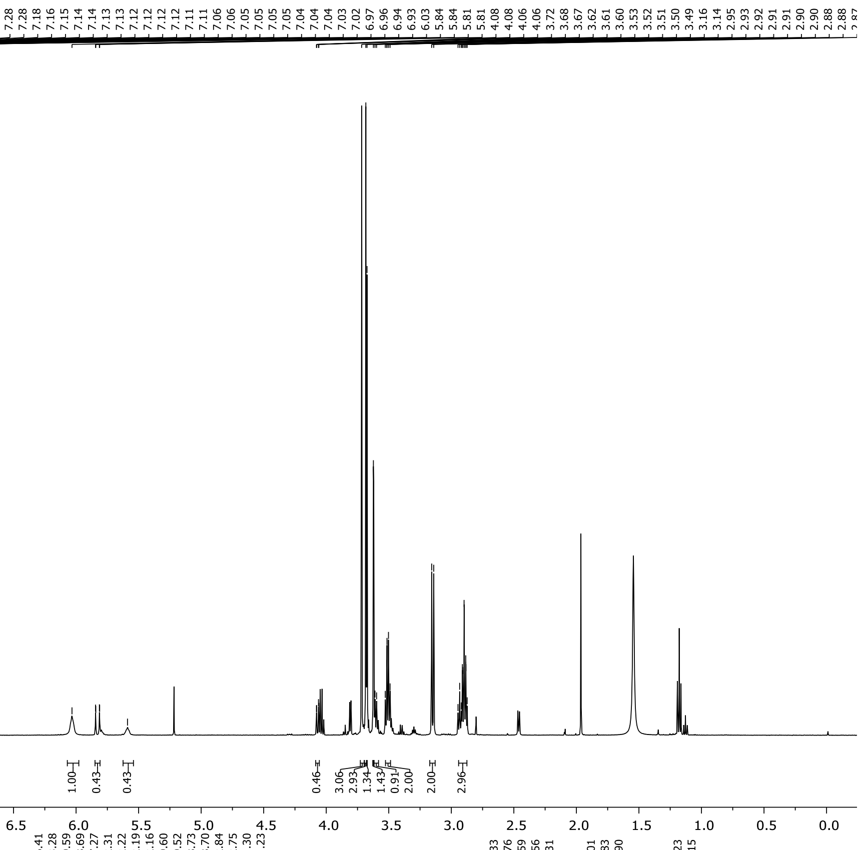

^{13}C-NMR
126 MHz
$CDCl_3$

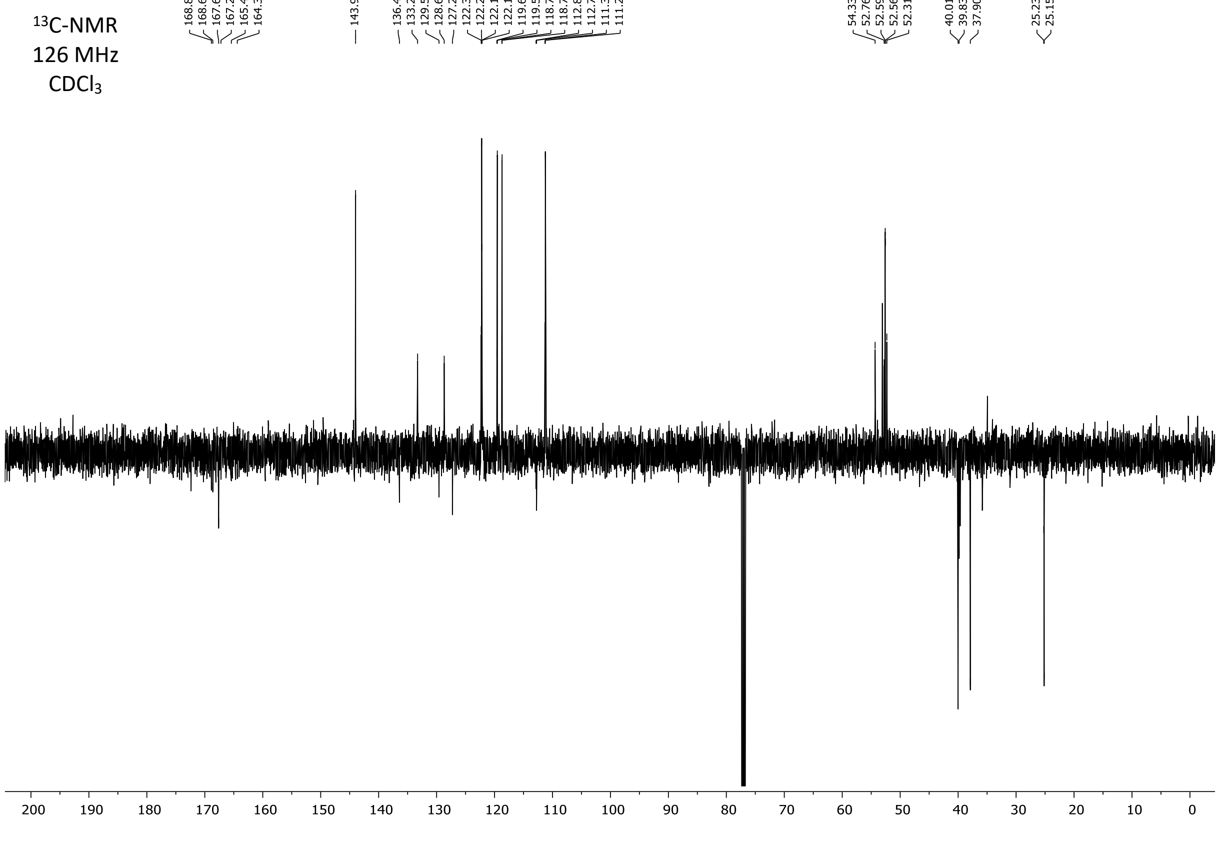

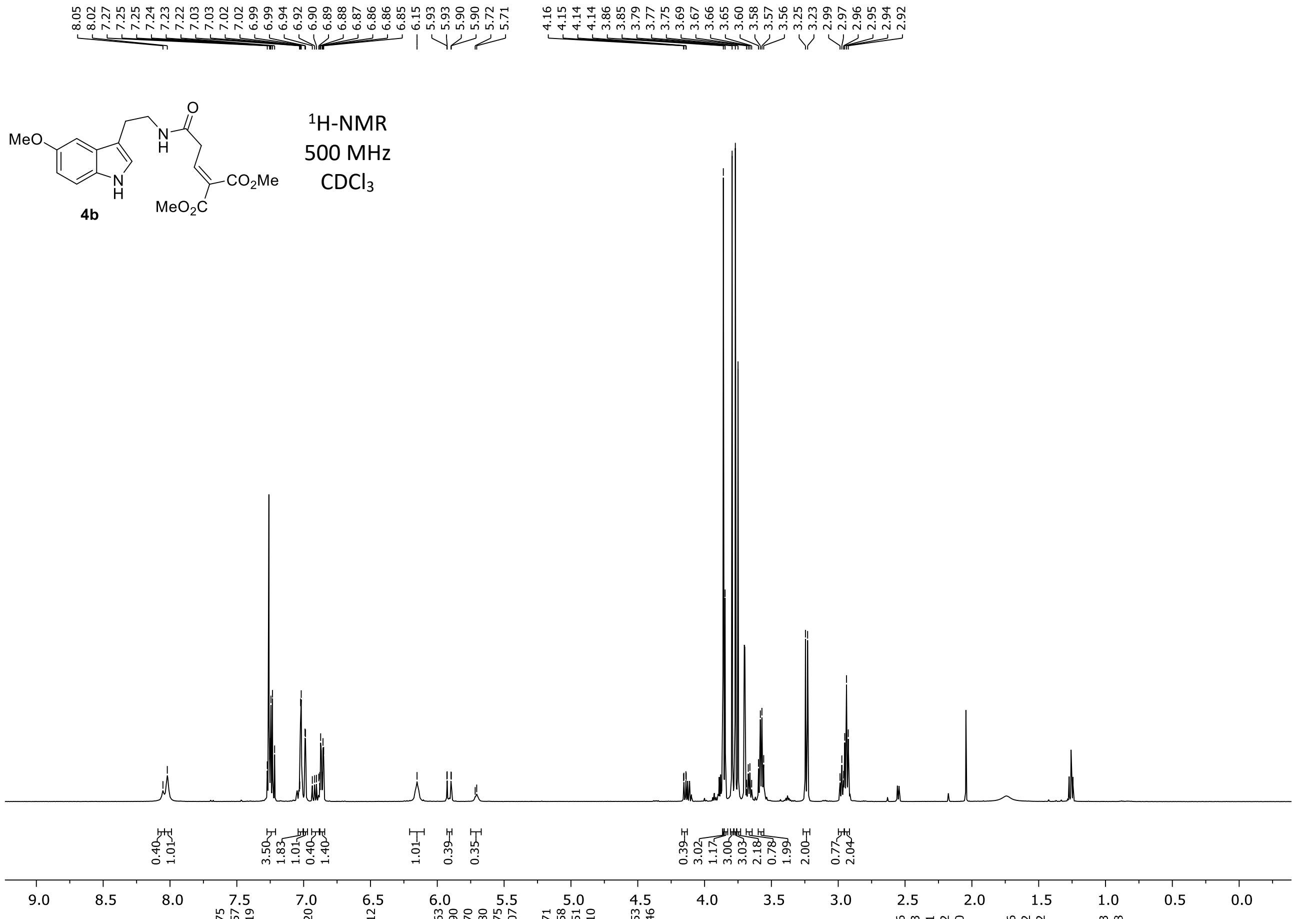
8.05 8.02 7.27 7.25 7.25 7.24 7.23 7.22 7.03 7.03 7.02 6.99 6.99 6.94 6.92 6.90 6.89 6.88 6.87 6.86 6.86 6.85 6.15 5.93 5.93 5.90 5.90 5.72 5.71
4.16 4.15 4.14 4.14 3.86 3.85 3.79 3.77 3.75 3.69 3.67 3.66 3.65 3.60 3.58 3.57 3.56 3.25 3.23 2.99 2.97 2.96 2.95 2.94 2.92
MeO
N
H
O
N
H
CO_2Me
MeO_2C
4b
^{1}H-NMR
500 MHz
$CDCl_3$
0.40 1.01 3.50 1.83 1.01 0.40 1.40 1.01 0.39 0.35 0.39 3.02 1.17 3.00 3.03 2.18 0.78 1.99 2.00 0.77 2.04
9.0 8.5 8.0 7.5 7.0 6.5 6.0 5.5 5.0 4.5 4.0 3.5 3.0 2.5 2.0 1.5 1.0 0.5 0.0

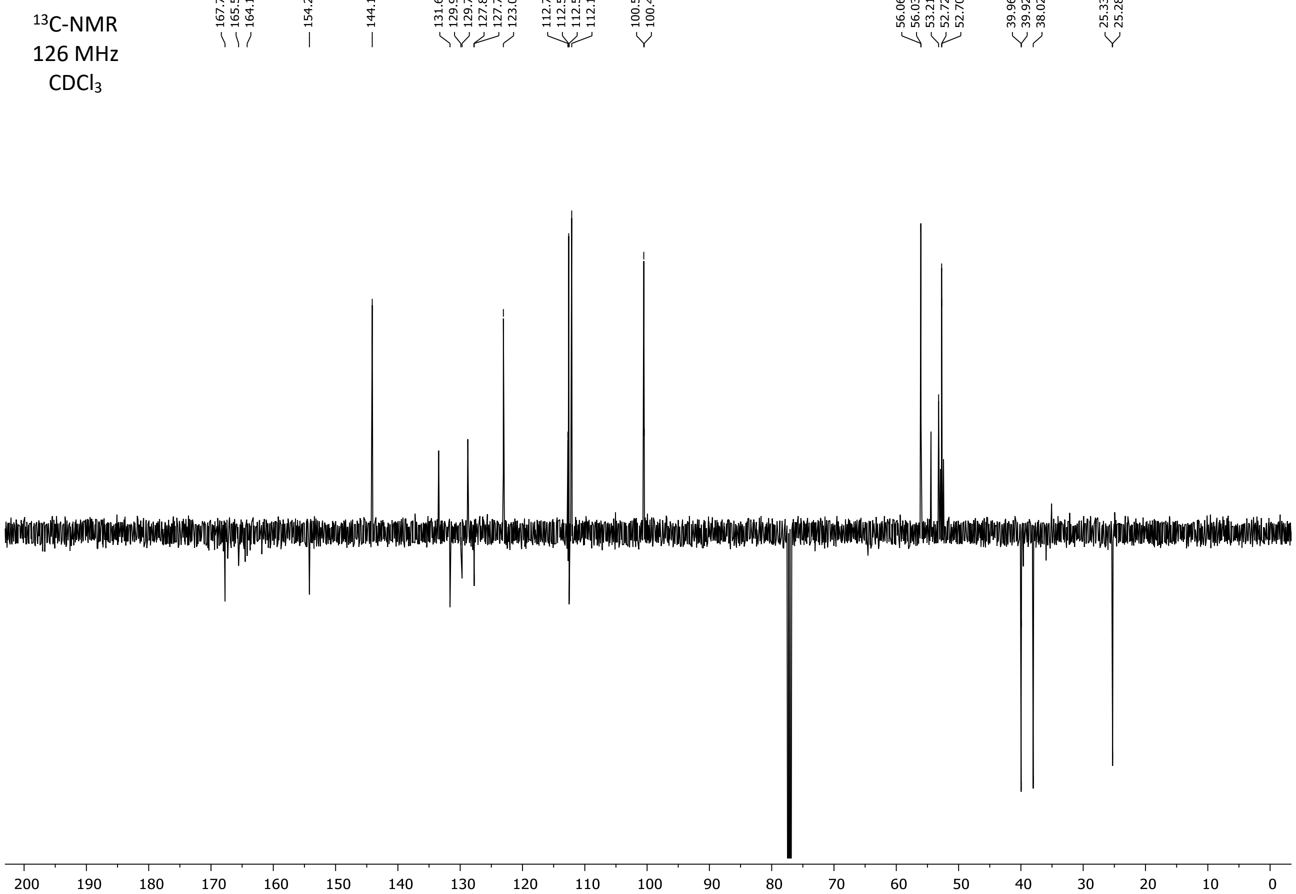
^{13}C-NMR
126 MHz
$CDCl_3$
167.75 165.57 164.19 154.20 144.12 131.63 129.90 129.70 127.80 127.75 123.07 112.71 112.58 112.51 112.10 100.53 100.46 56.06 56.03 53.21 52.72 52.70 39.96 39.92 38.02 25.33 25.28
200 190 180 170 160 150 140 130 120 110 100 90 80 70 60 50 40 30 20 10 0

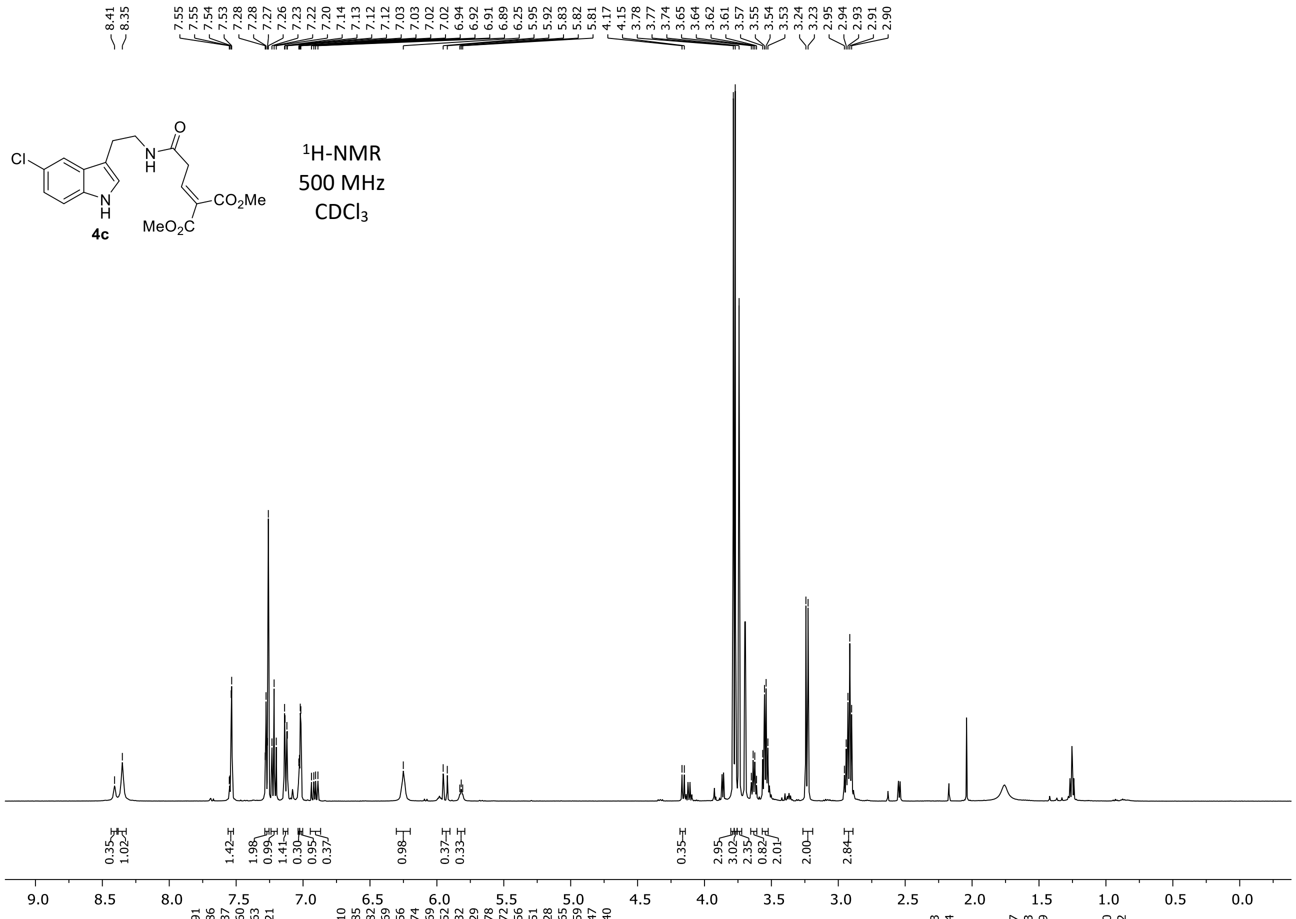

4c

^{1}H-NMR
500 MHz
$CDCl_3$

^{13}C-NMR
126 MHz
$CDCl_3$

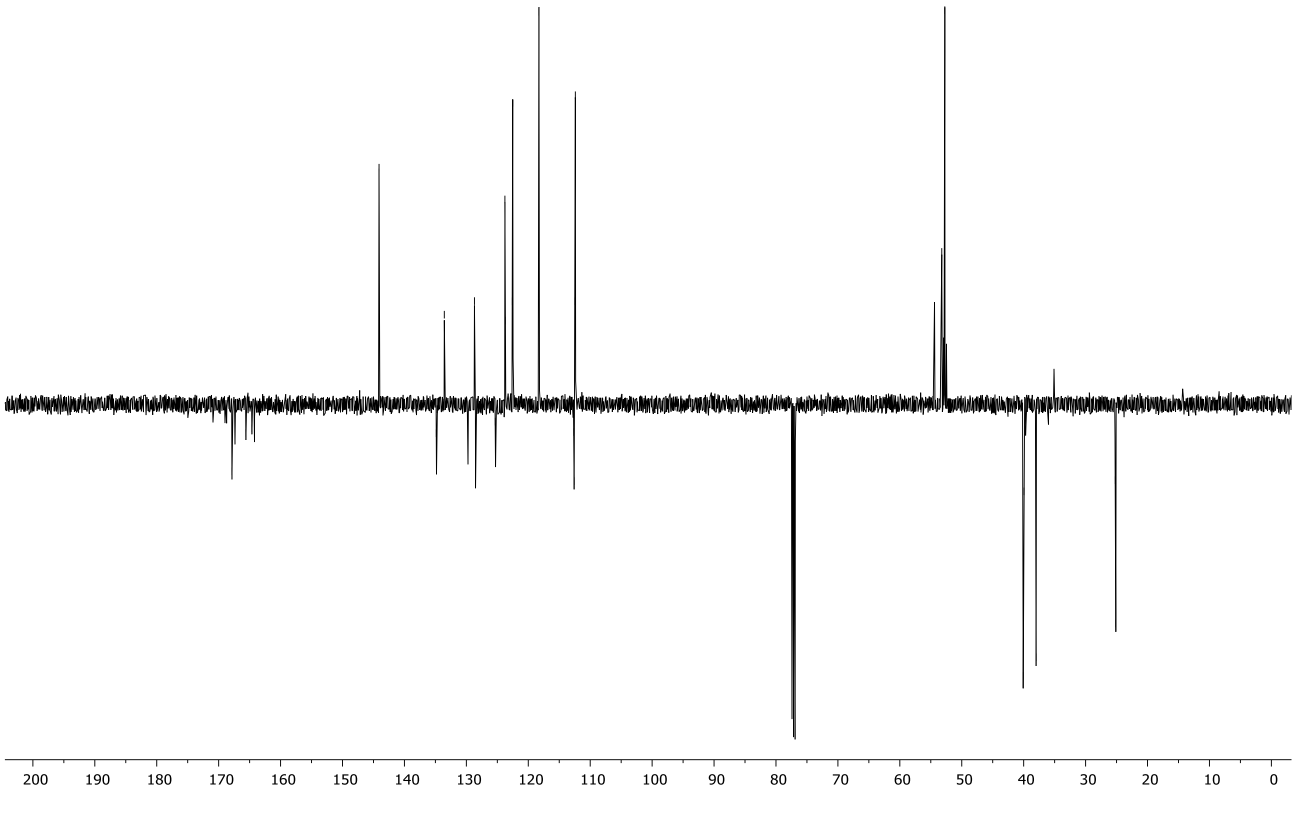

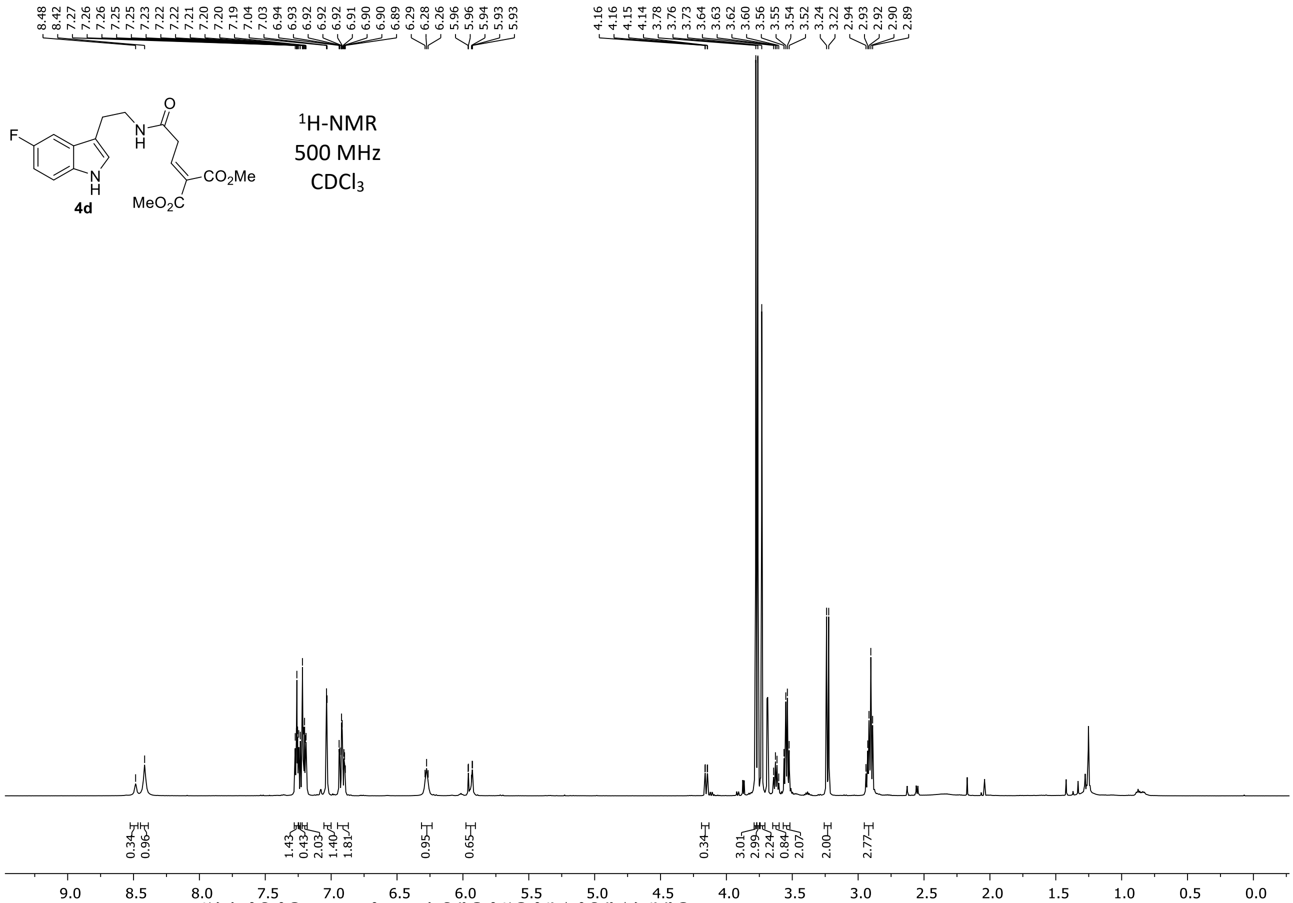

F
N H
O
CO2Me
MeO2C
H
4d
1H-NMR
500 MHz
CDCl3
8.48 8.42 7.27 7.26 7.26 7.25 7.23 7.22 7.22 7.21 7.20 7.19 7.04 7.03 6.94 6.93 6.92 6.92 6.92 6.91 6.90 6.90 6.89 6.29 6.28 6.26 5.96 5.96 5.94 5.93 5.93
4.16 4.16 4.15 4.14 3.78 3.76 3.73 3.64 3.63 3.62 3.60 3.56 3.55 3.54 3.52 3.24 3.22 2.94 2.93 2.92 2.90 2.89
0.34 0.96 1.43 0.43 2.03 1.40 1.81 0.95 0.65 0.34 3.01 2.99 2.24 0.84 2.07 2.00 2.77
9.0 8.5 8.0 7.5 7.0 6.5 6.0 5.5 5.0 4.5 4.0 3.5 3.0 2.5 2.0 1.5 1.0 0.5 0.0

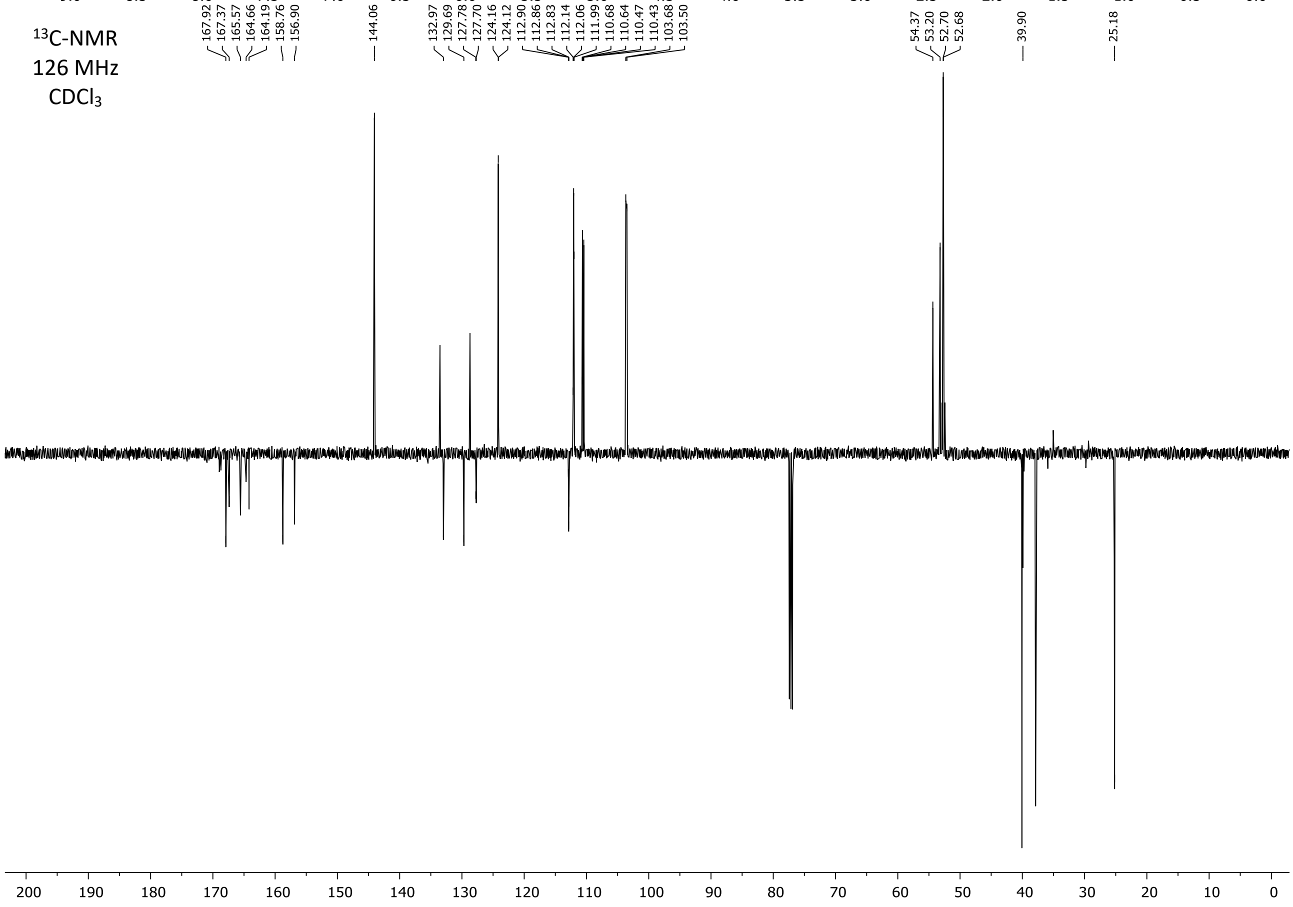

13C-NMR
126 MHz
CDCl3
167.92 167.37 165.57 164.66 164.19 158.76 156.90 144.06 132.97 129.69 127.78 127.70 124.16 124.12 112.90 112.86 112.83 112.14 112.06 111.99 110.68 110.64 110.47 110.43 103.68 103.50
54.37 53.20 52.70 52.68 39.90 25.18
200 190 180 170 160 150 140 130 120 110 100 90 80 70 60 50 40 30 20 10 0

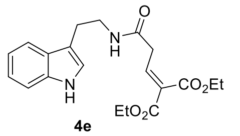

4e

^{1}H-NMR
600 MHz
$CDCl_3$

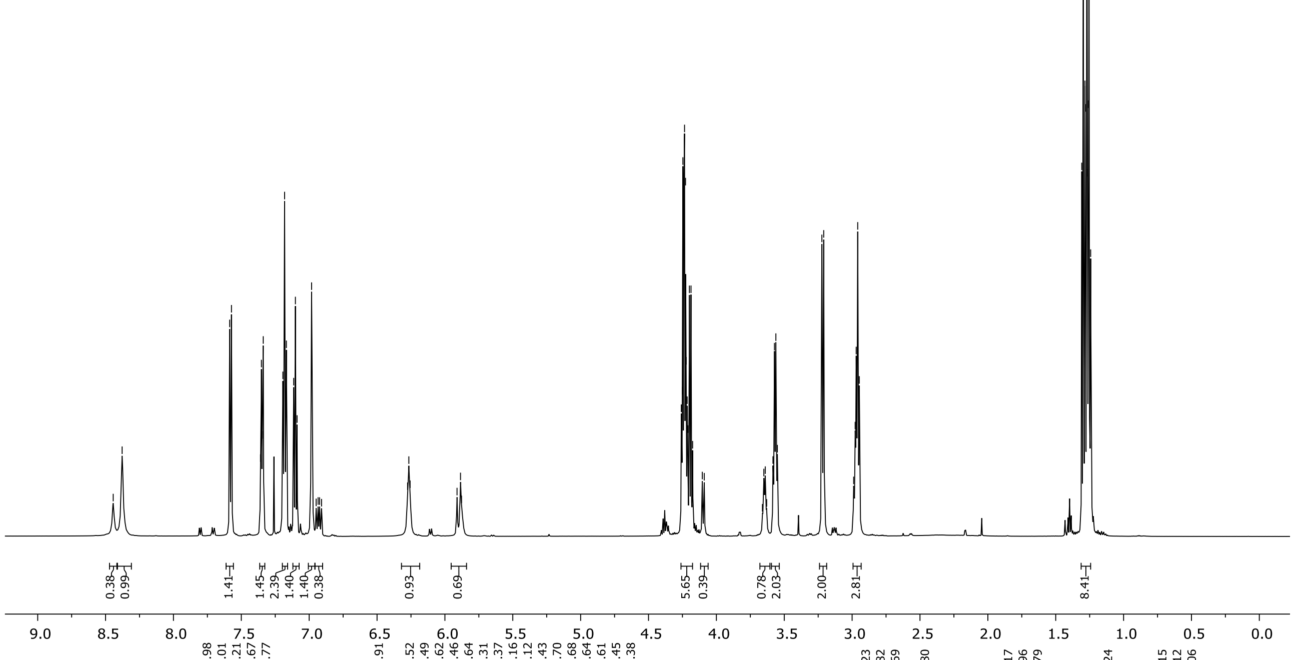

^{13}C-NMR
151 MHz
$CDCl_3$

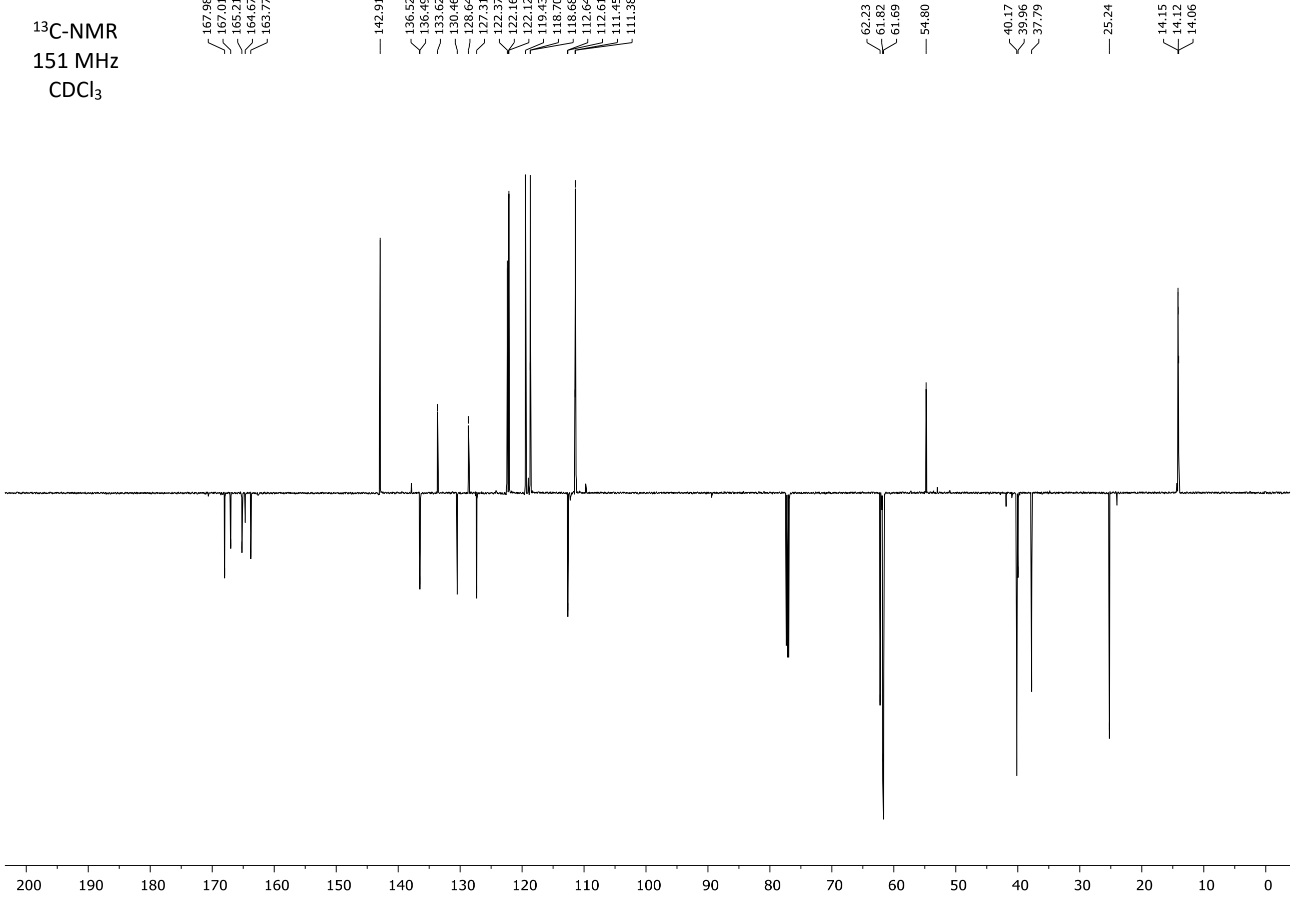

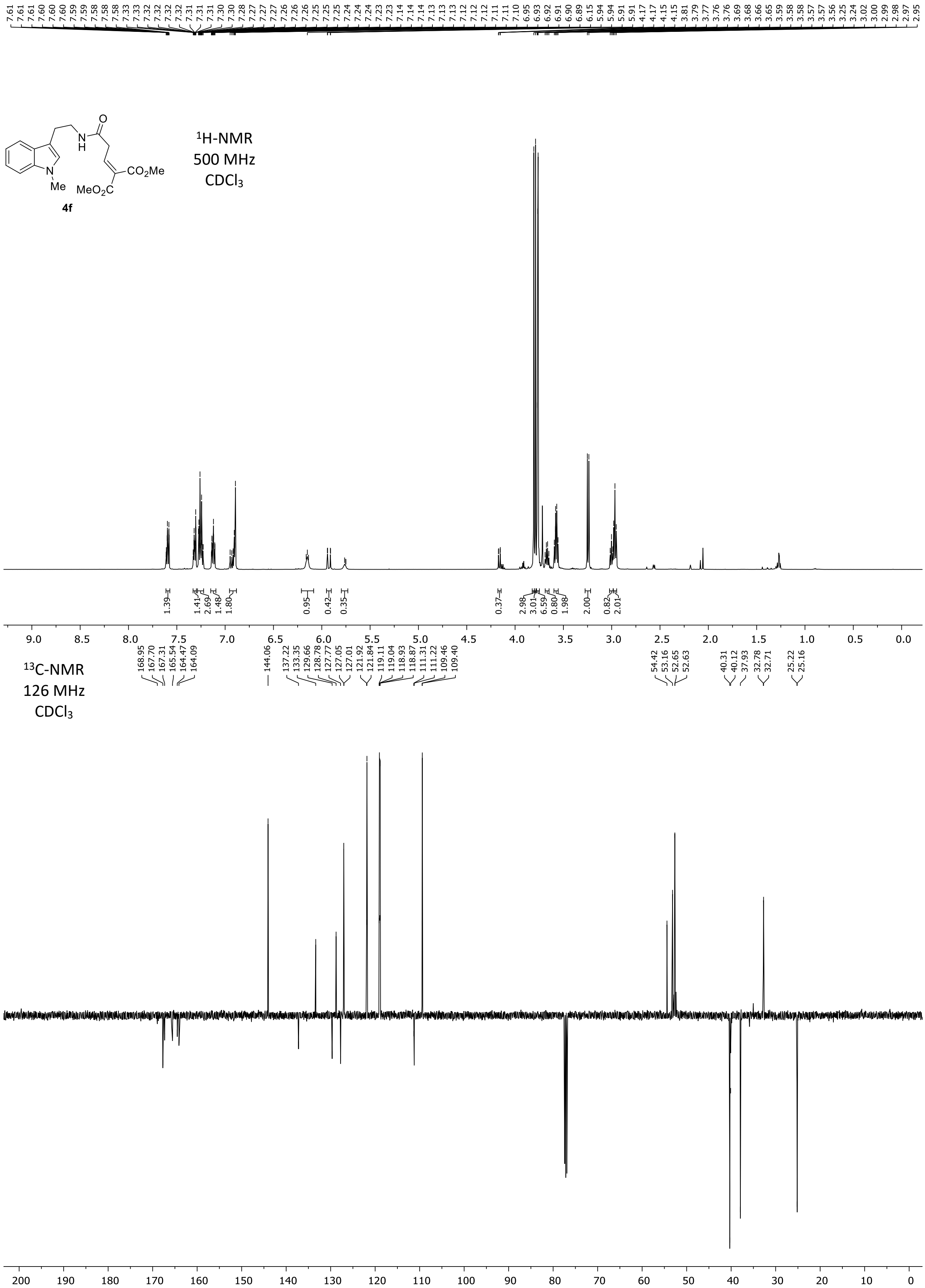
7.61 7.61 7.60 7.60 7.60 7.59 7.59 7.58 7.58 7.58 7.33 7.33 7.32 7.32 7.32 7.32 7.31 7.31 7.31 7.30 7.28 7.27 7.27 7.27 7.26 7.26 7.25 7.25 7.25 7.24 7.24 7.24 7.23 7.23 7.14 7.14 7.13 7.13 7.13 7.12 7.12 7.12 7.11 7.11 7.10 6.95 6.93 6.92 6.91 6.90 6.89 6.15 5.94 5.94 5.91 5.91 4.17 4.15 4.15 3.81 3.79 3.77 3.76 3.76 3.69 3.68 3.66 3.65 3.59 3.58 3.58 3.57 3.56 3.25 3.24 3.02 3.00 2.99 2.98 2.97 2.95
O
N
H
CO2Me
N
Me
MeO2C
4f
1H-NMR
500 MHz
CDCl3
1.39 1.41 2.69 1.48 1.80 0.95 0.42 0.35 0.37 2.98 3.01 6.59 0.80 1.98 2.00 0.82 2.01
9.0 8.5 8.0 7.5 7.0 6.5 6.0 5.5 5.0 4.5 4.0 3.5 3.0 2.5 2.0 1.5 1.0 0.5 0.0
13C-NMR
126 MHz
CDCl3
168.95 167.70 167.31 165.54 164.47 164.09 144.06 137.22 133.35 129.66 128.78 127.77 127.05 127.01 121.92 121.84 119.11 119.04 118.93 118.87 111.31 111.22 109.46 109.40 54.42 53.16 52.65 52.63 40.31 40.12 37.93 32.78 32.71 25.22 25.16
200 190 180 170 160 150 140 130 120 110 100 90 80 70 60 50 40 30 20 10 0

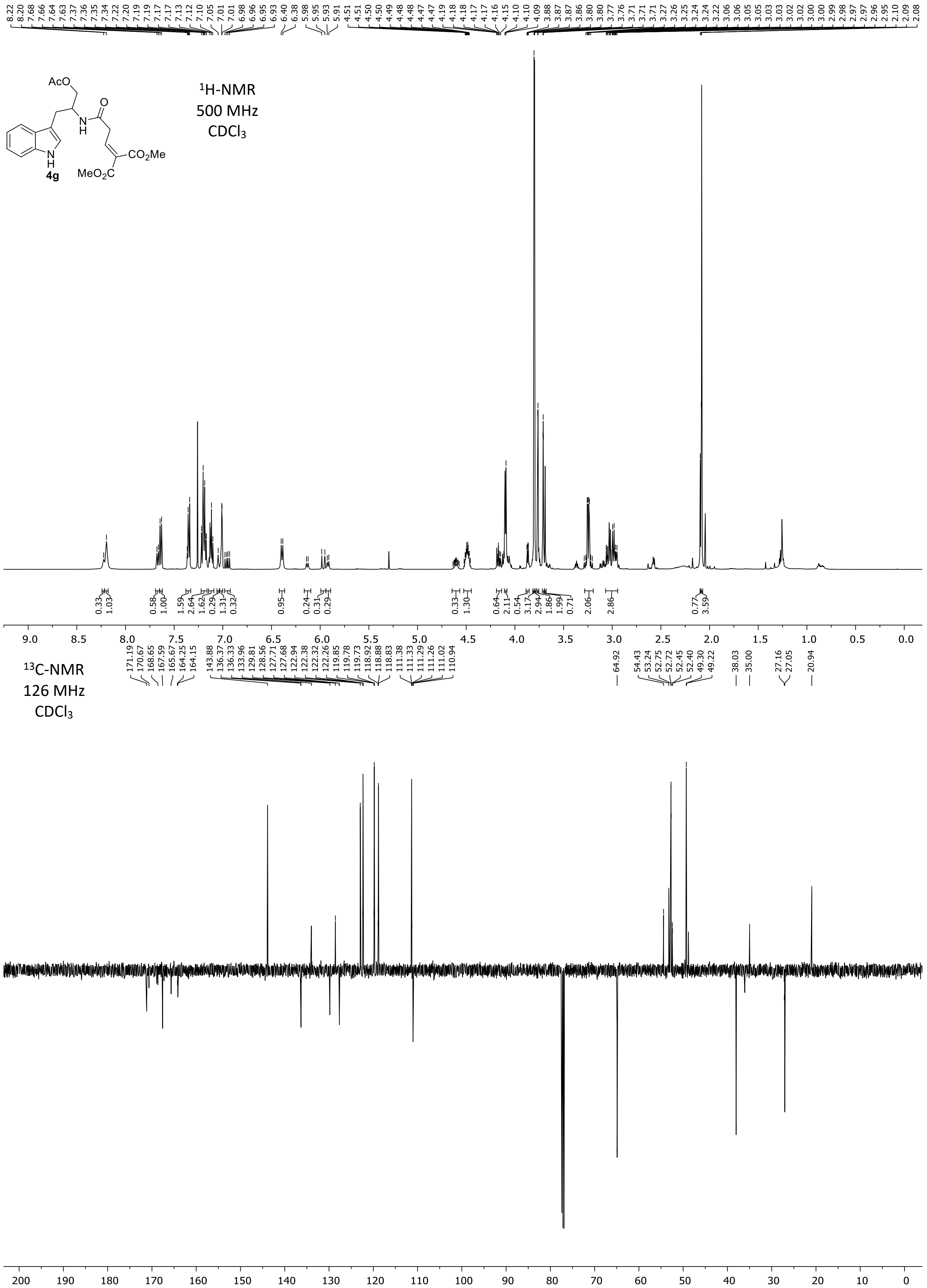
AcO
N
H
O
CO2Me
MeO2C
N
H
4g
1H-NMR
500 MHz
CDCl3
8.22 8.20 7.68 7.66 7.64 7.63 7.37 7.36 7.35 7.34 7.22 7.20 7.19 7.19 7.17 7.17 7.13 7.12 7.10 7.05 7.01 7.01 6.98 6.96 6.95 6.93 6.40 6.38 5.98 5.95 5.93 5.91 4.51 4.51 4.50 4.50 4.49 4.48 4.48 4.47 4.47 4.19 4.18 4.18 4.17 4.17 4.16 4.15 4.10 4.09 3.88 3.87 3.87 3.86 3.80 3.80 3.77 3.76 3.71 3.71 3.71 3.27 3.26 3.25 3.24 3.24 3.22 3.06 3.06 3.05 3.05 3.03 3.03 3.02 3.00 3.00 2.99 2.98 2.97 2.97 2.96 2.95 2.10 2.09 2.08
0.33 1.03 0.58 1.00 1.59 2.64 1.62 0.29 1.31 0.32 0.95 0.24 0.31 0.29 0.33 1.30 0.64 2.11 0.54 3.17 2.94 1.86 1.99 0.71 2.06 2.86 0.77 3.59
9.0 8.5 8.0 7.5 7.0 6.5 6.0 5.5 5.0 4.5 4.0 3.5 3.0 2.5 2.0 1.5 1.0 0.5 0.0
13C-NMR
126 MHz
CDCl3
171.19 170.67 168.65 167.59 165.67 164.25 164.15 143.88 136.37 136.33 133.96 129.81 128.56 127.71 127.68 122.94 122.38 122.32 122.26 119.85 119.78 119.73 118.92 118.88 118.83 111.38 111.33 111.29 111.26 111.02 110.94
64.92
54.43 53.24 52.75 52.72 52.45 52.40 49.30 49.22
38.03 35.00 27.16 27.05 20.94
200 190 180 170 160 150 140 130 120 110 100 90 80 70 60 50 40 30 20 10 0

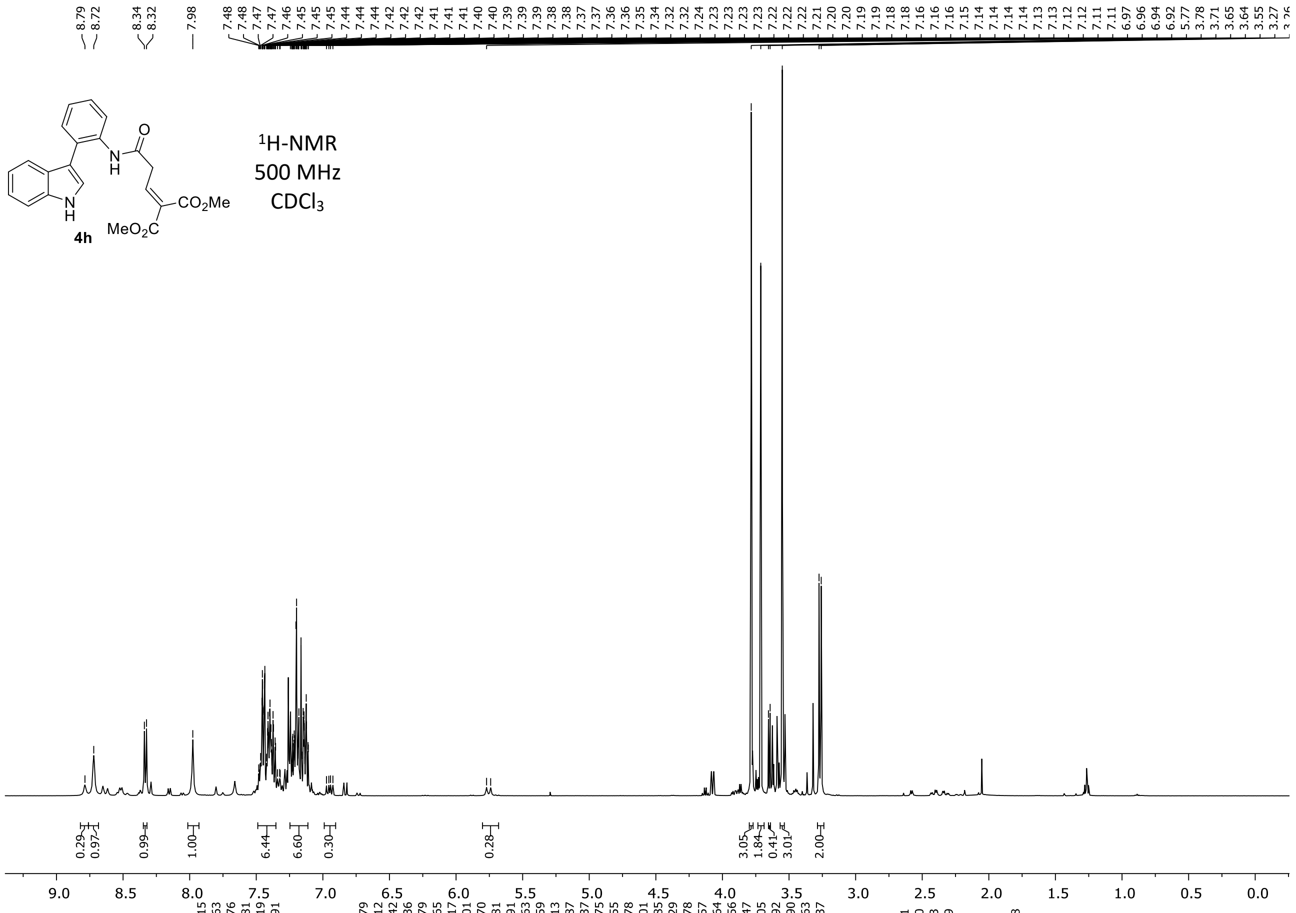

4h
MeO$_2$C
CO$_2$Me
^{1}H-NMR
500 MHz
CDCl$_3$
8.79 8.72 8.34 8.32 7.98 7.48 7.48 7.47 7.47 7.46 7.45 7.45 7.44 7.44 7.44 7.42 7.42 7.42 7.41 7.41 7.41 7.40 7.40 7.39 7.39 7.39 7.38 7.38 7.37 7.37 7.36 7.35 7.34 7.32 7.32 7.24 7.23 7.23 7.23 7.23 7.22 7.22 7.22 7.21 7.20 7.20 7.19 7.19 7.18 7.18 7.16 7.16 7.15 7.15 7.14 7.14 7.14 7.14 7.13 7.13 7.12 7.12 7.11 7.11 6.97 6.96 6.94 6.92 5.77 3.78 3.71 3.65 3.64 3.55 3.27
0.29 0.97 0.99 1.00 6.44 6.60 0.30 0.28 3.05 1.84 0.41 3.01 2.00
9.0 8.5 8.0 7.5 7.0 6.5 6.0 5.5 5.0 4.5 4.0 3.5 3.0 2.5 2.0 1.5 1.0 0.5 0.0

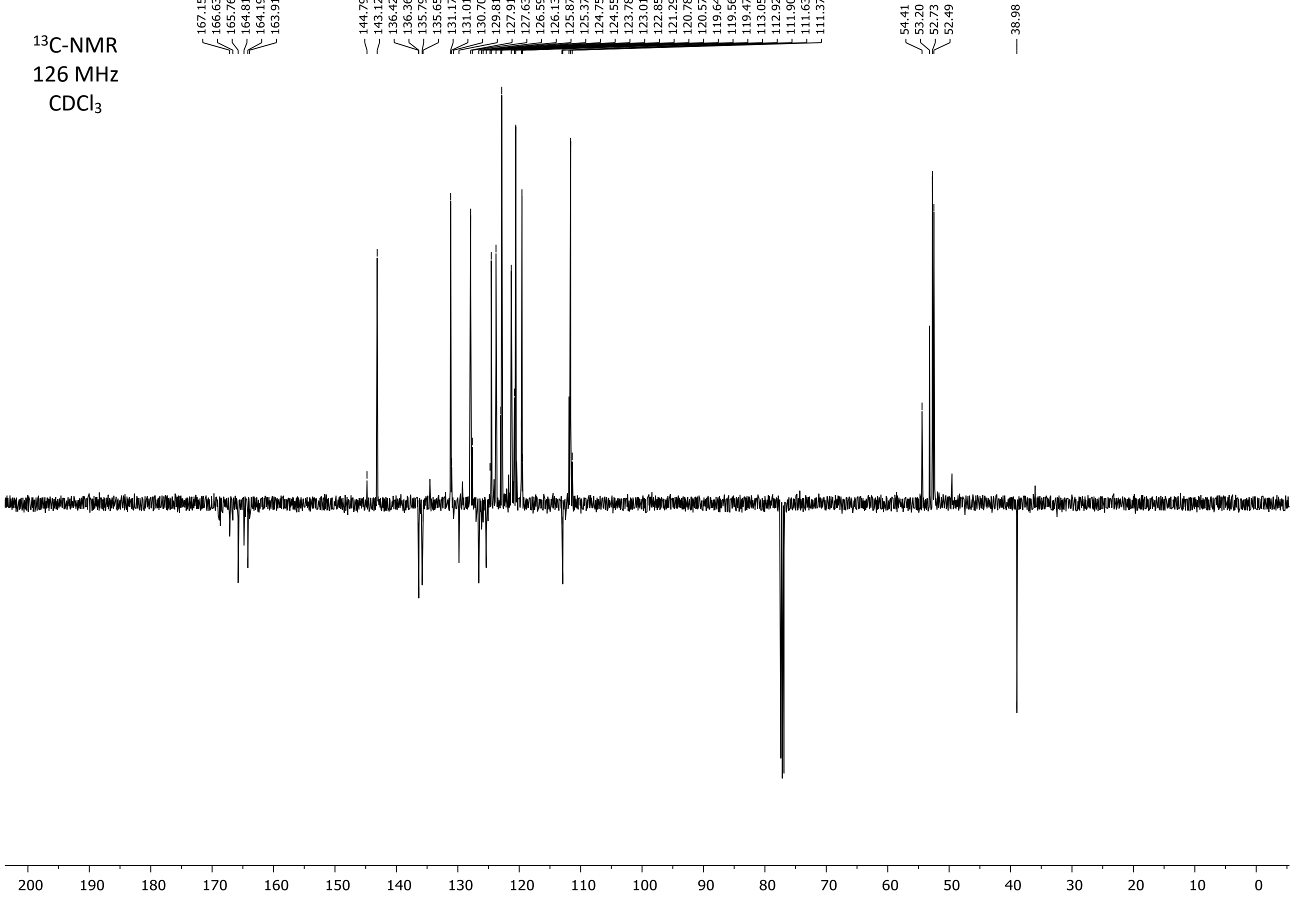

^{13}C-NMR
126 MHz
CDCl$_3$
167.15 166.63 165.76 164.81 164.19 163.91 144.79 143.12 136.42 136.36 135.79 135.65 131.17 131.01 130.70 129.81 127.91 127.63 126.59 126.13 125.87 125.37 124.75 124.55 123.78 123.01 122.85 121.29 120.78 120.57 119.64 119.56 119.47 113.05 112.92 111.90 111.63 111.37 54.41 53.20 52.73 52.49 38.98
200 190 180 170 160 150 140 130 120 110 100 90 80 70 60 50 40 30 20 10 0

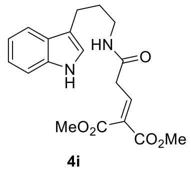

4i

^{1}H-NMR
500 MHz
$CDCl_3$

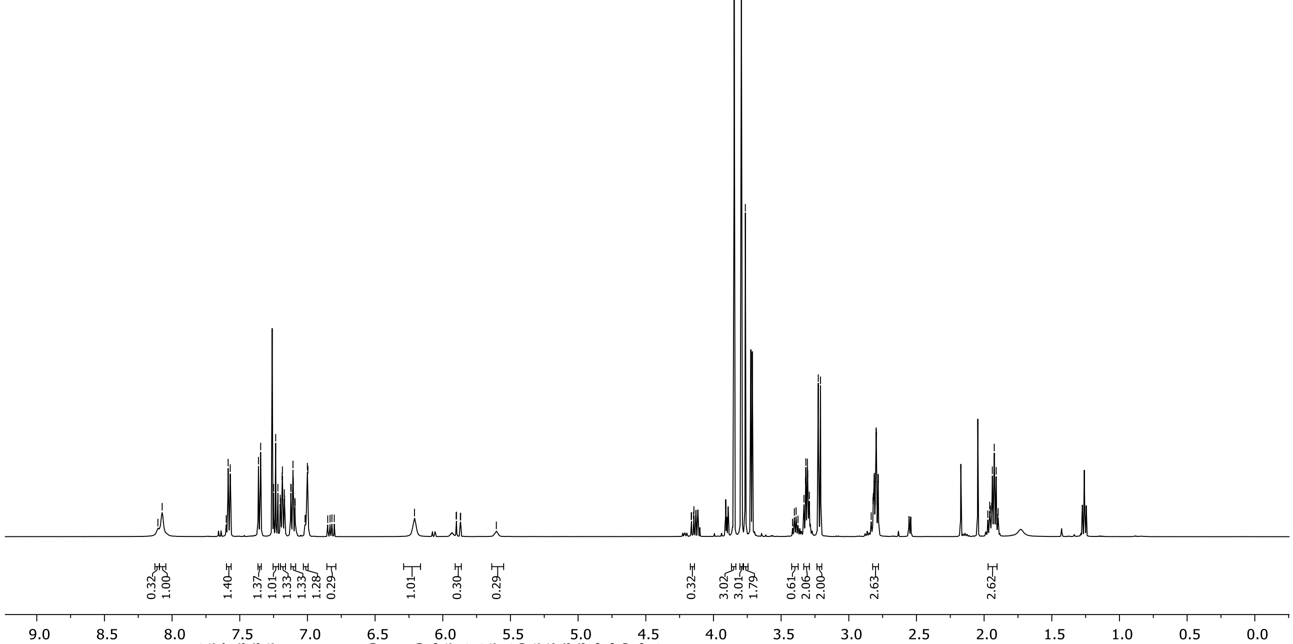

^{13}C-NMR
126 MHz
$CDCl_3$

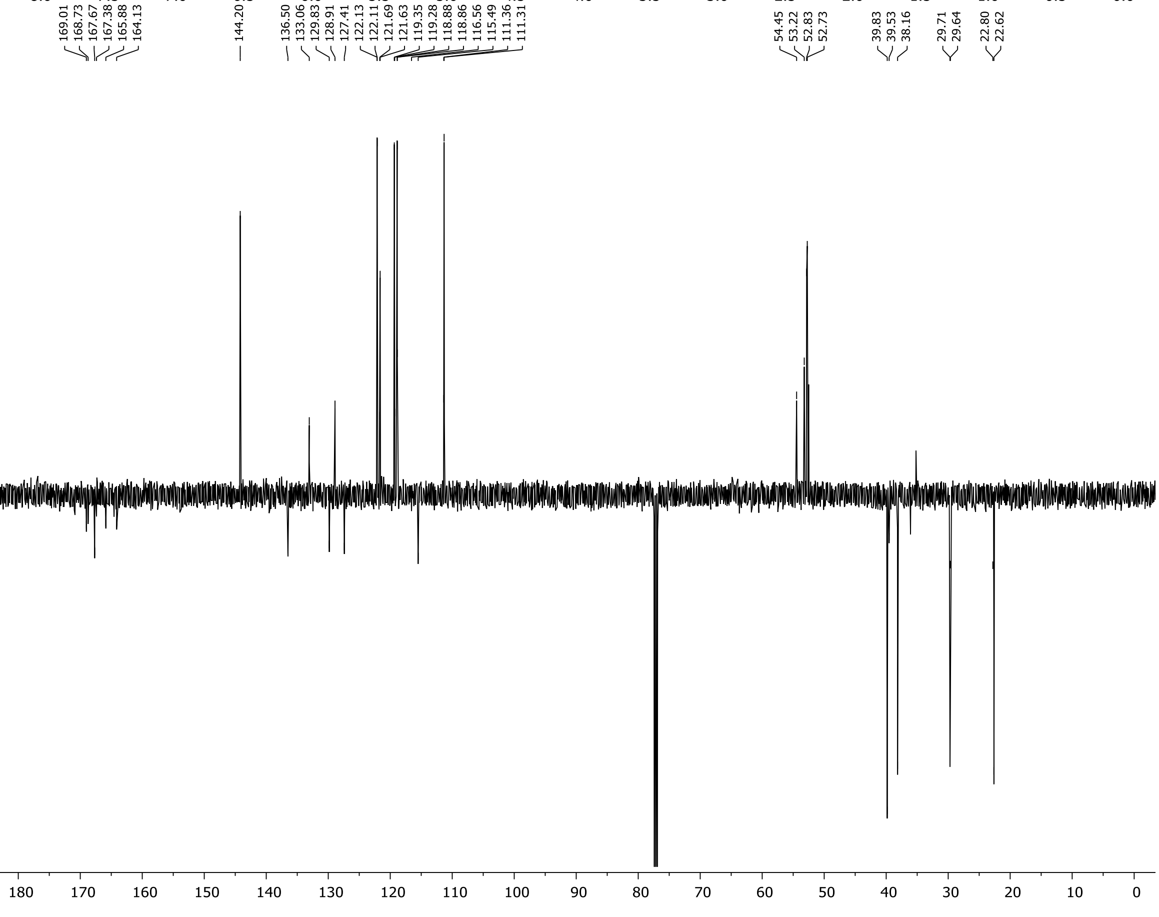

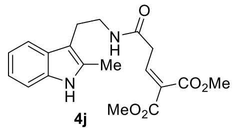

4j

^{1}H-NMR
600 MHz
$CDCl_3$

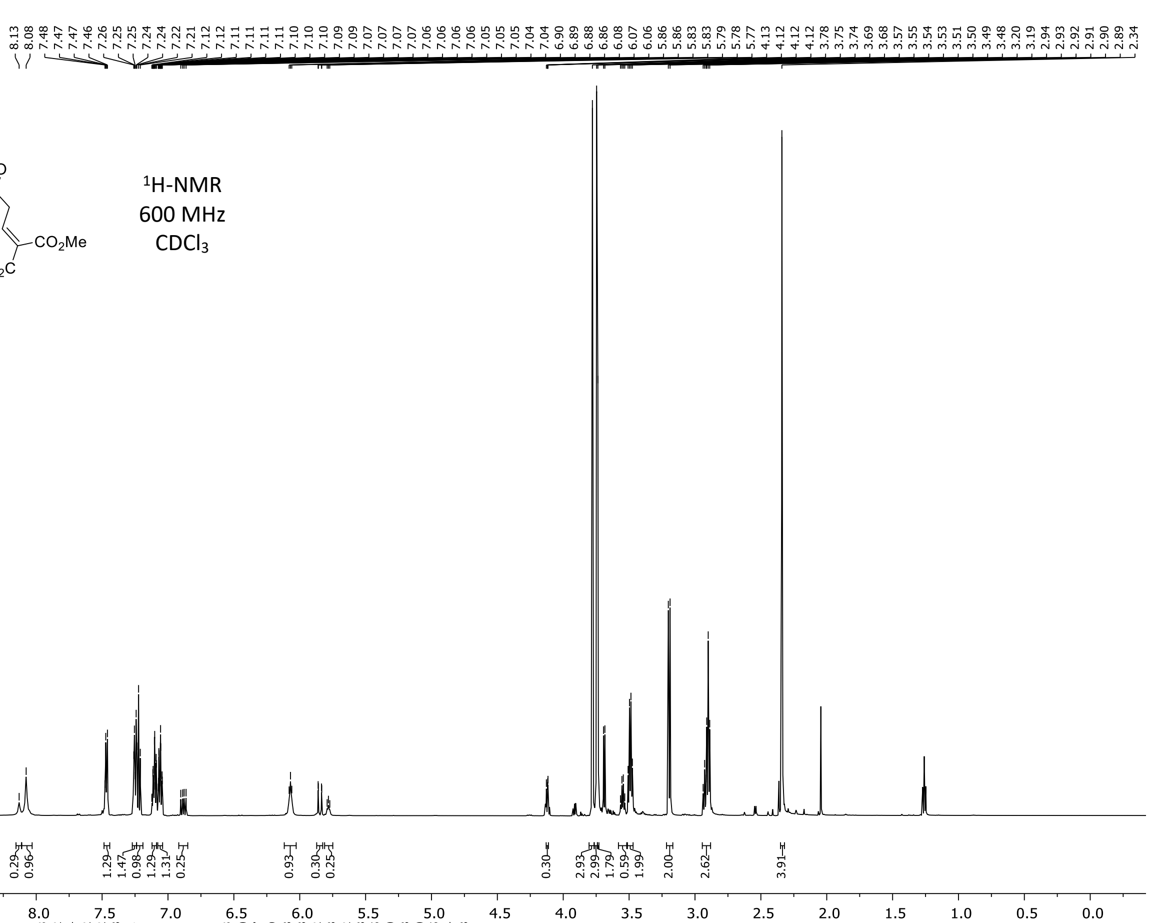

^{13}C-NMR
151 MHz
$CDCl_3$

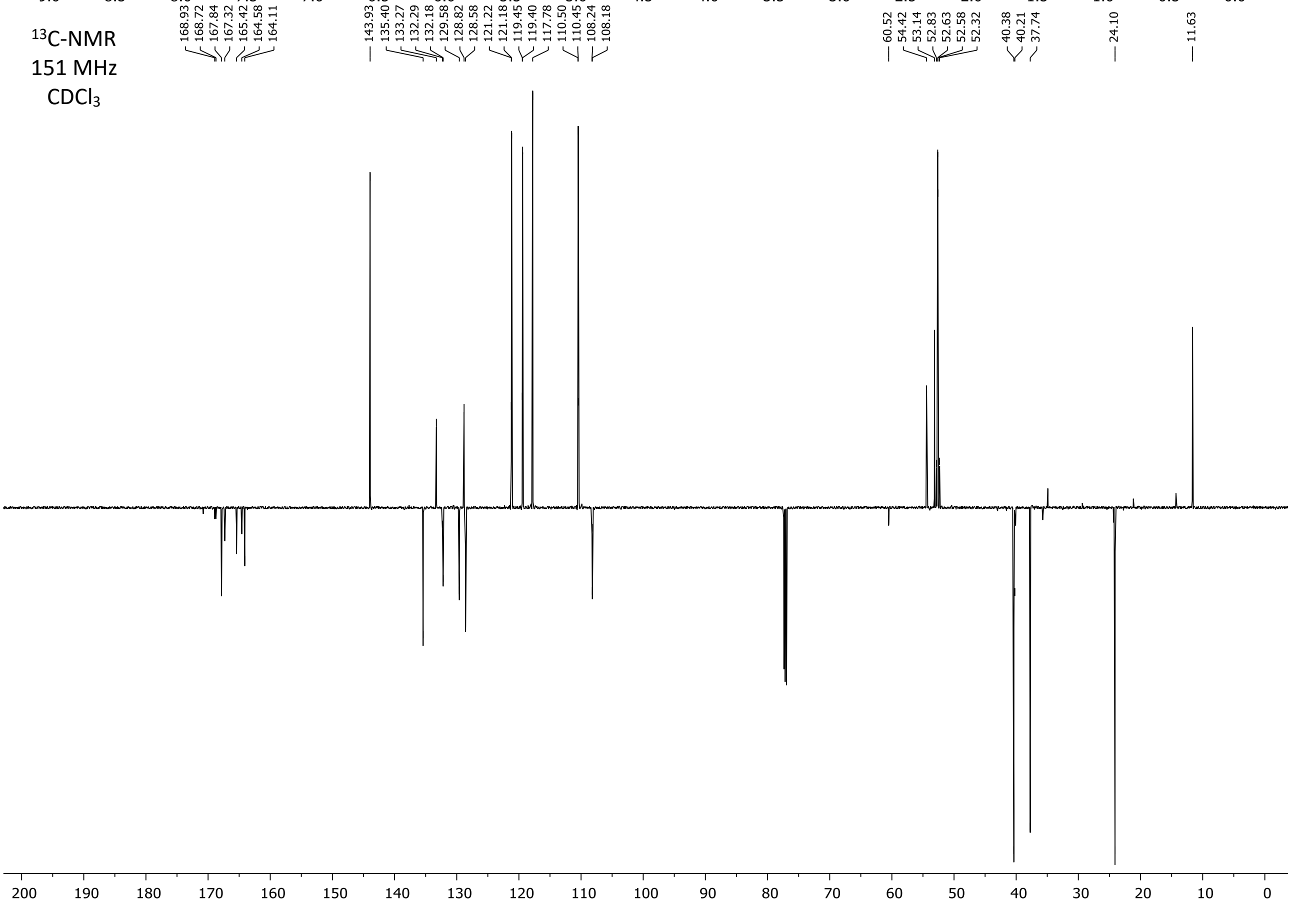

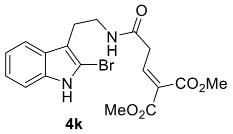

4k

^{1}H-NMR
600 MHz
$CDCl_3$

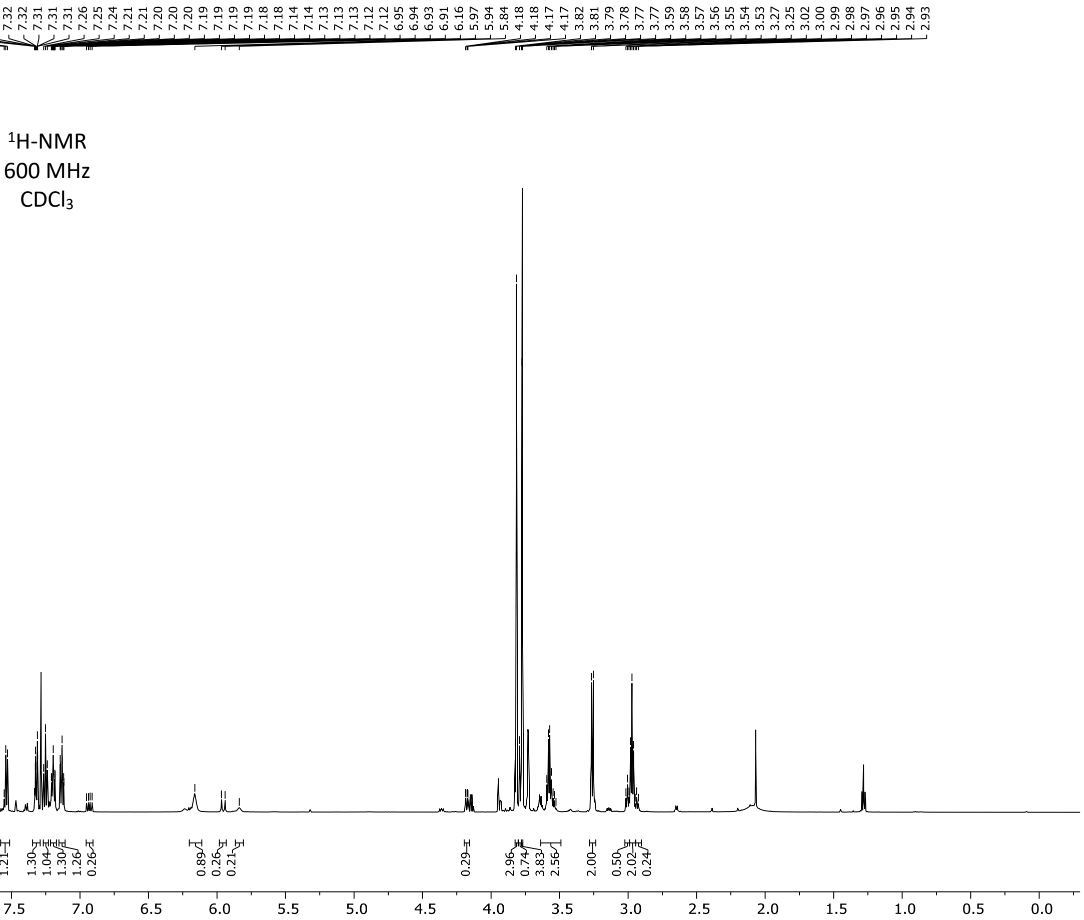

^{13}C-NMR
151 MHz
$CDCl_3$

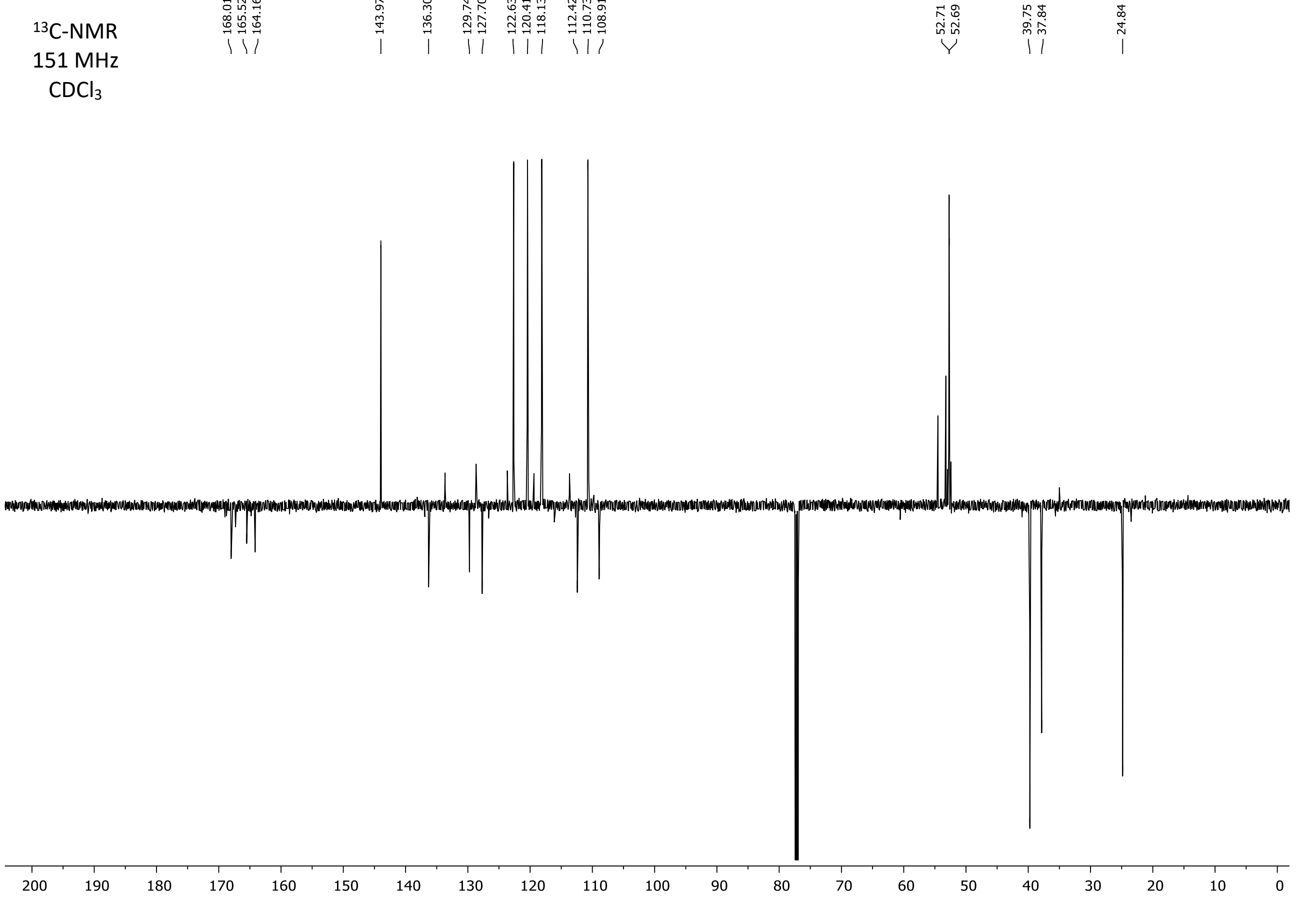

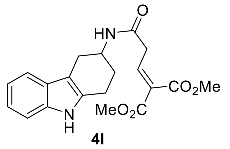

4l

^{1}H-NMR
500 MHz
$CDCl_3$

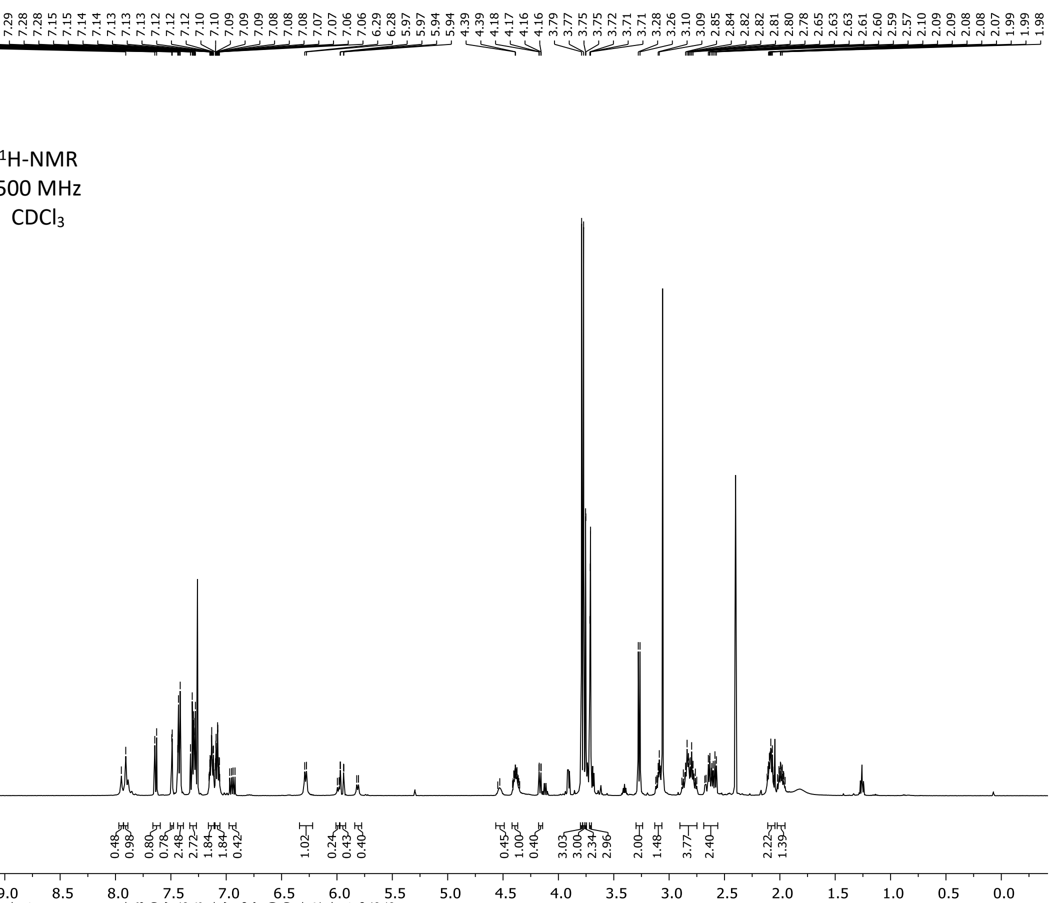

^{13}C-NMR
126 MHz
$CDCl_3$

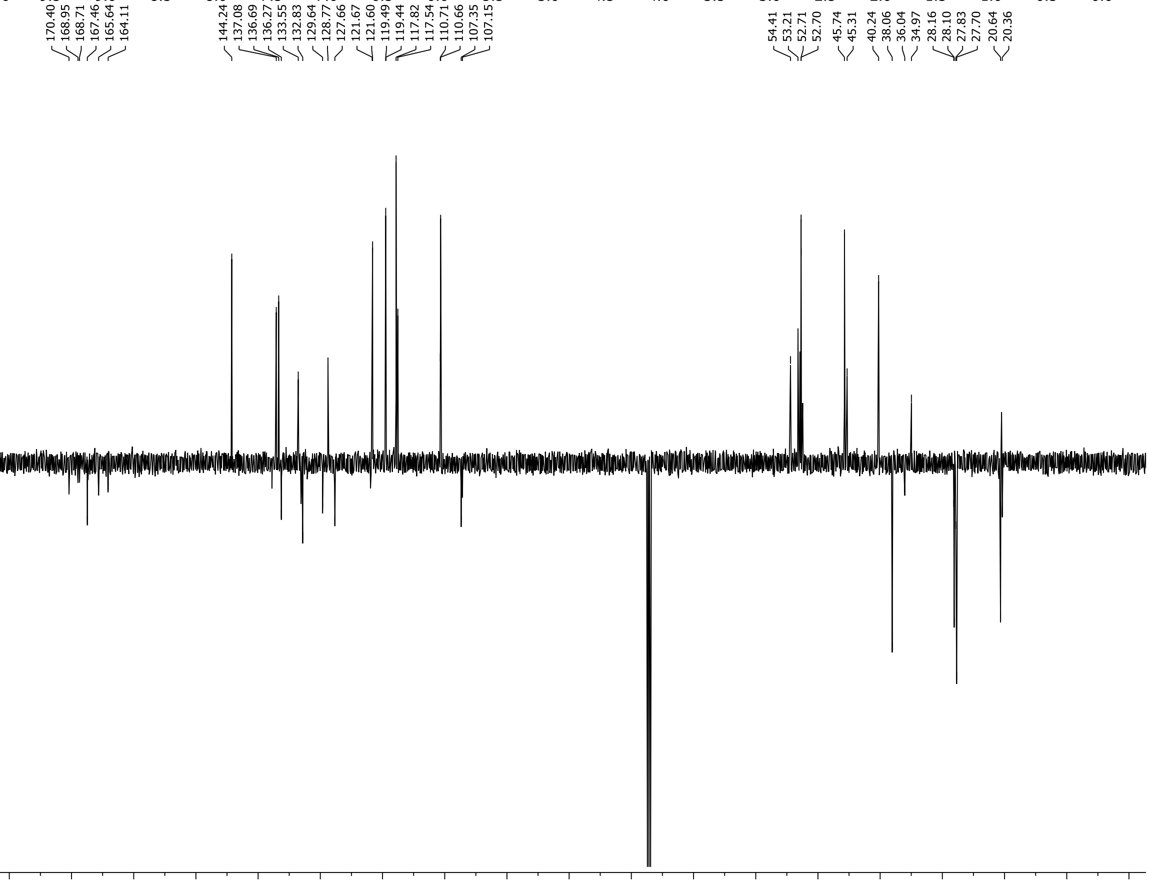

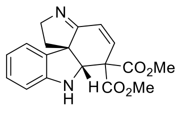

7a

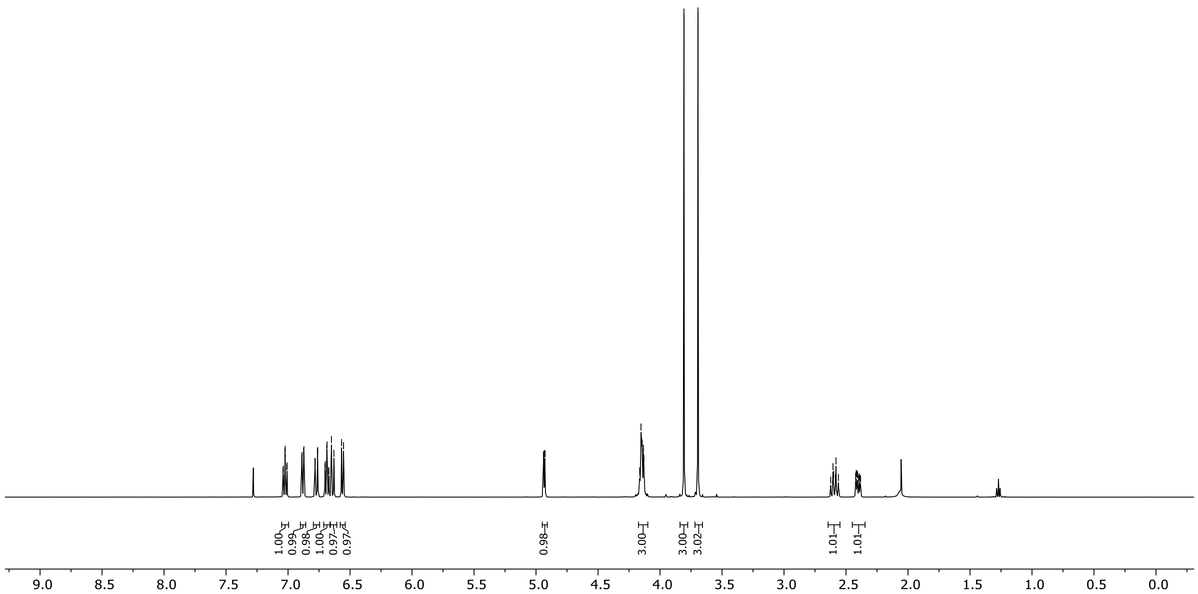

^{1}H-NMR
500 MHz
$CDCl_3$

^{13}C-NMR
126 MHz
$CDCl_3$

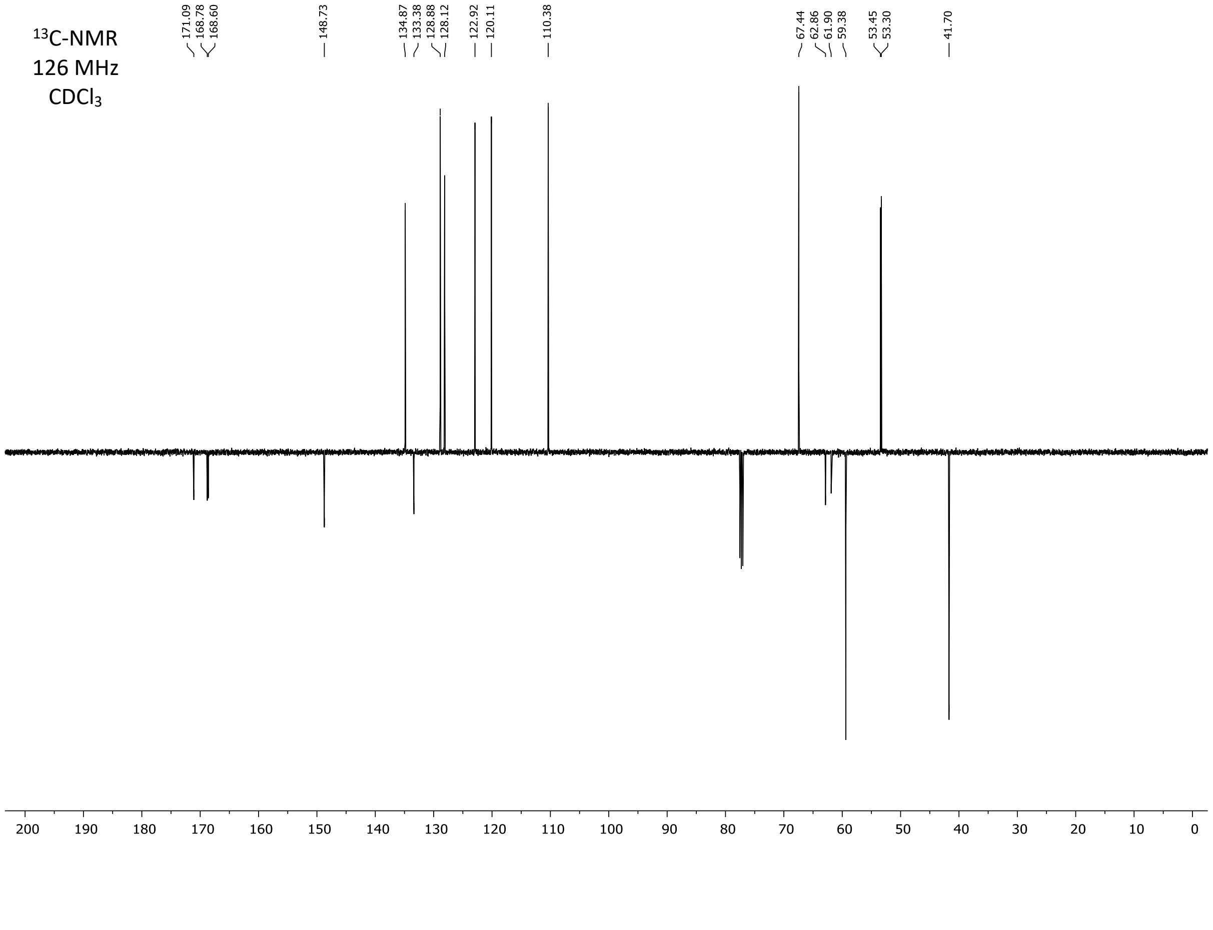

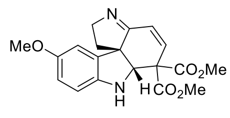

7b

^{1}H-NMR
500 MHz
$CDCl_3$

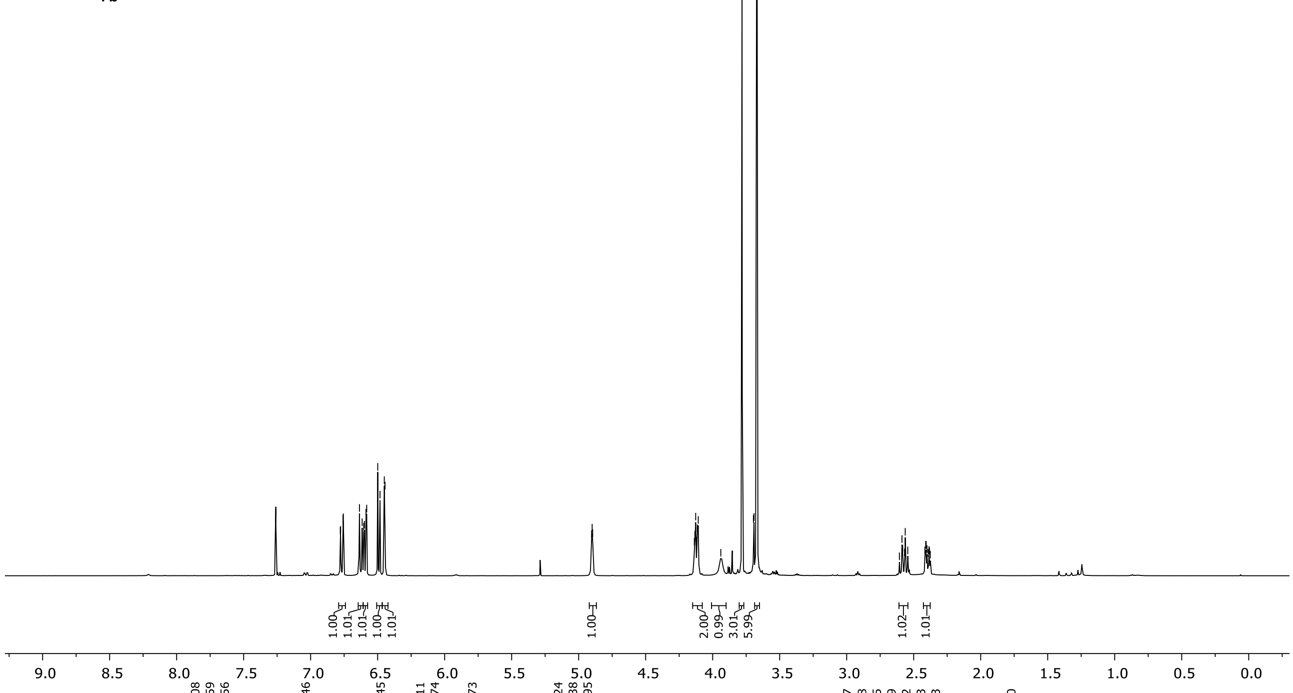

^{13}C-NMR
126 MHz
$CDCl_3$

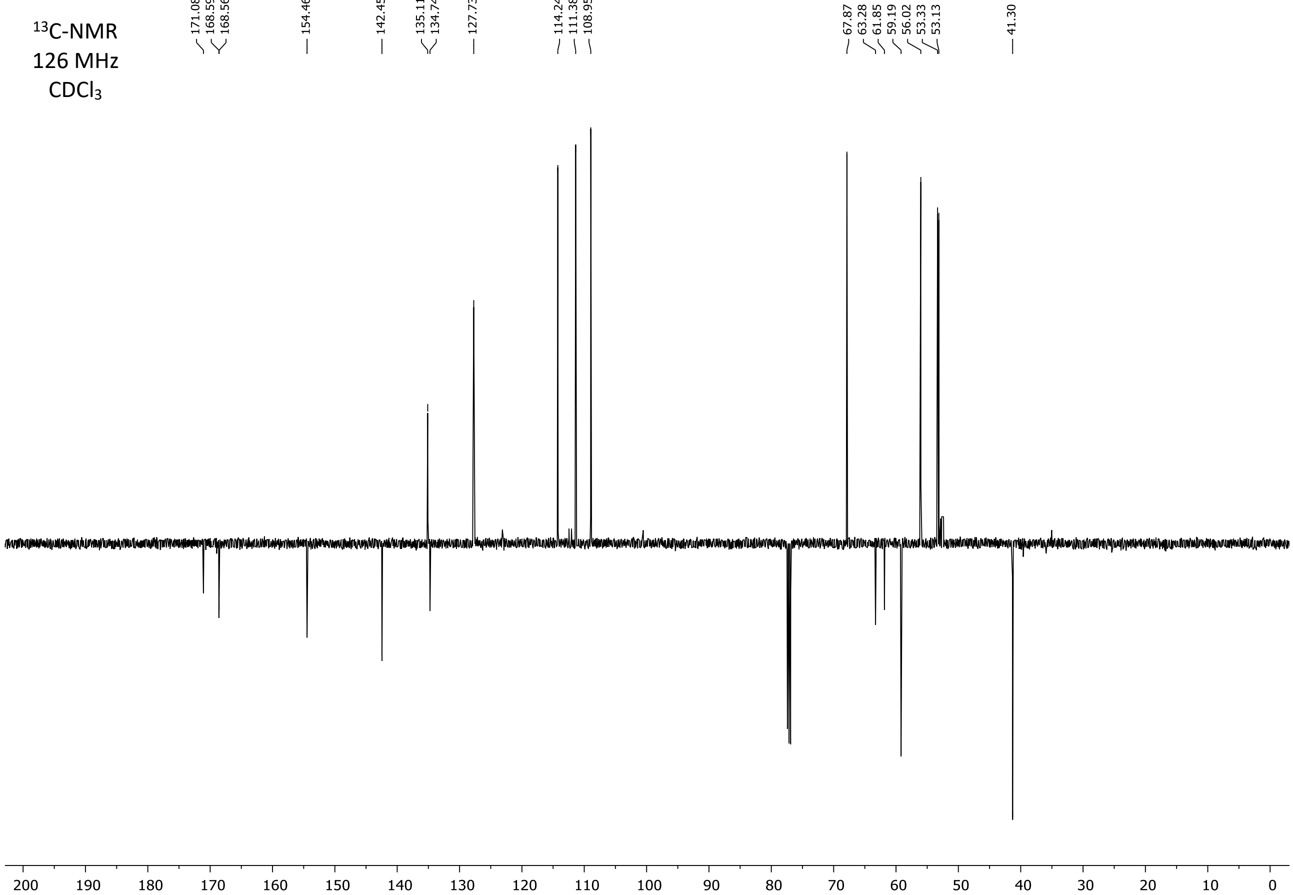

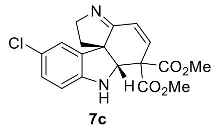

7c

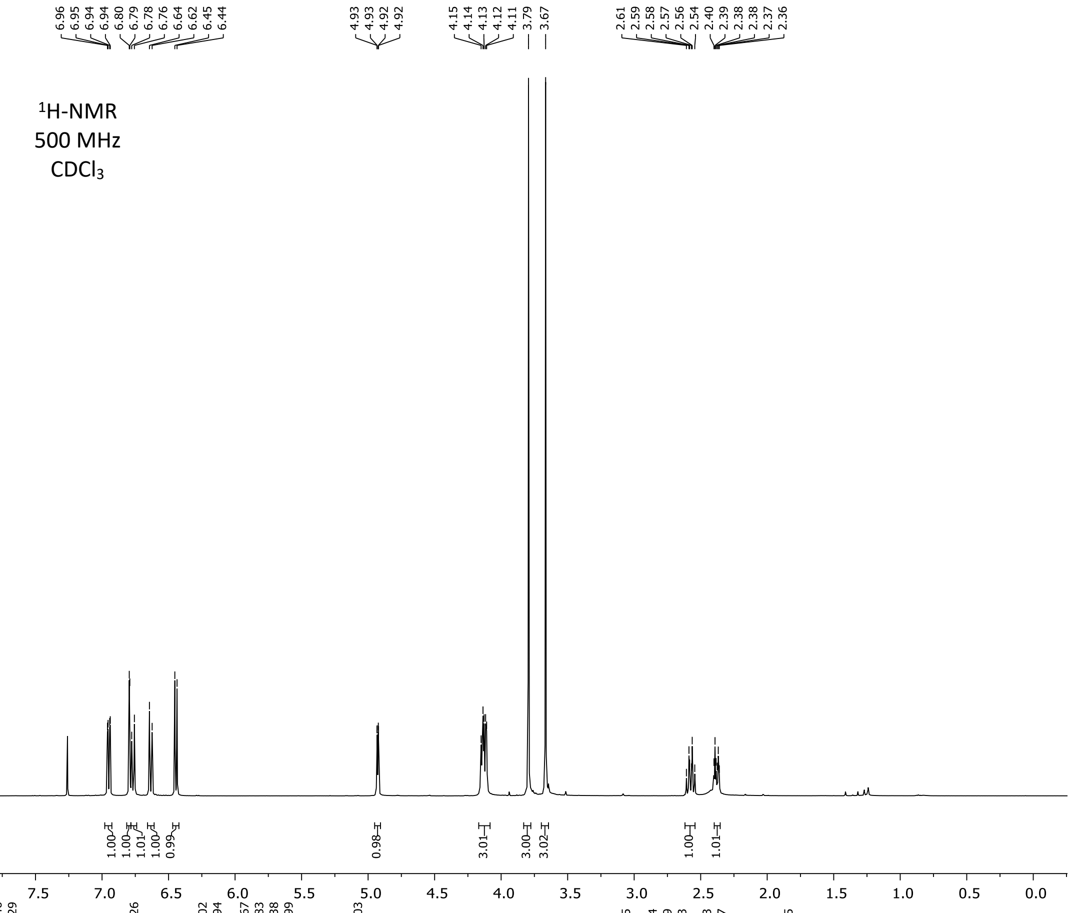

1H-NMR
500 MHz
CDCl3
6.96
6.95
6.94
6.94
6.80
6.79
6.78
6.76
6.64
6.62
6.45
6.44
4.93
4.93
4.92
4.92
4.15
4.14
4.13
4.12
4.11
3.79
3.67
2.61
2.59
2.58
2.57
2.56
2.54
2.40
2.39
2.38
2.38
2.37
2.36
1.00
1.00
1.01
1.00
0.99
0.98
3.01
3.00
3.02
1.00
1.01
9.0
8.5
8.0
7.5
7.0
6.5
6.0
5.5
5.0
4.5
4.0
3.5
3.0
2.5
2.0
1.5
1.0
0.5
0.0

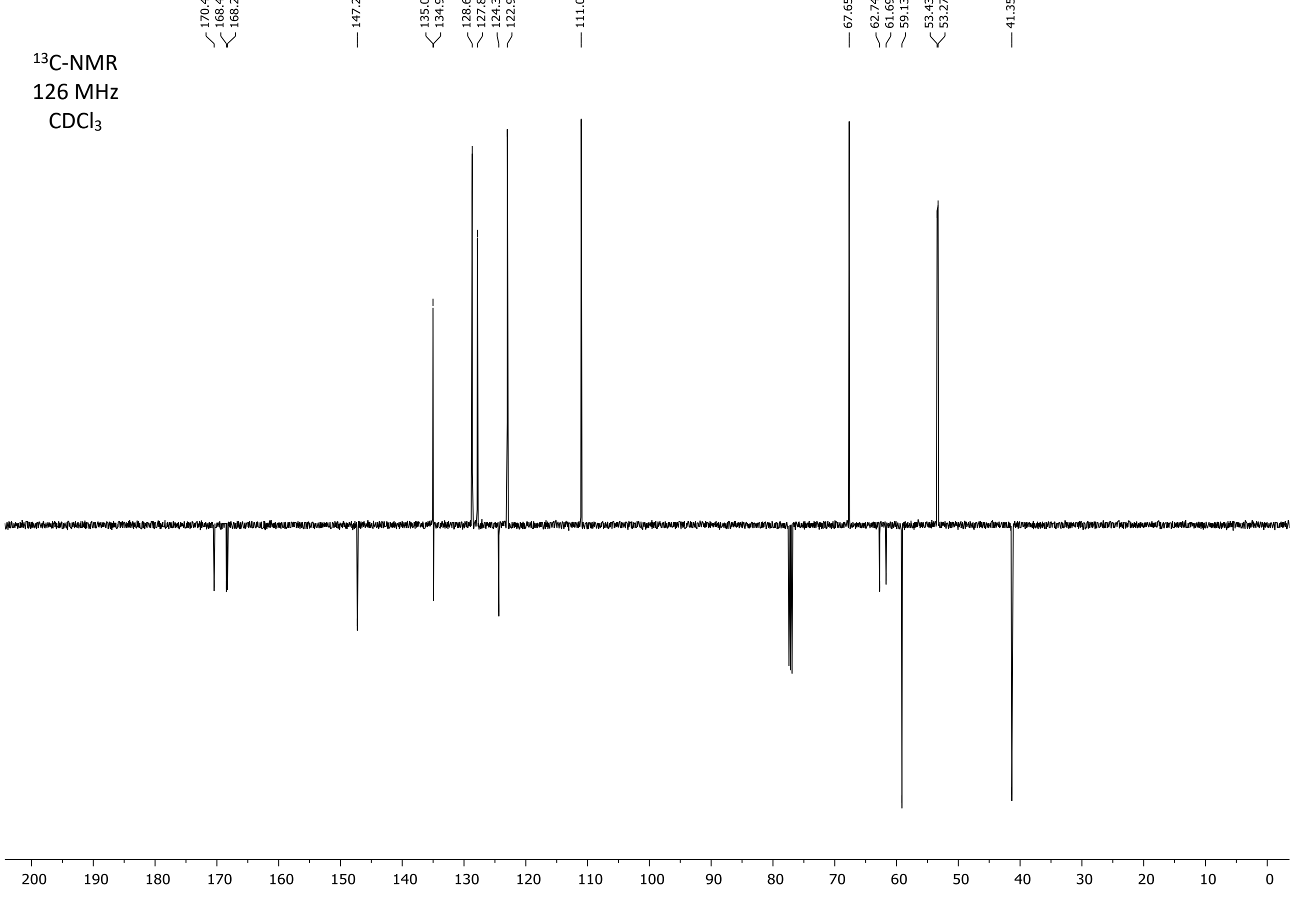

13C-NMR
126 MHz
CDCl3
170.44
168.46
168.29
147.26
135.02
134.94
128.67
127.83
124.38
122.99
111.03
67.65
62.74
61.69
59.13
53.43
53.27
41.35
200
190
180
170
160
150
140
130
120
110
100
90
80
70
60
50
40
30
20
10
0

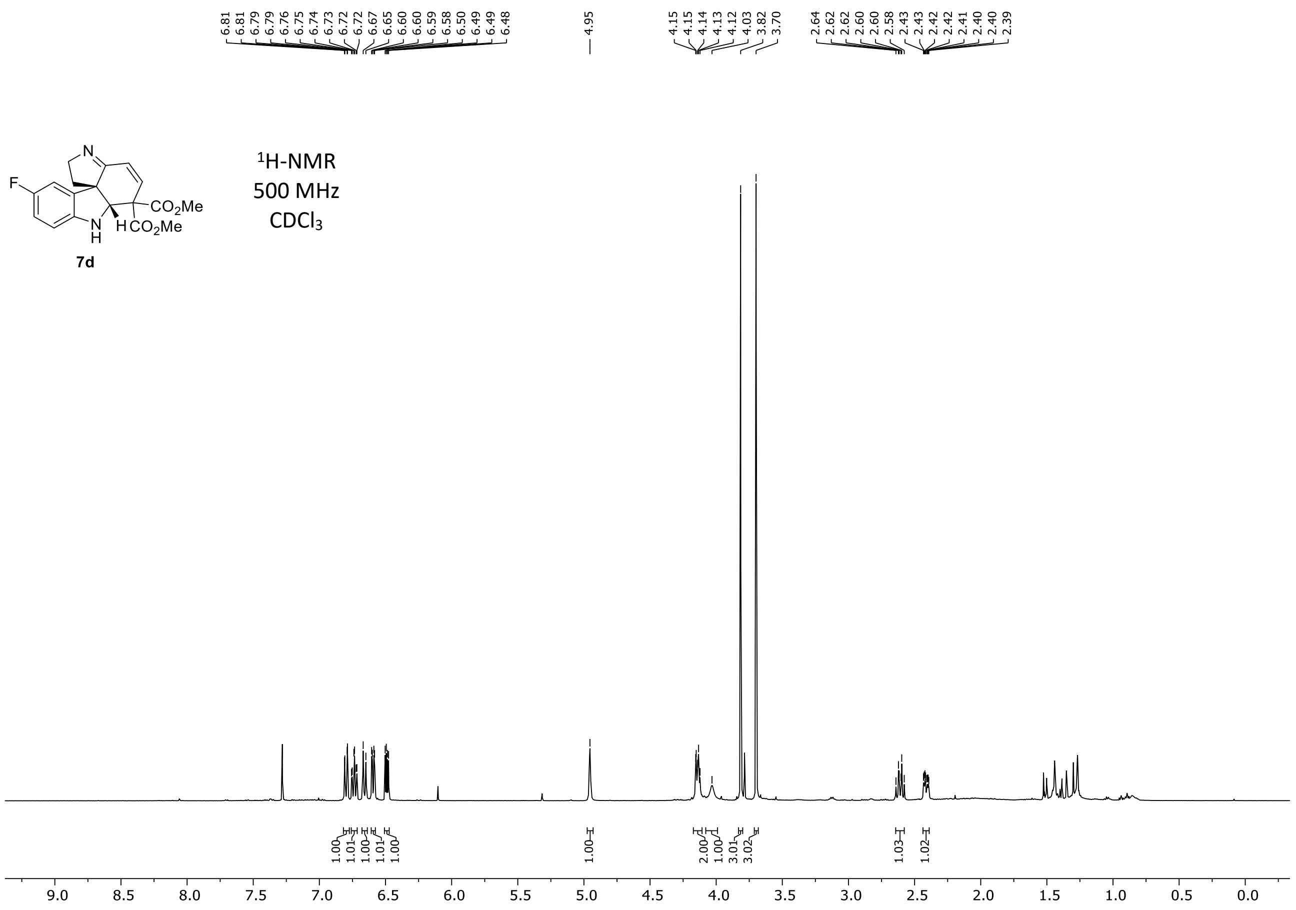
6.81 6.81 6.79 6.79 6.76 6.75 6.74 6.73 6.72 6.72 6.67 6.65 6.60 6.60 6.59 6.58 6.50 6.49 6.49 6.48
4.95
4.15 4.15 4.14 4.13 4.12 4.03 3.82 3.70
2.64 2.62 2.62 2.60 2.60 2.58 2.43 2.43 2.42 2.42 2.41 2.40 2.40 2.39
F
N
CO2Me
H CO2Me
N
H
7d
1H-NMR
500 MHz
CDCl3
1.00 1.01 1.00 1.00 1.01 1.00
1.00
2.00 1.00 3.01 3.02
1.03
1.02
9.0 8.5 8.0 7.5 7.0 6.5 6.0 5.5 5.0 4.5 4.0 3.5 3.0 2.5 2.0 1.5 1.0 0.5 0.0

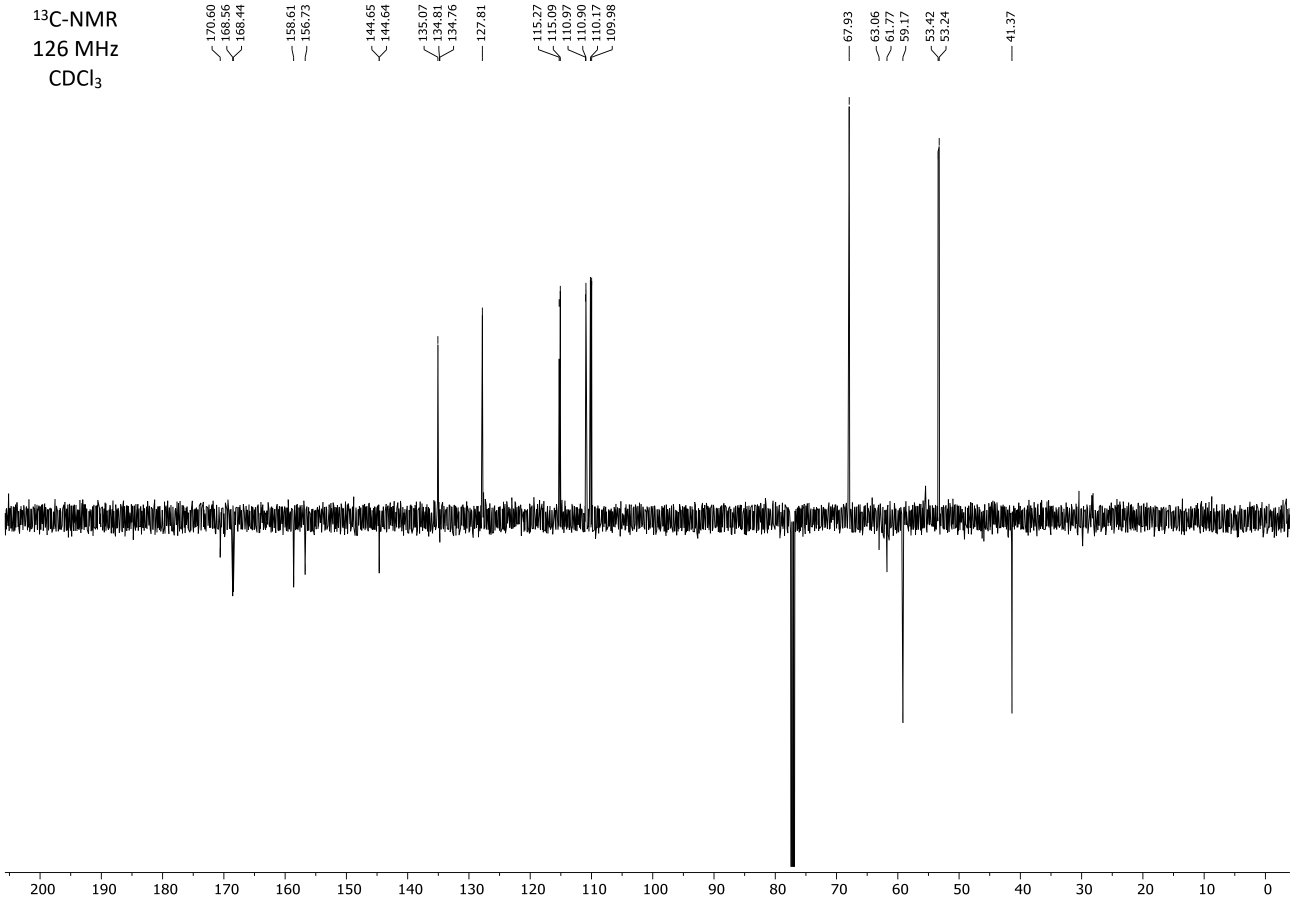
13C-NMR
126 MHz
CDCl3
170.60 168.56 168.44
158.61 156.73
144.65 144.64
135.07 134.81 134.76
127.81
115.27 115.09 110.97 110.90 110.17 109.98
67.93
63.06 61.77 59.17
53.42 53.24
41.37
200 190 180 170 160 150 140 130 120 110 100 90 80 70 60 50 40 30 20 10 0

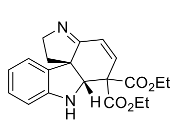
N
CO2Et
N
H
H CO2Et
7e

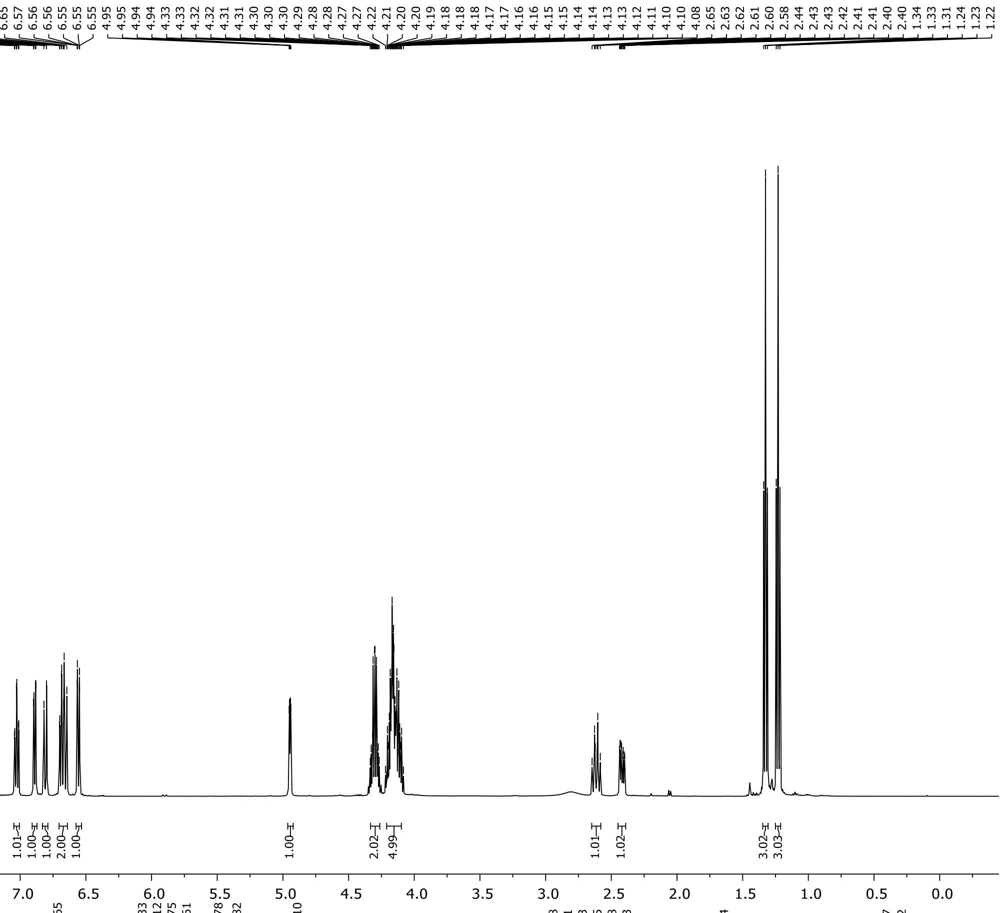
7.04 7.04 7.03 7.03 7.01 7.01 6.90 6.90 6.88 6.88 6.82 6.80 6.70 6.70 6.69 6.68 6.67 6.67 6.67 6.65 6.57 6.56 6.56 6.55 6.55 6.55 4.95 4.95 4.94 4.33 4.33 4.32 4.32 4.31 4.31 4.30 4.30 4.29 4.28 4.28 4.27 4.27 4.22 4.21 4.21 4.20 4.20 4.19 4.18 4.18 4.18 4.17 4.17 4.16 4.16 4.15 4.15 4.14 4.14 4.13 4.13 4.12 4.11 4.10 4.10 4.08 2.65 2.63 2.62 2.61 2.60 2.58 2.44 2.43 2.42 2.41 2.41 2.40 2.40 1.34 1.33 1.31 1.24 1.23 1.22
1H-NMR
500 MHz
CDCl3
1.01 1.00 1.00 2.00 1.00 1.00 2.02 4.99 1.01 1.02 3.02 3.03
9.0 8.5 8.0 7.5 7.0 6.5 6.0 5.5 5.0 4.5 4.0 3.5 3.0 2.5 2.0 1.5 1.0 0.5 0.0

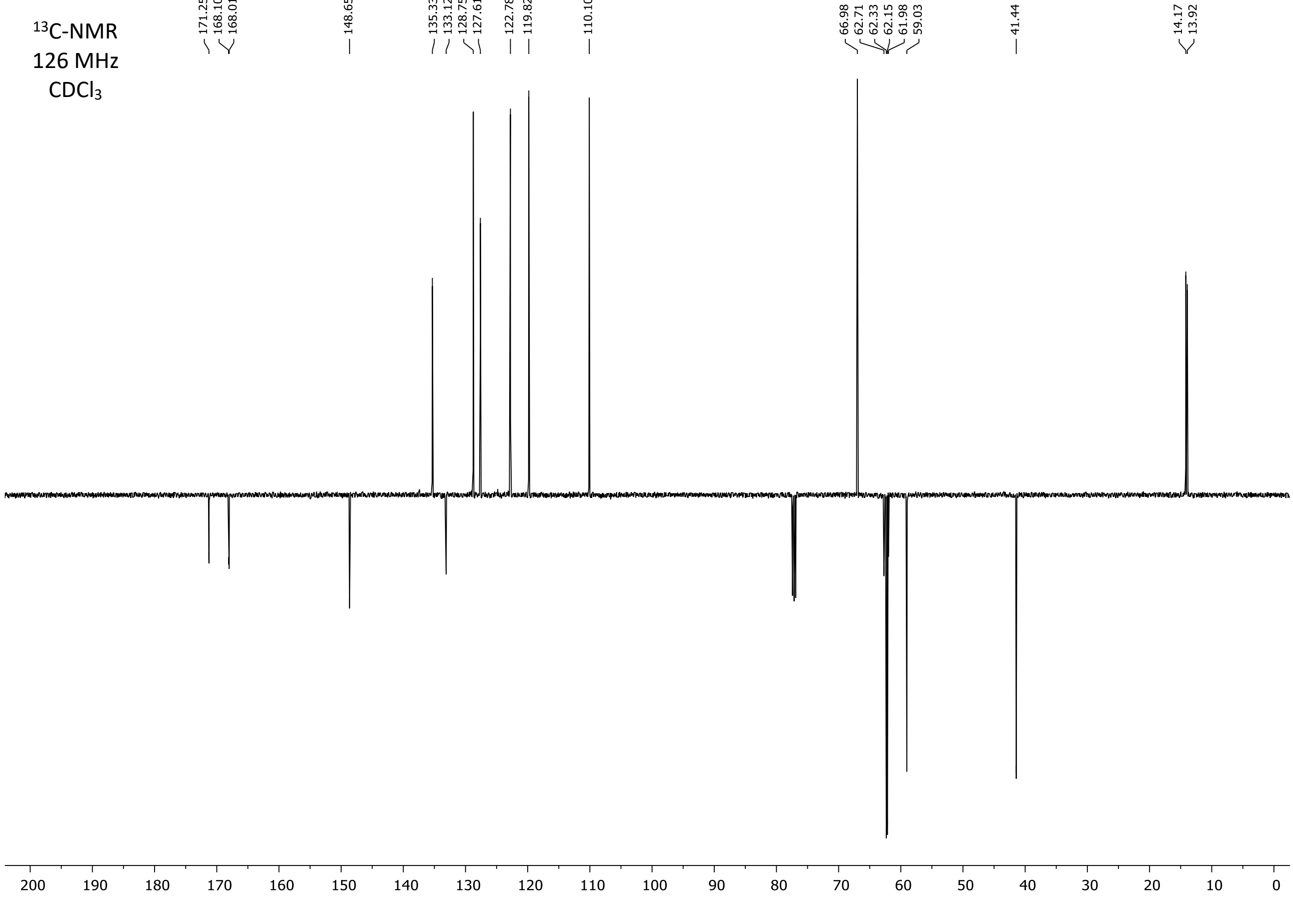
13C-NMR
126 MHz
CDCl3
171.25 168.10 168.01 148.65 135.33 133.12 128.75 127.61 122.78 119.82 110.10 66.98 62.71 62.33 62.15 61.98 59.03 41.44 14.17 13.92
200 190 180 170 160 150 140 130 120 110 100 90 80 70 60 50 40 30 20 10 0

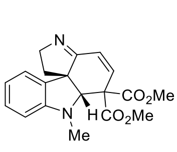

7f

^{1}H-NMR
500 MHz
$CDCl_3$

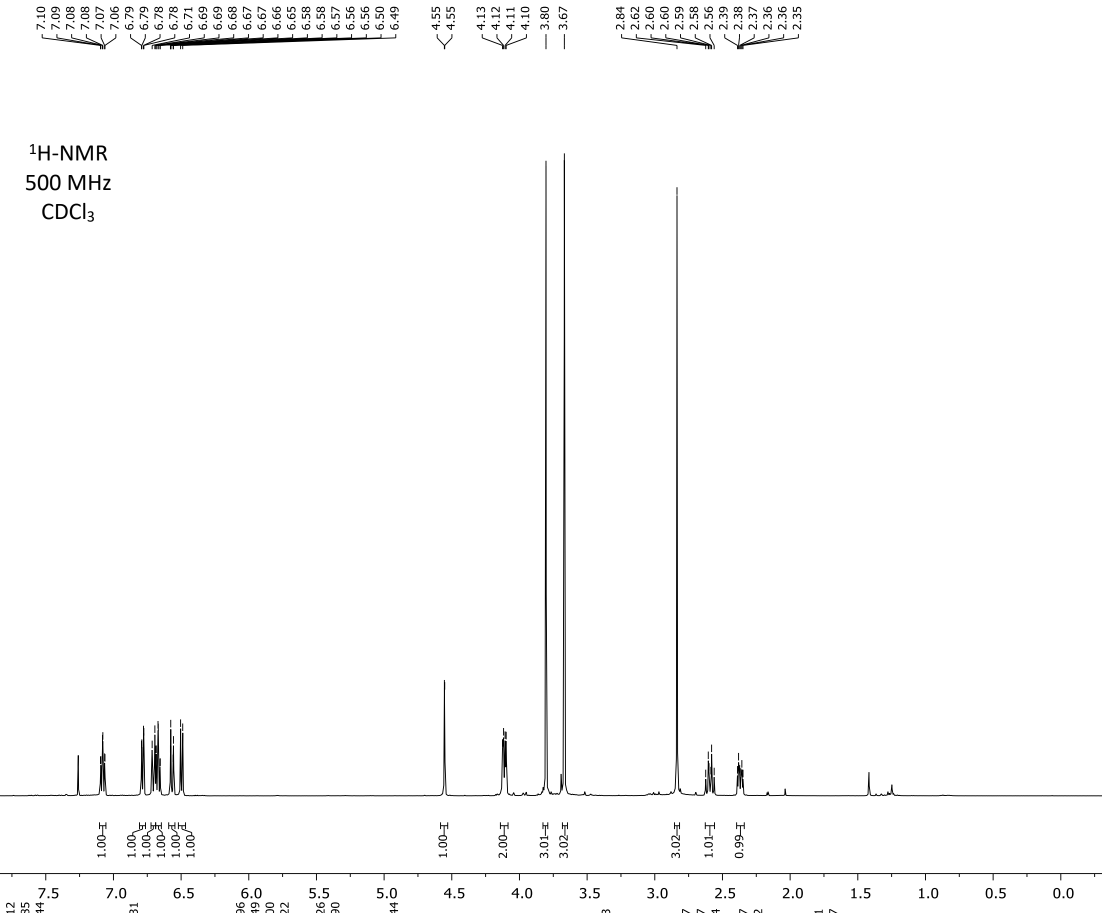

^{13}C-NMR
126 MHz
$CDCl_3$

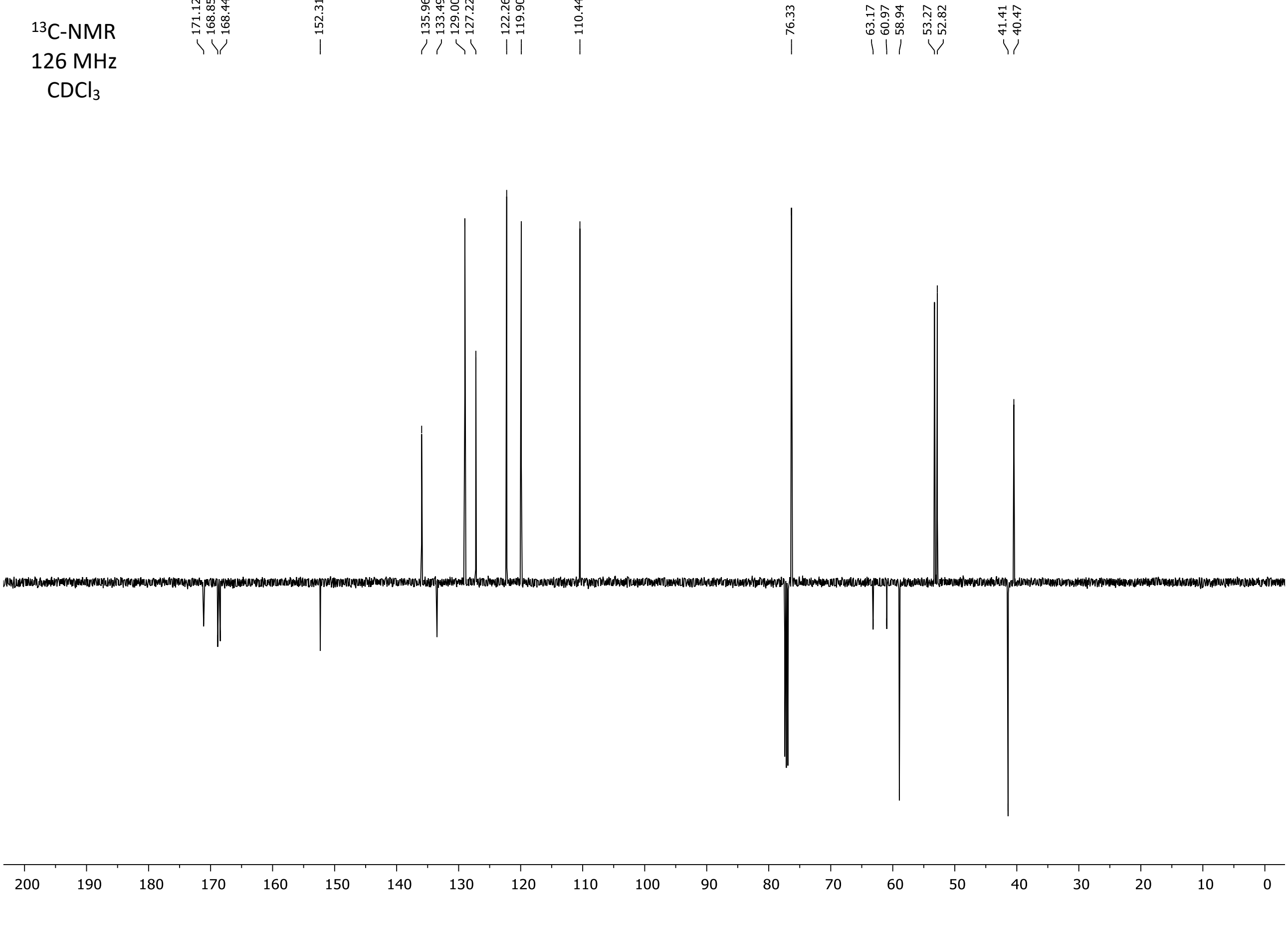

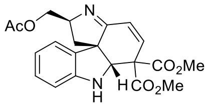

7g

^{1}H-NMR
500 MHz
$CDCl_3$

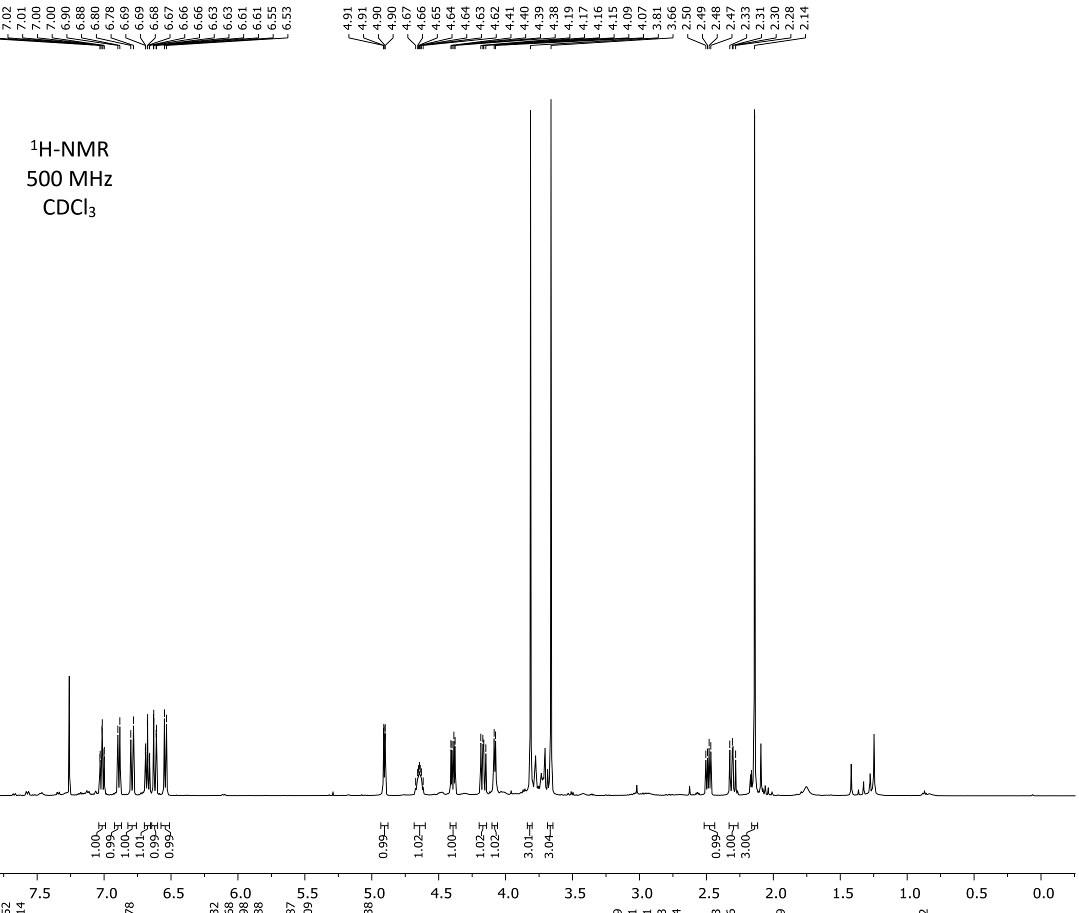

^{13}C-NMR
126 MHz
$CDCl_3$

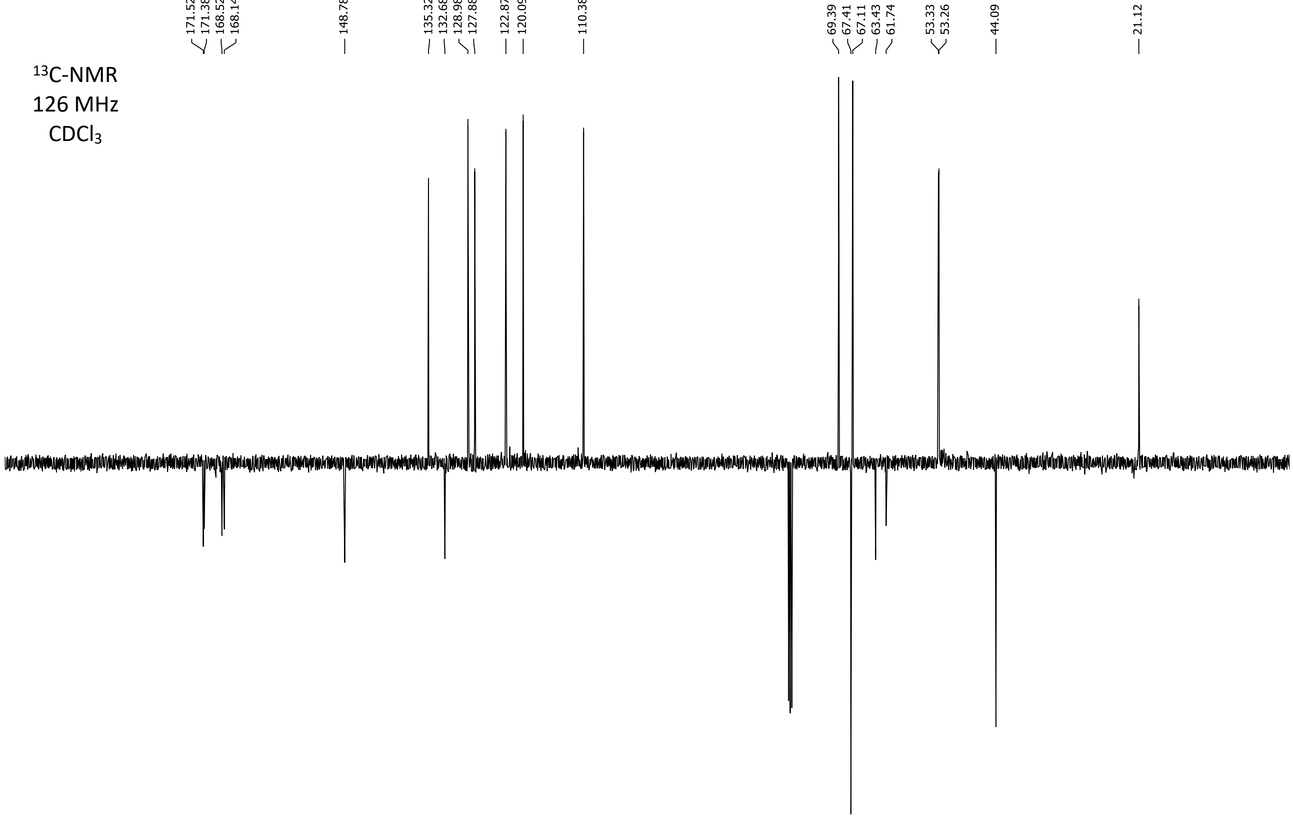

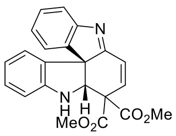

7h

^{1}H-NMR
500 MHz
$CDCl_3$

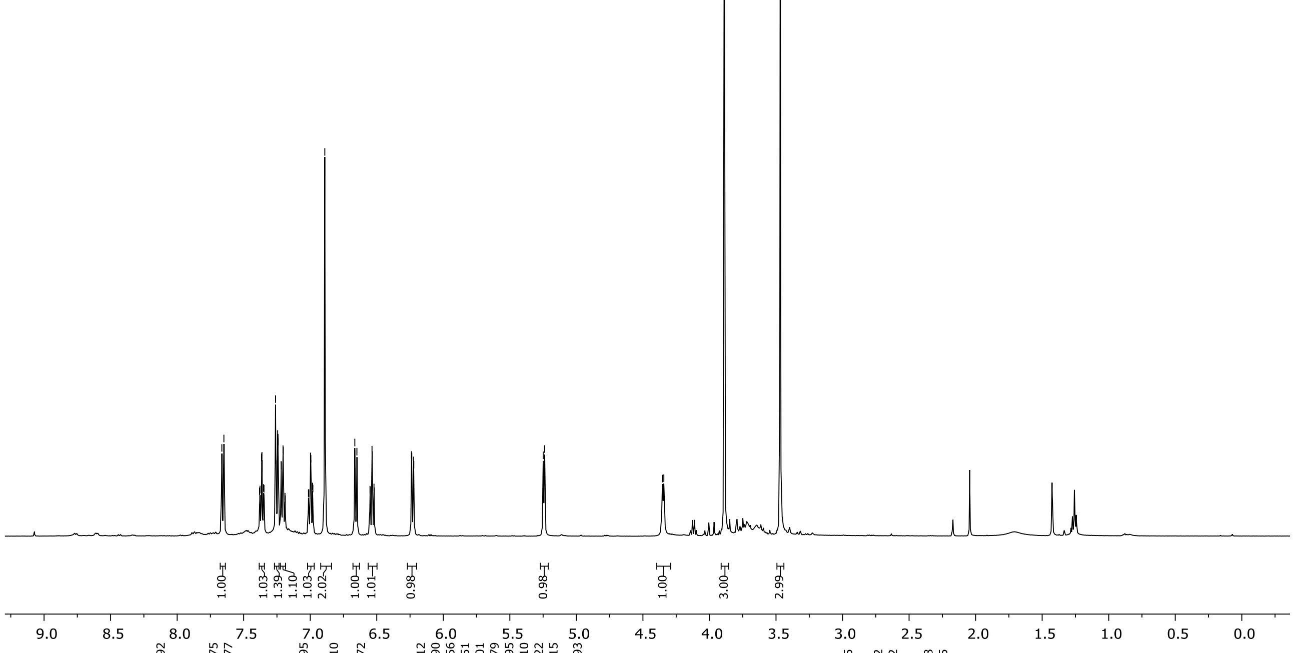

^{13}C-NMR
126 MHz
$CDCl_3$

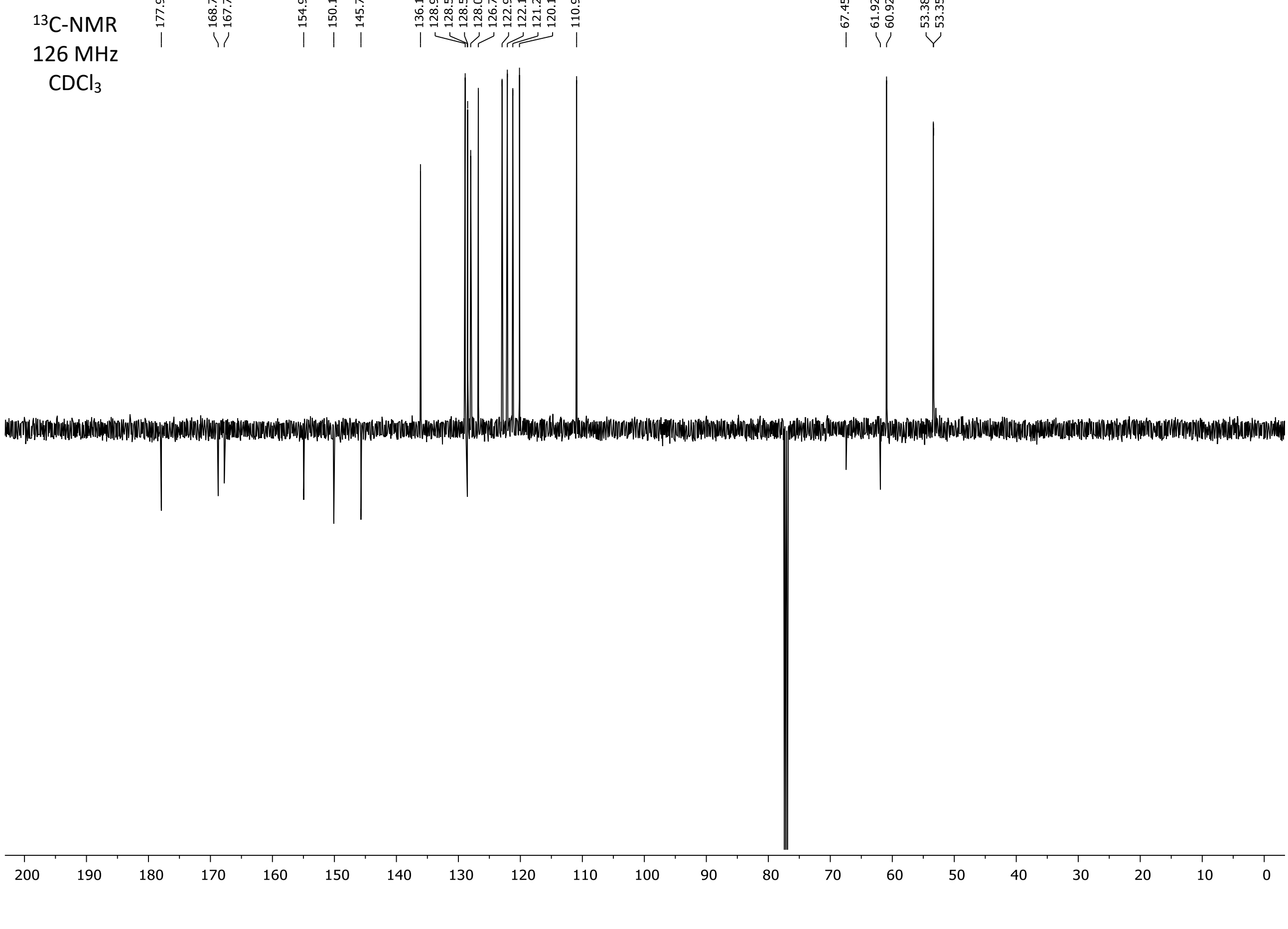

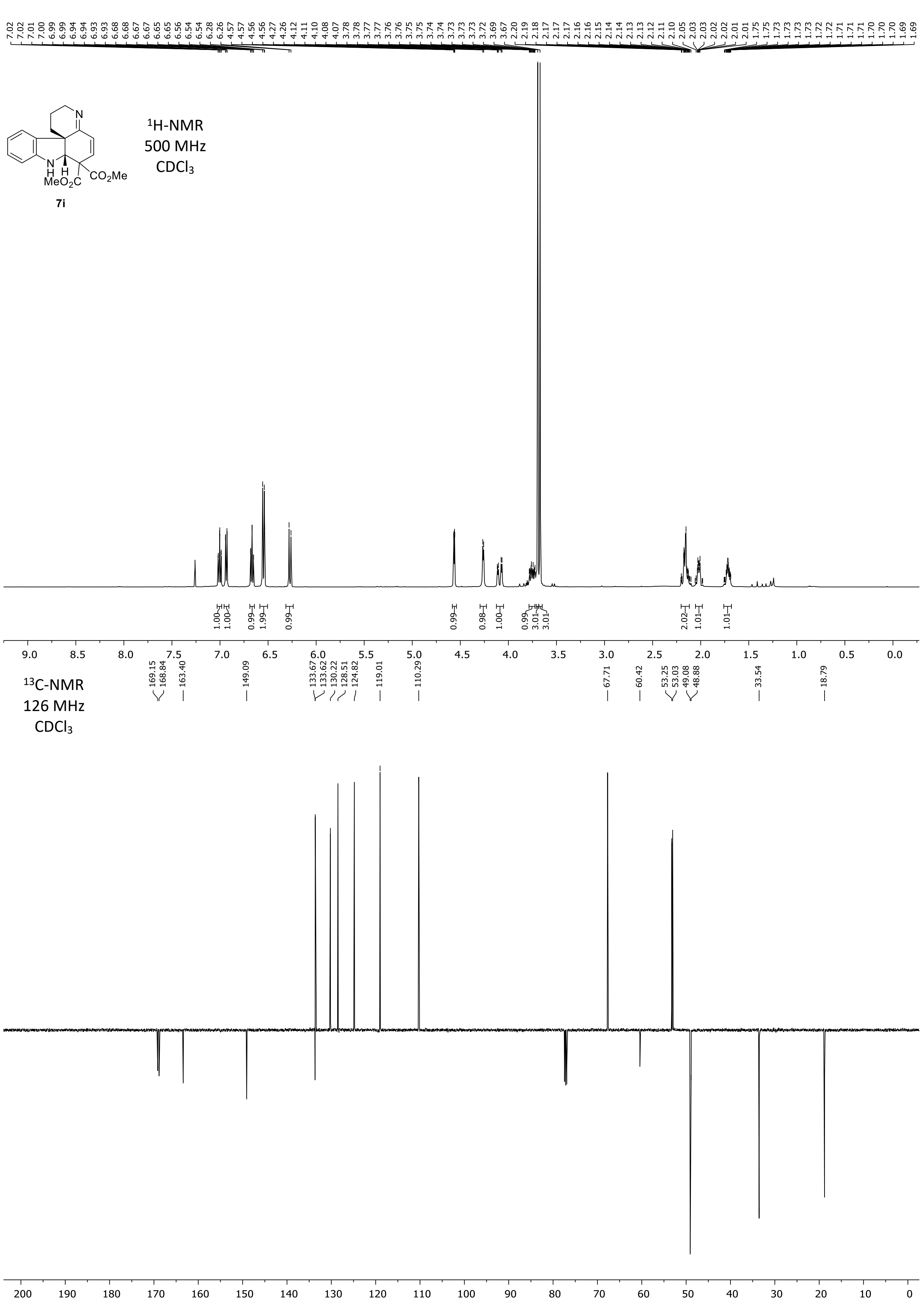
7.02 7.02 7.01 7.00 6.99 6.99 6.94 6.94 6.93 6.93 6.68 6.68 6.67 6.65 6.65 6.56 6.56 6.54 6.54 6.28 6.26 4.57 4.57 4.56 4.56 4.27 4.26 4.12 4.11 4.10 4.08 4.07 3.78 3.78 3.77 3.77 3.76 3.76 3.75 3.75 3.74 3.74 3.73 3.73 3.73 3.72 3.69 3.67 2.20 2.19 2.18 2.17 2.17 2.17 2.16 2.16 2.15 2.14 2.14 2.13 2.13 2.12 2.12 2.11 2.10 2.05 2.03 2.03 2.02 2.02 2.01 2.01 1.75 1.75 1.73 1.73 1.73 1.73 1.72 1.71 1.71 1.71 1.70 1.70 1.69 1.69
N
H H
MeO2C
CO2Me
7i
1H-NMR
500 MHz
CDCl3
1.00 1.00 0.99 1.99 0.99 0.99 0.98 1.00 0.99 3.01 3.01 2.02 1.01 1.01
9.0 8.5 8.0 7.5 7.0 6.5 6.0 5.5 5.0 4.5 4.0 3.5 3.0 2.5 2.0 1.5 1.0 0.5 0.0
13C-NMR
126 MHz
CDCl3
169.15 168.84 163.40 149.09 133.67 133.62 130.22 128.51 124.82 119.01 110.29 67.71 60.42 53.25 53.03 49.08 48.88 33.54 18.79
200 190 180 170 160 150 140 130 120 110 100 90 80 70 60 50 40 30 20 10 0

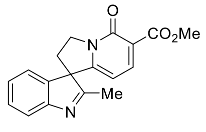

15j

^{1}H-NMR
600 MHz
$CDCl_3$

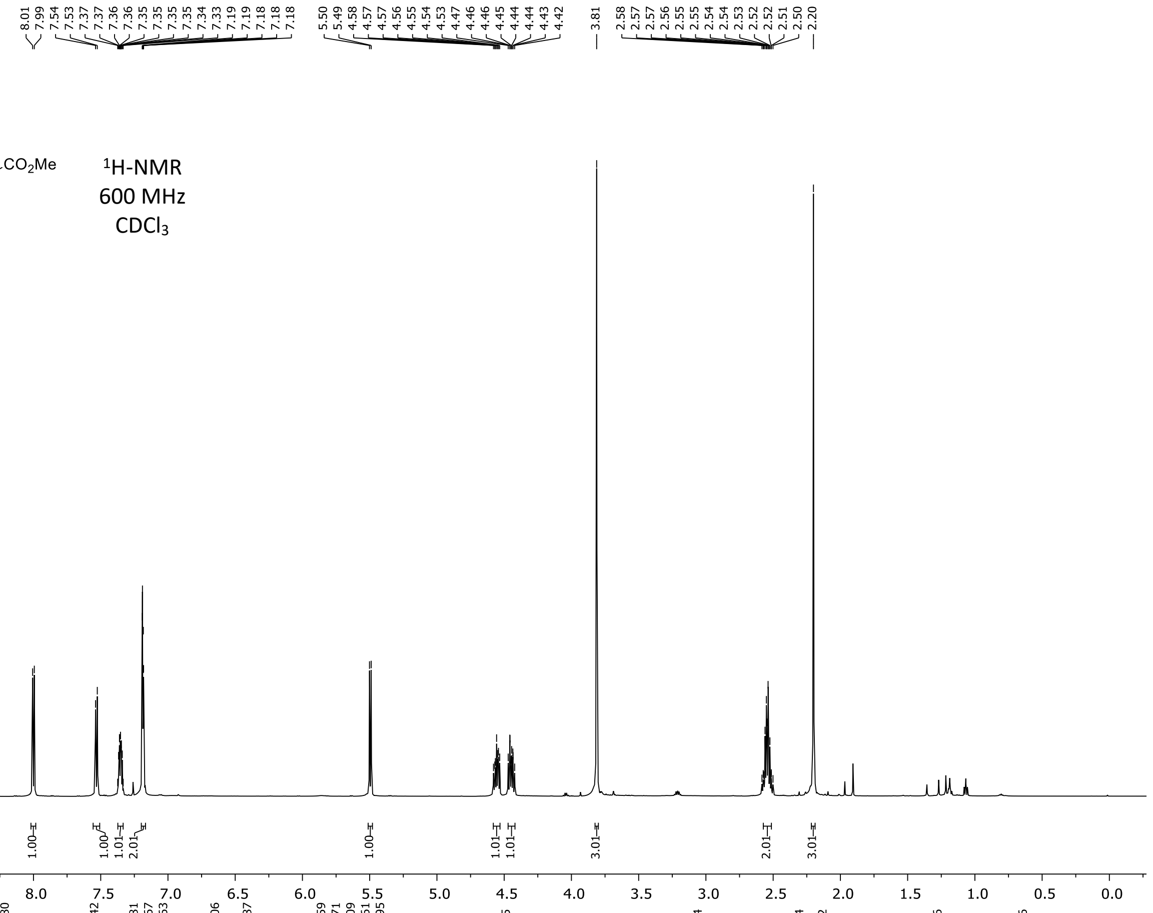

^{13}C-NMR
151 MHz
$CDCl_3$

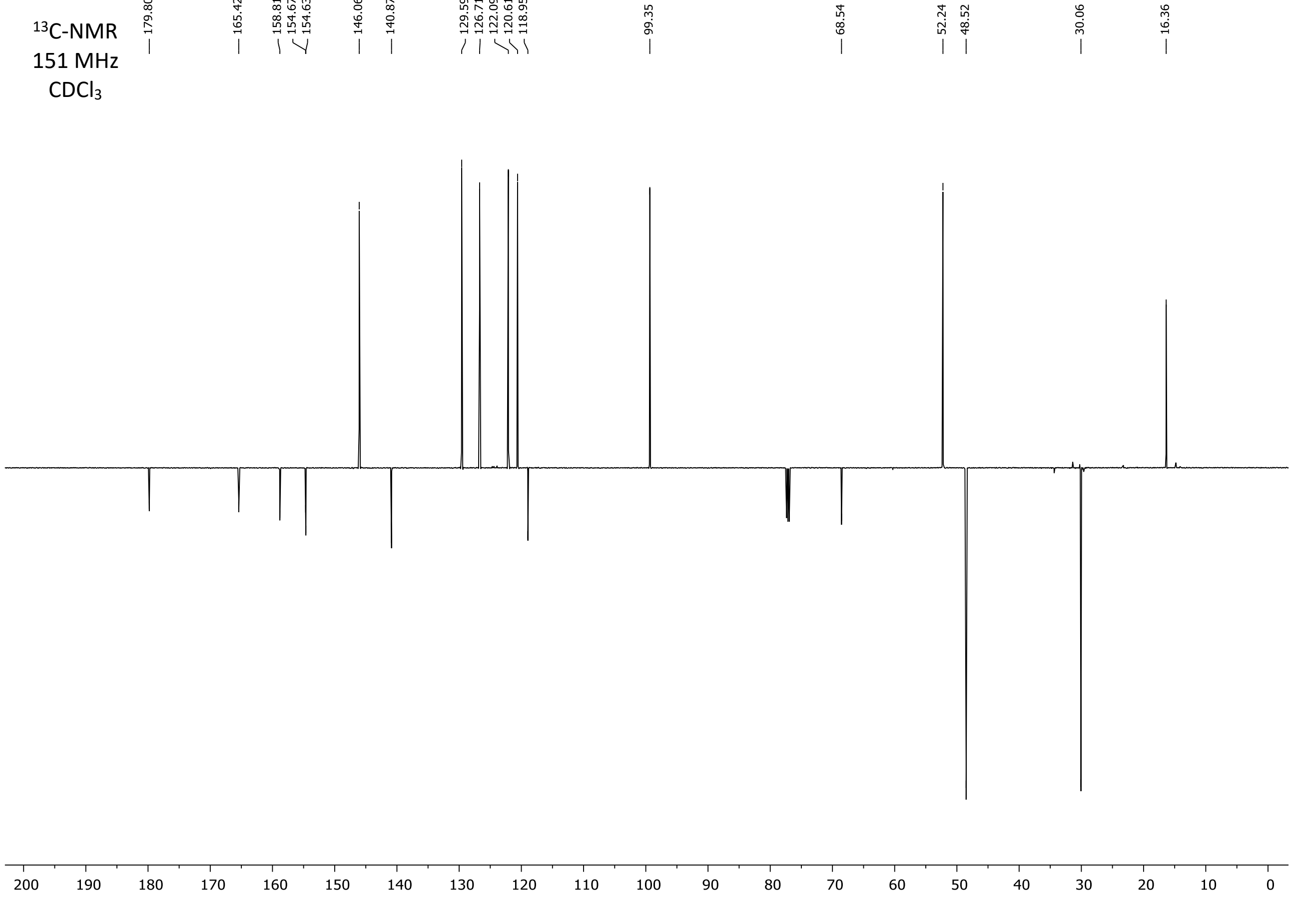

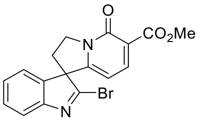

15k

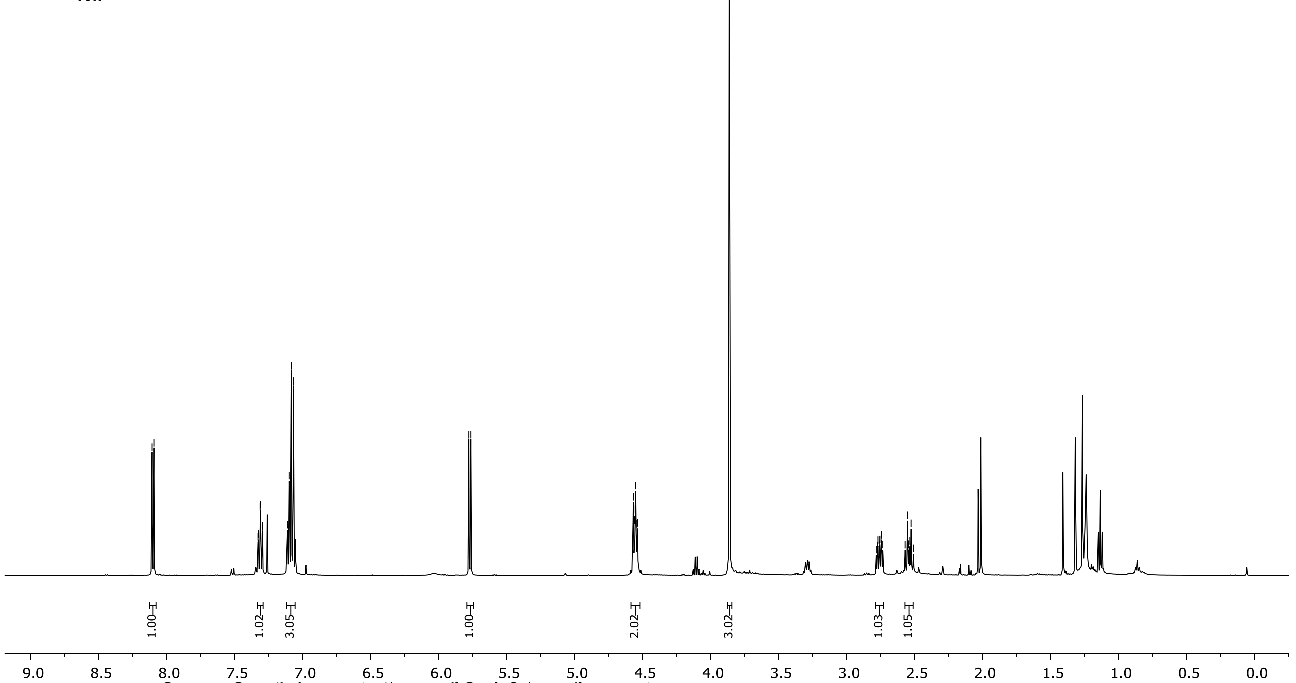

^{1}H-NMR
500 MHz
$CDCl_3$

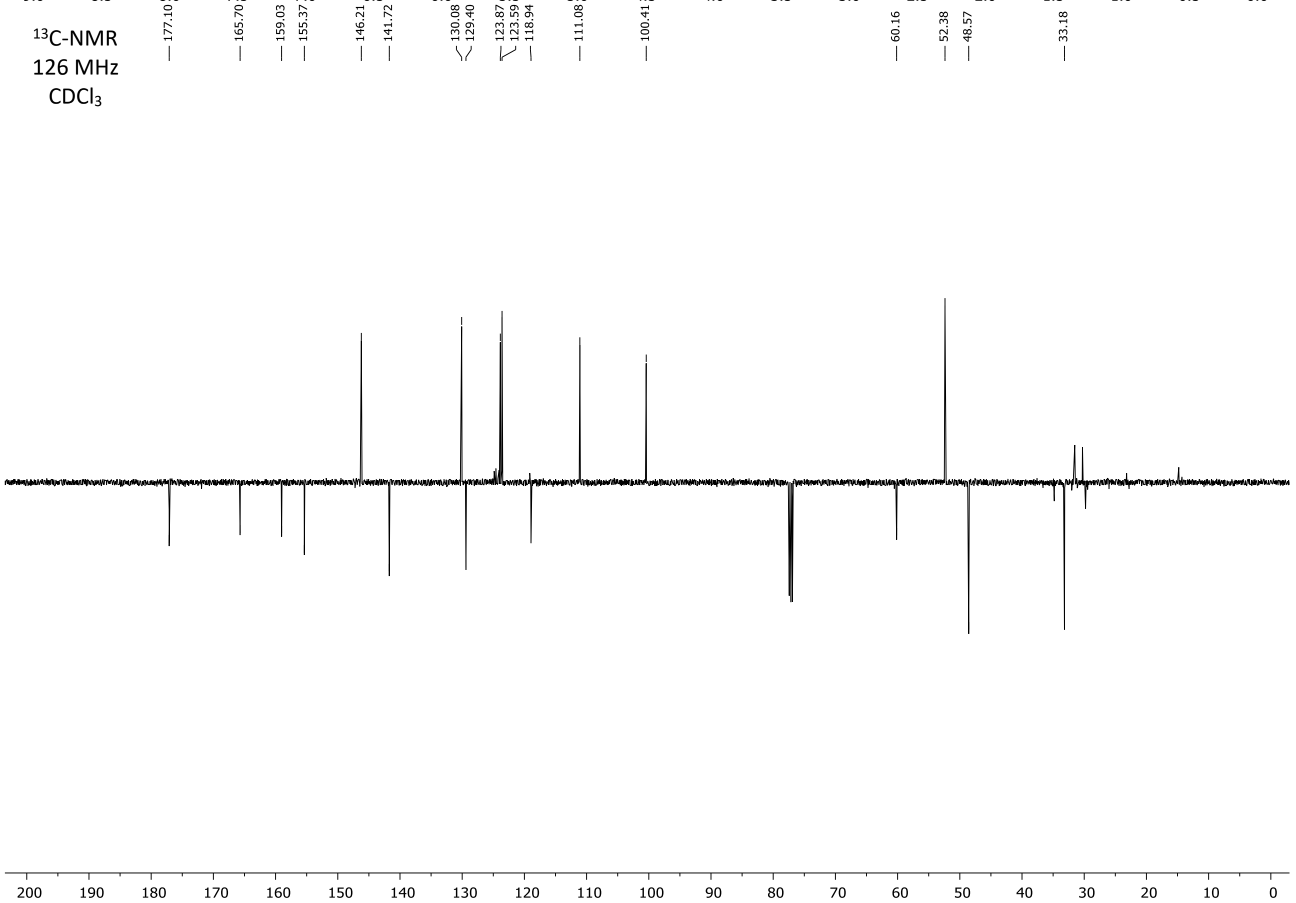

^{13}C-NMR
126 MHz
$CDCl_3$

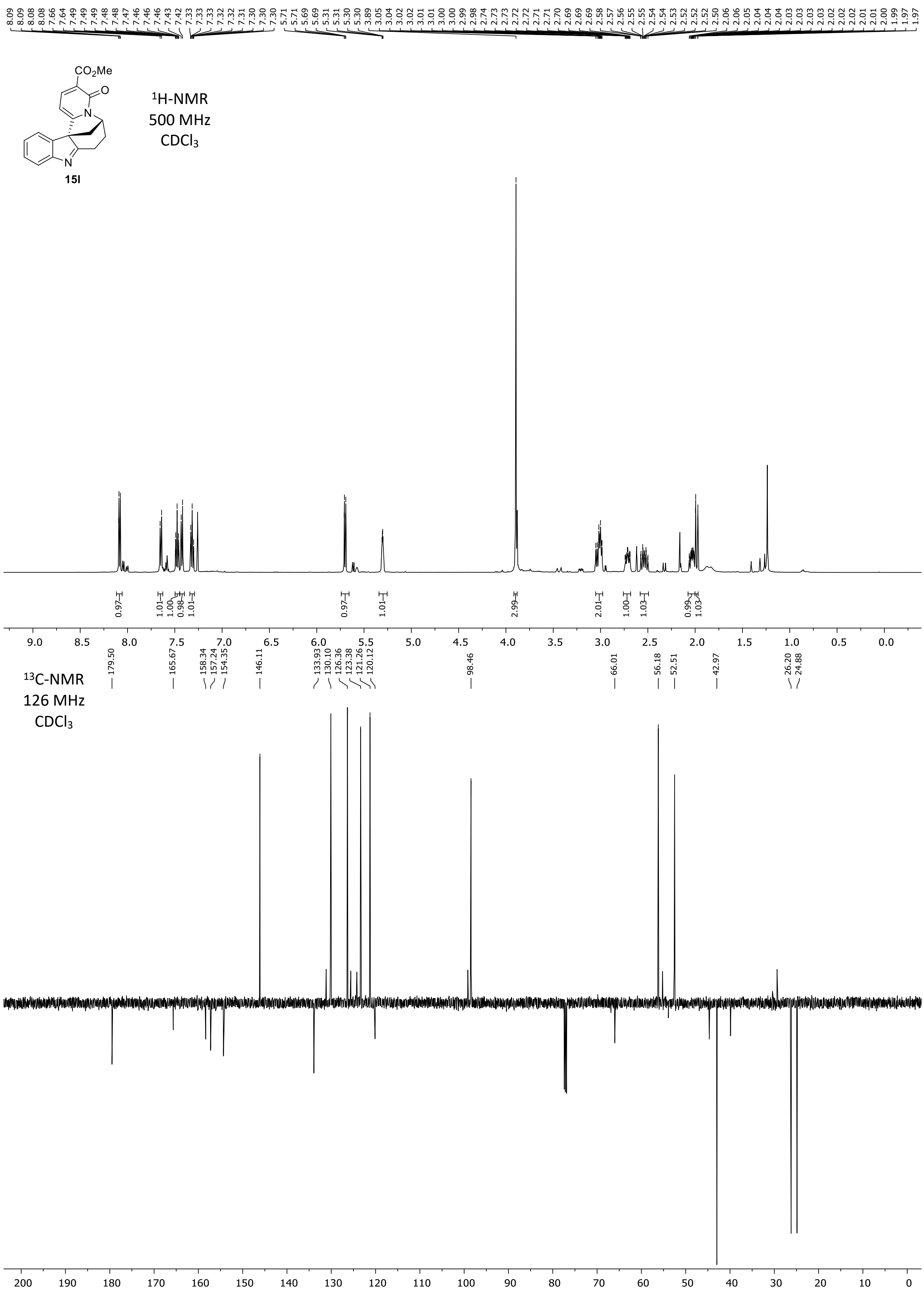

8.09 8.09 8.08 8.08 7.66 7.64 7.49 7.49 7.48 7.47 7.46 7.46 7.43 7.42 7.33 7.33 7.32 7.32 7.31 7.30 7.30 7.30 5.71 5.69 5.31 5.30 5.30 3.89 3.05 3.04 3.02 3.01 3.00 2.99 2.98 2.74 2.73 2.72 2.72 2.71 2.70 2.69 2.69 2.58 2.57 2.56 2.55 2.55 2.54 2.54 2.53 2.52 2.52 2.50 2.06 2.06 2.05 2.04 2.04 2.03 2.03 2.03 2.02 2.02 2.01 2.01 2.00 1.99 1.97 1.97
CO2Me
O
N
N
15l
1H-NMR
500 MHz
CDCl3
0.97 1.01 1.00 0.98 1.01 0.97 1.01 2.99 2.01 1.00 1.03 0.99 1.03
9.0 8.5 8.0 7.5 7.0 6.5 6.0 5.5 5.0 4.5 4.0 3.5 3.0 2.5 2.0 1.5 1.0 0.5 0.0
179.50 165.67 158.34 157.24 154.35 146.11 133.93 130.10 126.36 123.38 121.26 120.12 98.46 66.01 56.18 52.51 42.97 26.20 24.88
13C-NMR
126 MHz
CDCl3
200 190 180 170 160 150 140 130 120 110 100 90 80 70 60 50 40 30 20 10 0

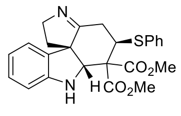

16

^{1}H-NMR
500 MHz
$CDCl_3$

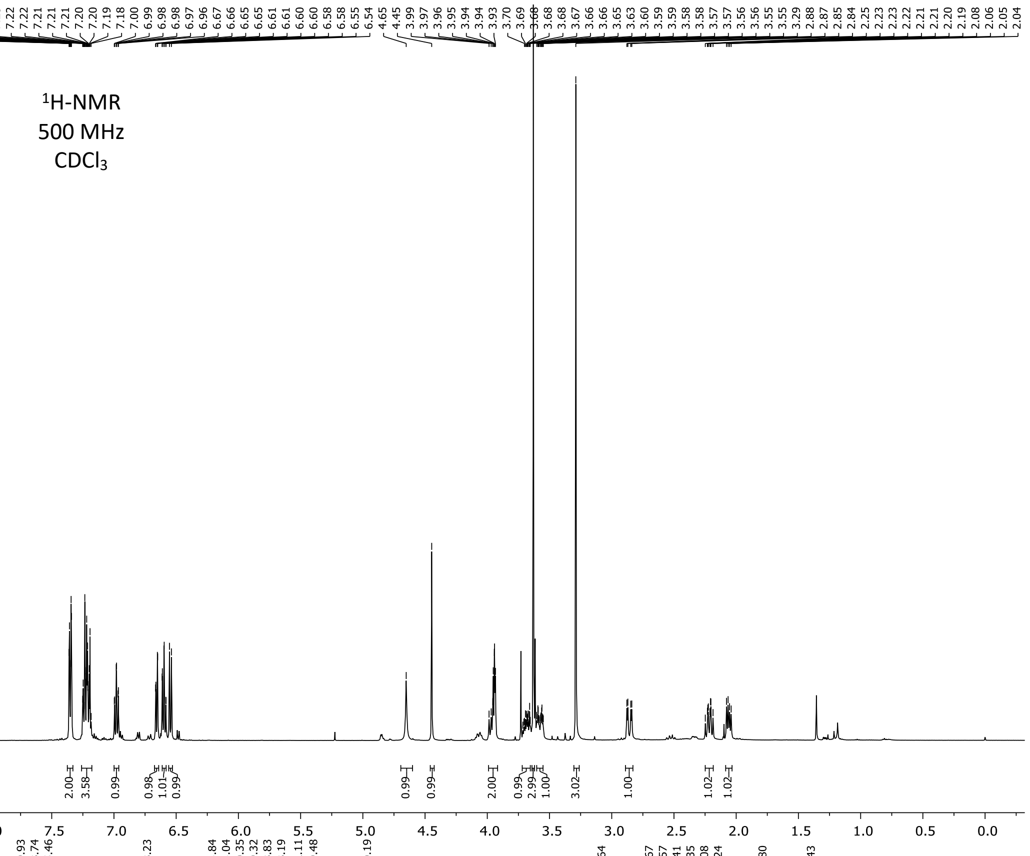

^{13}C-NMR
126 MHz
$CDCl_3$

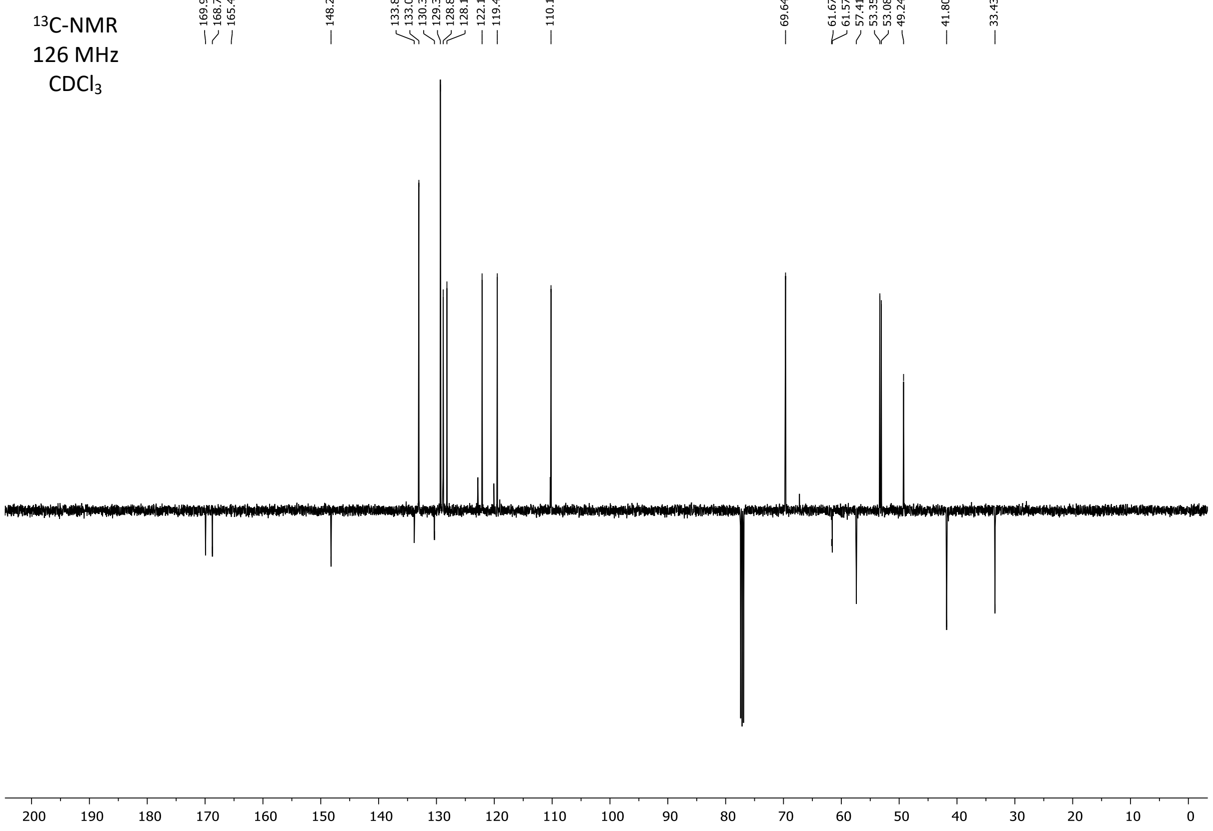

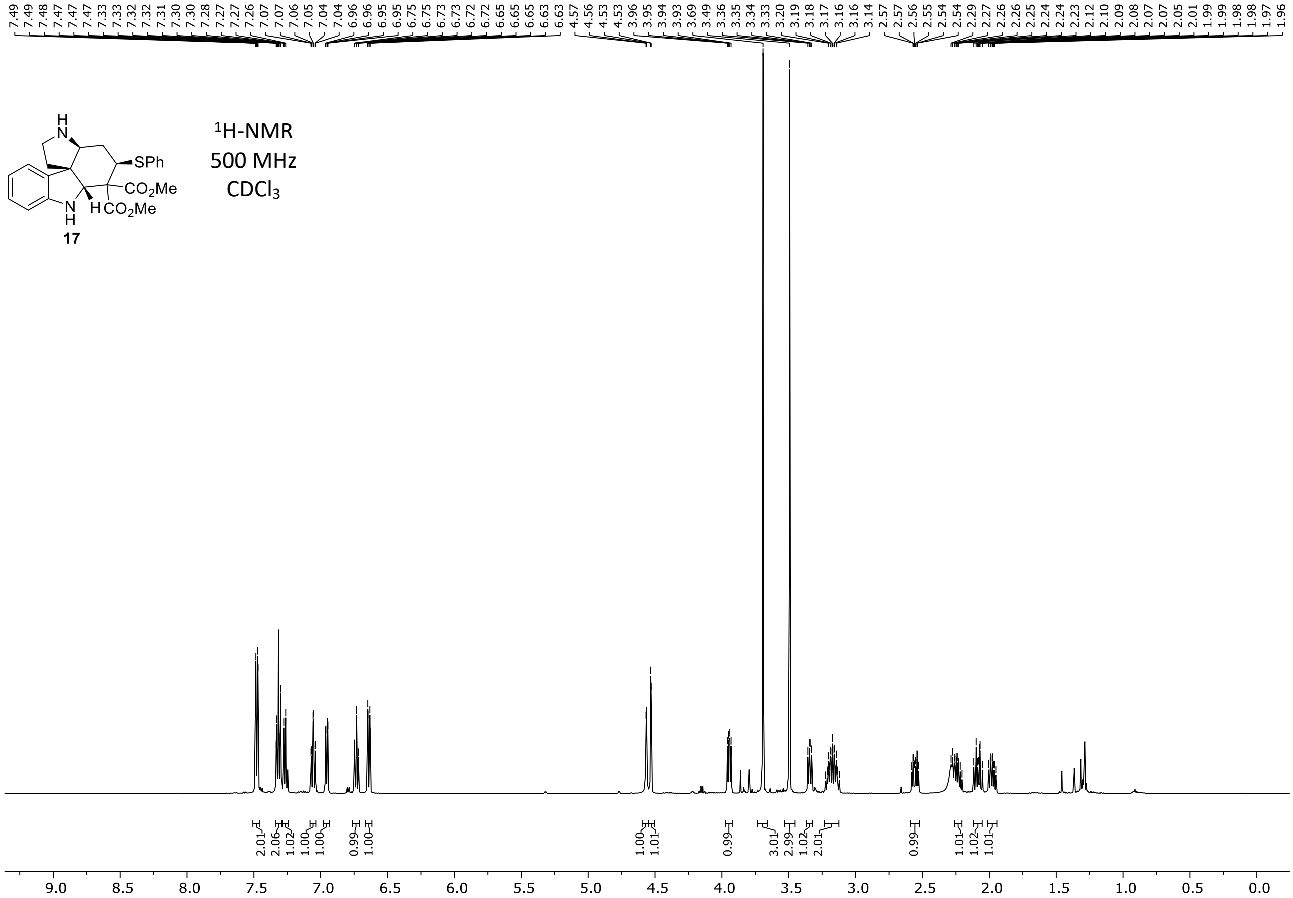
7.49 7.49 7.48 7.47 7.47 7.33 7.33 7.32 7.32 7.31 7.30 7.30 7.28 7.27 7.27 7.26 7.07 7.07 7.06 7.05 7.04 7.04 6.96 6.96 6.95 6.95 6.75 6.75 6.73 6.73 6.72 6.72 6.65 6.65 6.65 6.63 6.63 4.57 4.56 4.53 4.53 3.96 3.95 3.94 3.93 3.69 3.49 3.36 3.35 3.34 3.33 3.20 3.19 3.18 3.17 3.16 3.16 3.14 2.57 2.57 2.56 2.55 2.54 2.54 2.29 2.27 2.26 2.26 2.25 2.24 2.24 2.23 2.12 2.10 2.09 2.08 2.07 2.07 2.05 2.01 1.99 1.99 1.98 1.98 1.97 1.96
SPh
CO2Me
CO2Me
17
1H-NMR
500 MHz
CDCl3
2.01 2.06 1.02 1.00 1.00 0.99 1.00 1.00 1.01 0.99 3.01 2.99 1.02 2.01 0.99 1.01 1.02 1.01
9.0 8.5 8.0 7.5 7.0 6.5 6.0 5.5 5.0 4.5 4.0 3.5 3.0 2.5 2.0 1.5 1.0 0.5 0.0

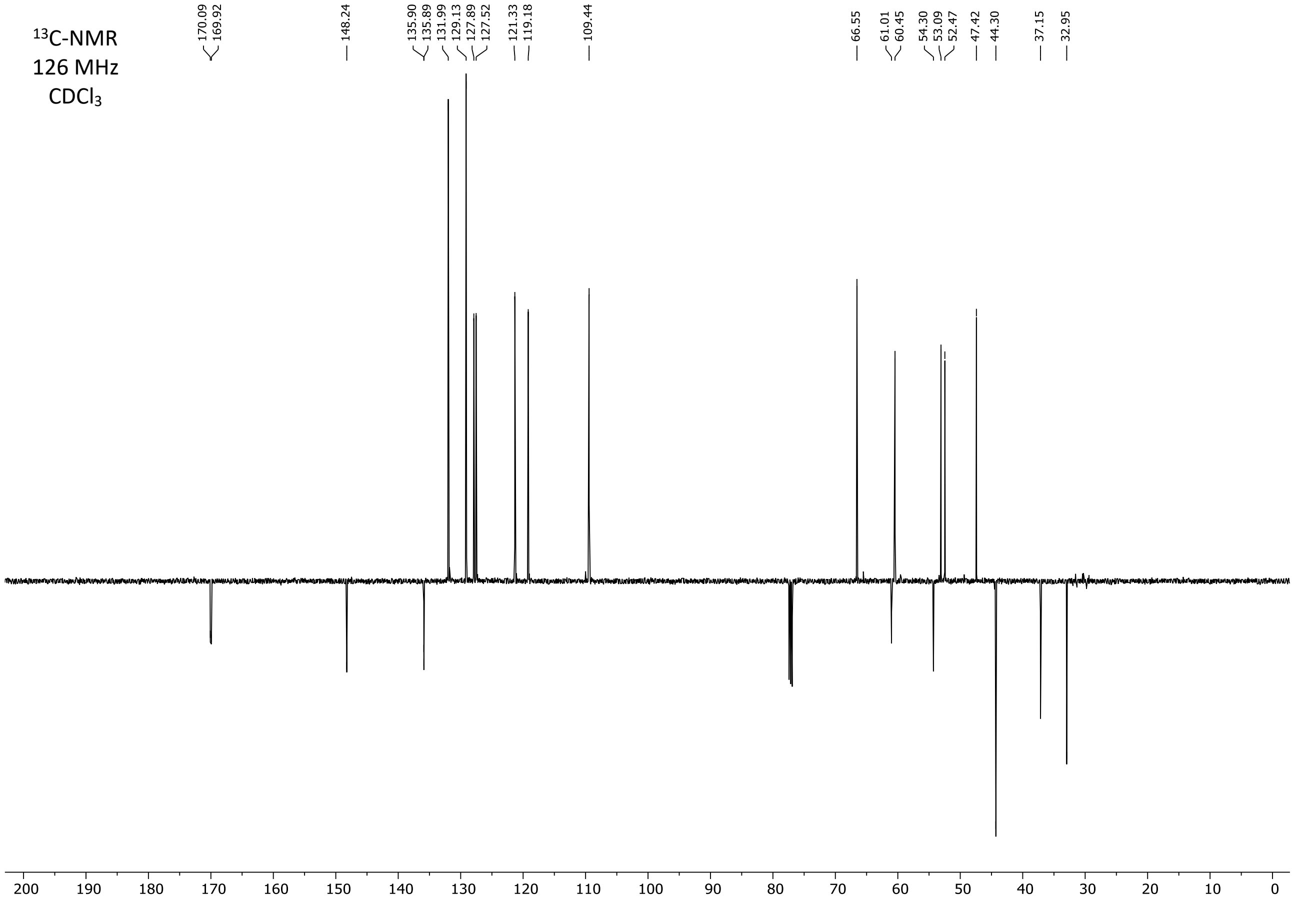
13C-NMR
126 MHz
CDCl3
170.09 169.92 148.24 135.90 135.89 131.99 129.13 127.89 127.52 121.33 119.18 109.44 66.55 61.01 60.45 54.30 53.09 52.47 47.42 44.30 37.15 32.95
200 190 180 170 160 150 140 130 120 110 100 90 80 70 60 50 40 30 20 10 0

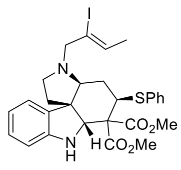

19

^{1}H-NMR
600 MHz
$CDCl_3$

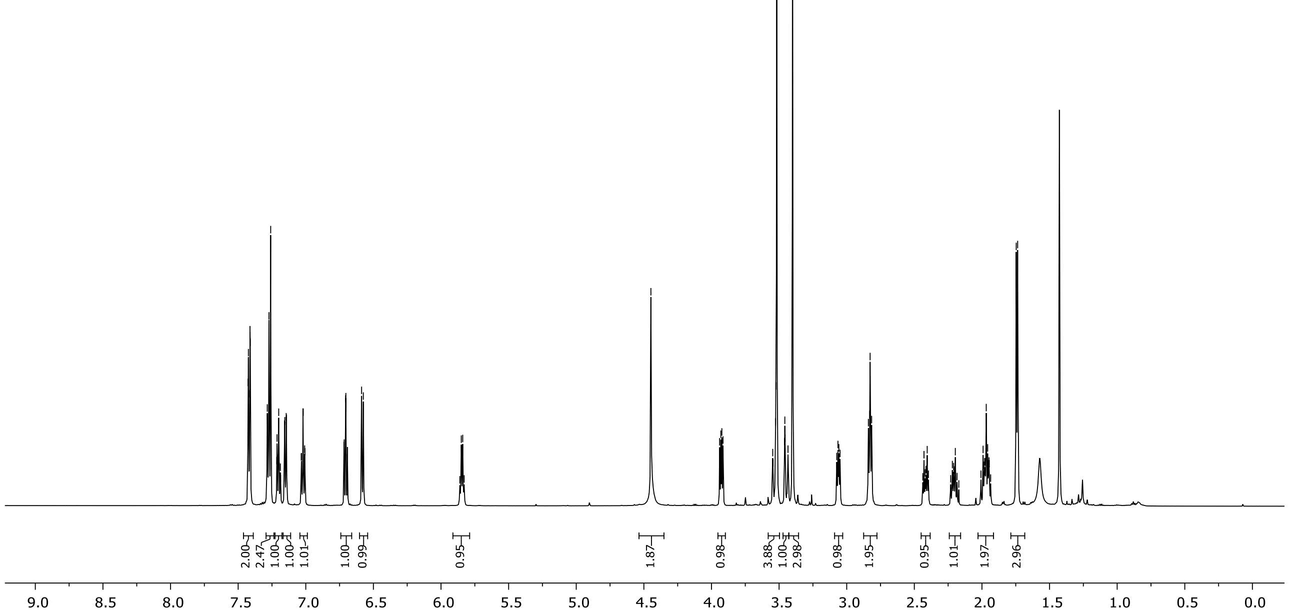

^{13}C-NMR
151 MHz
$CDCl_3$

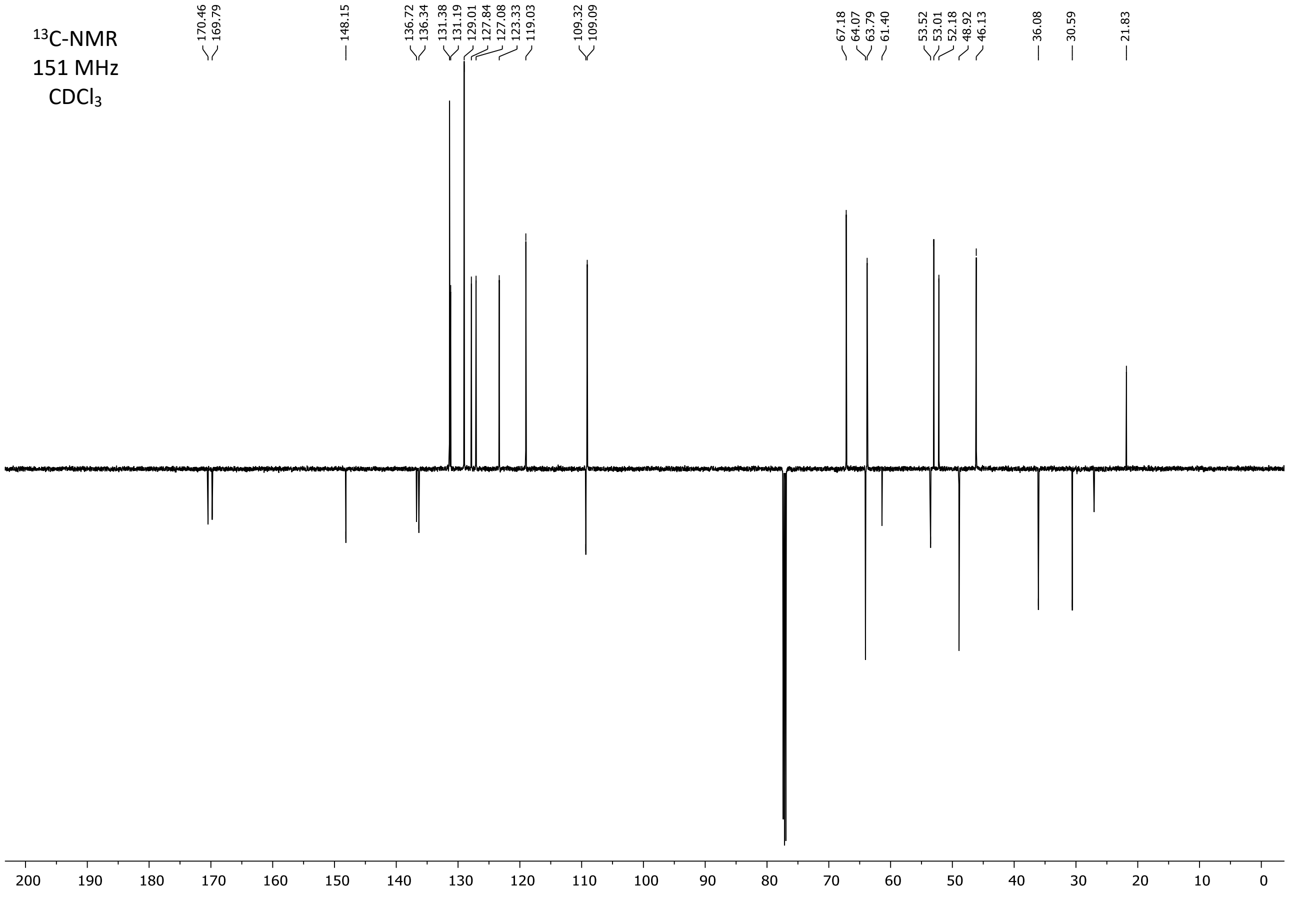

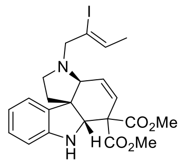

20

^{1}H-NMR
500 MHz
$CDCl_3$

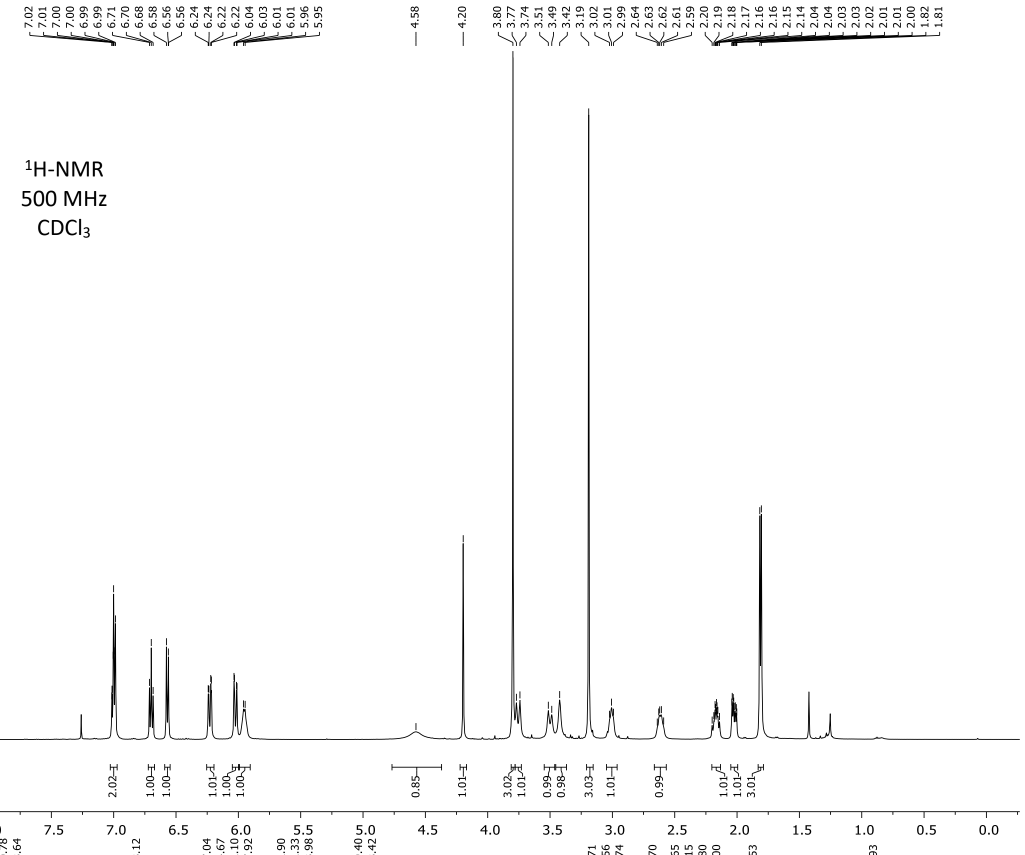

^{13}C-NMR
126 MHz
$CDCl_3$

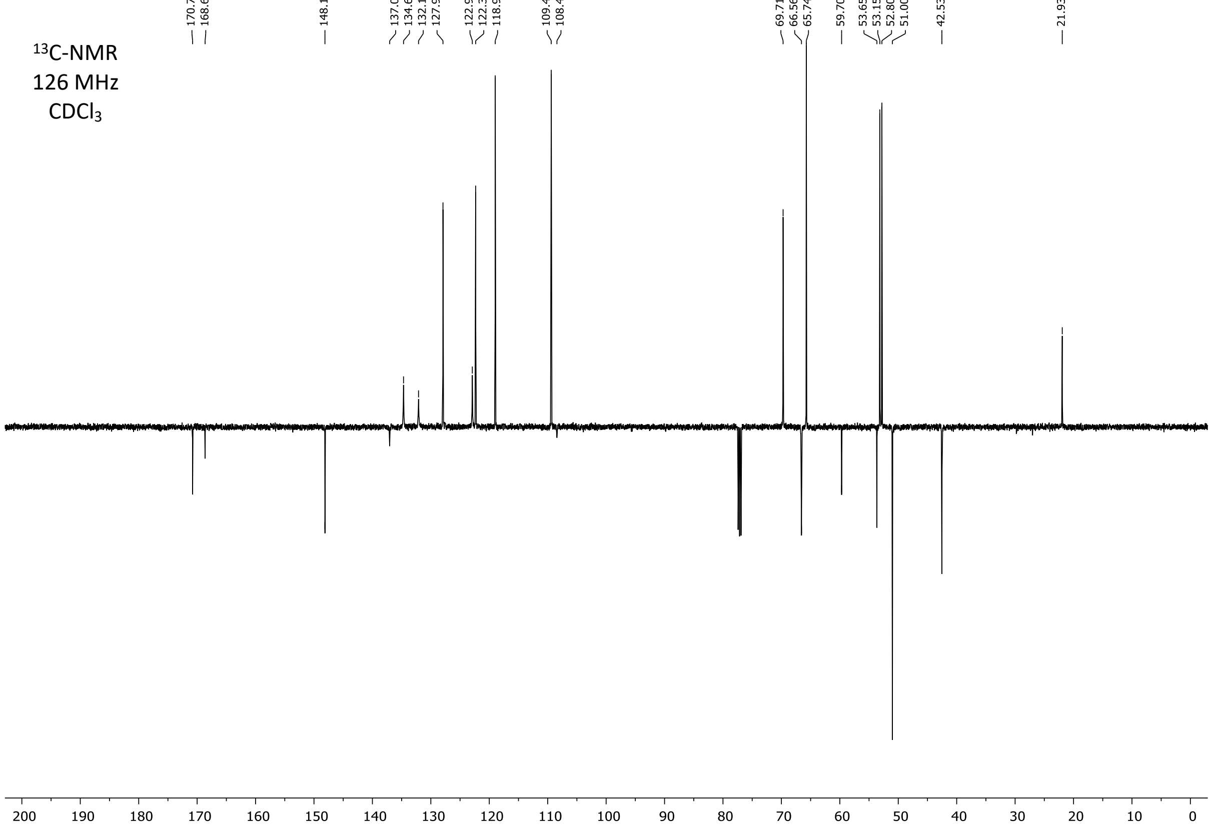

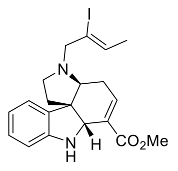

21

^{1}H-NMR
500 MHz
$CDCl_3$

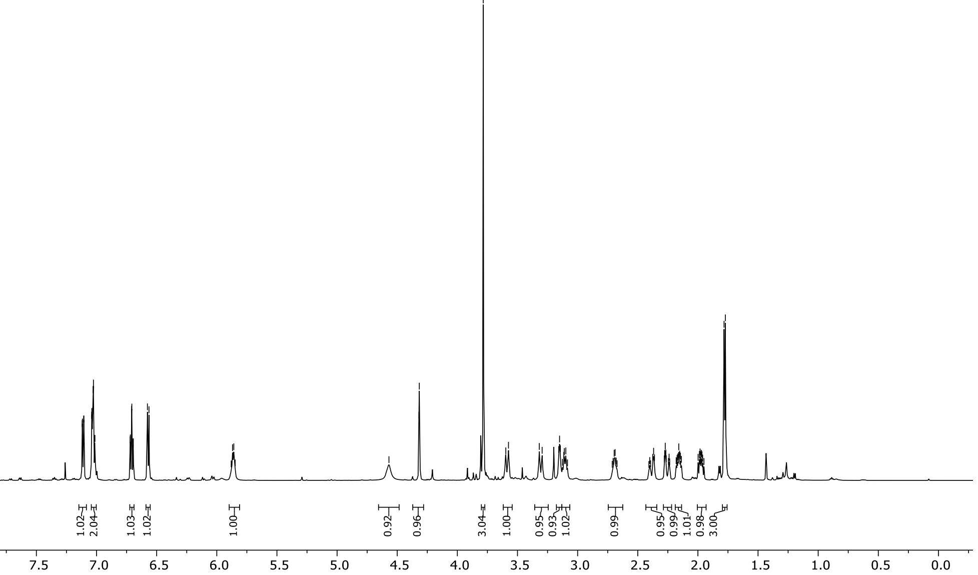

^{13}C-NMR
126 MHz
$CDCl_3$

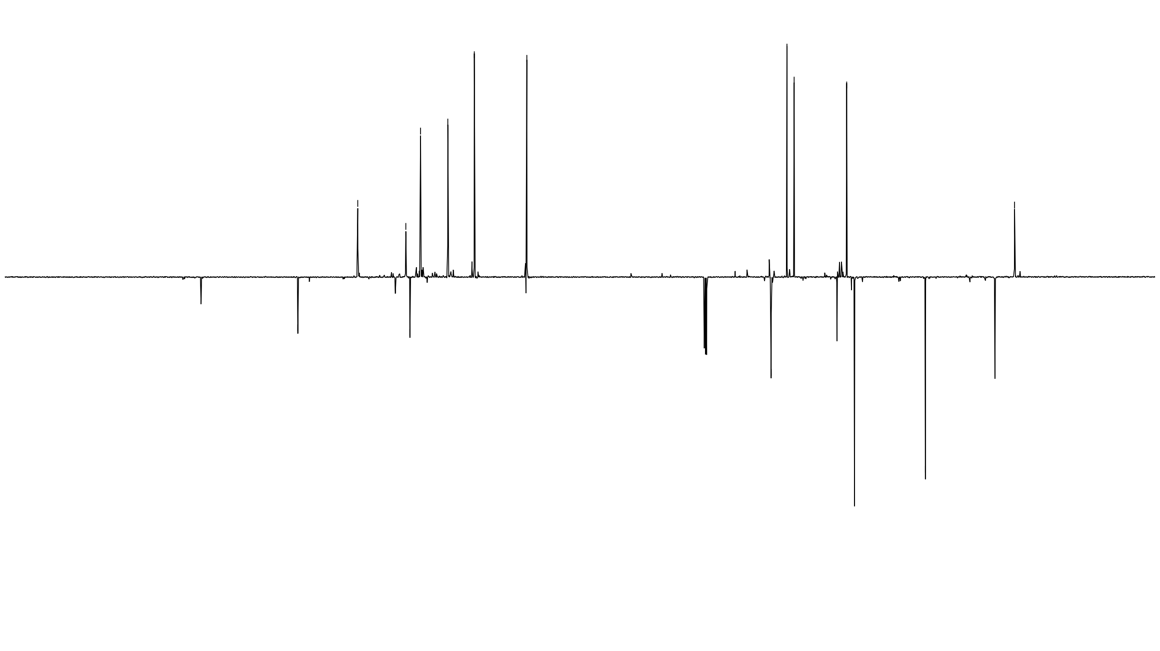

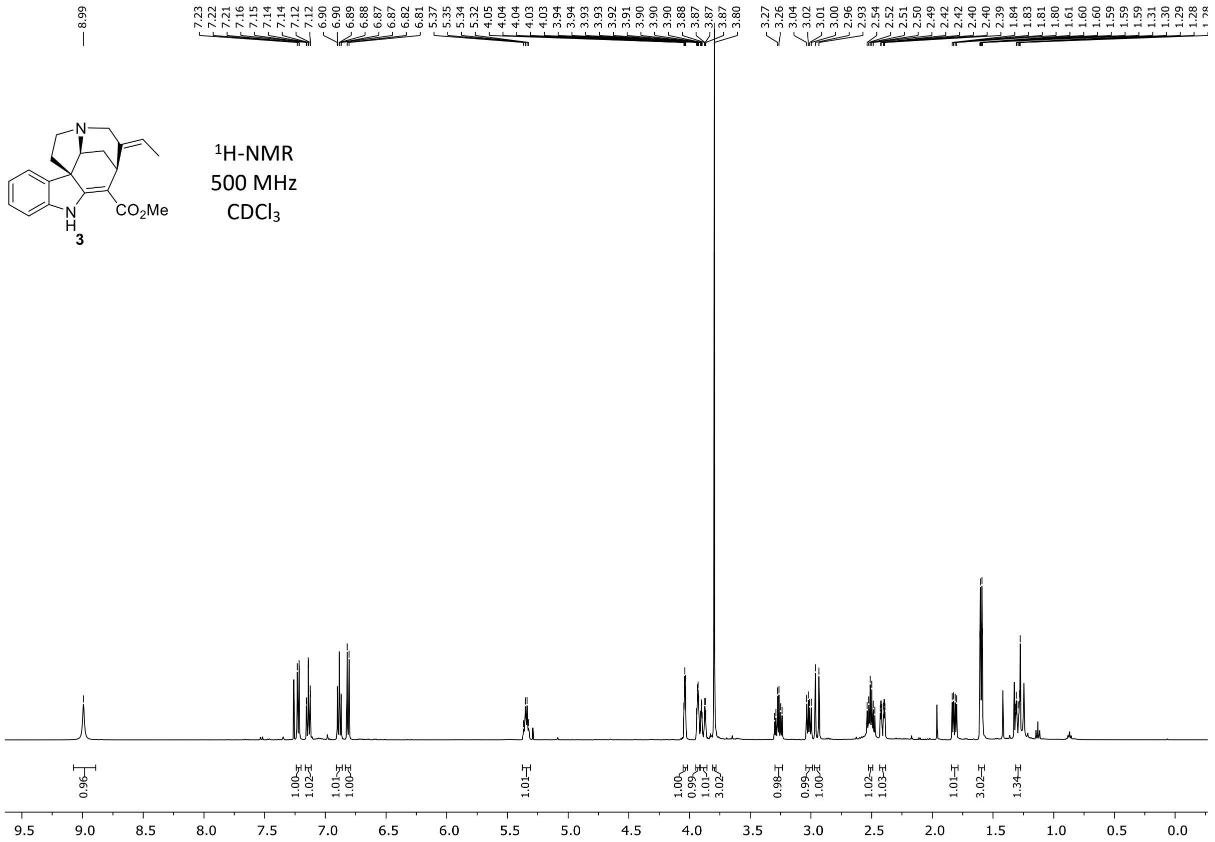

Reference spectrum:[3]

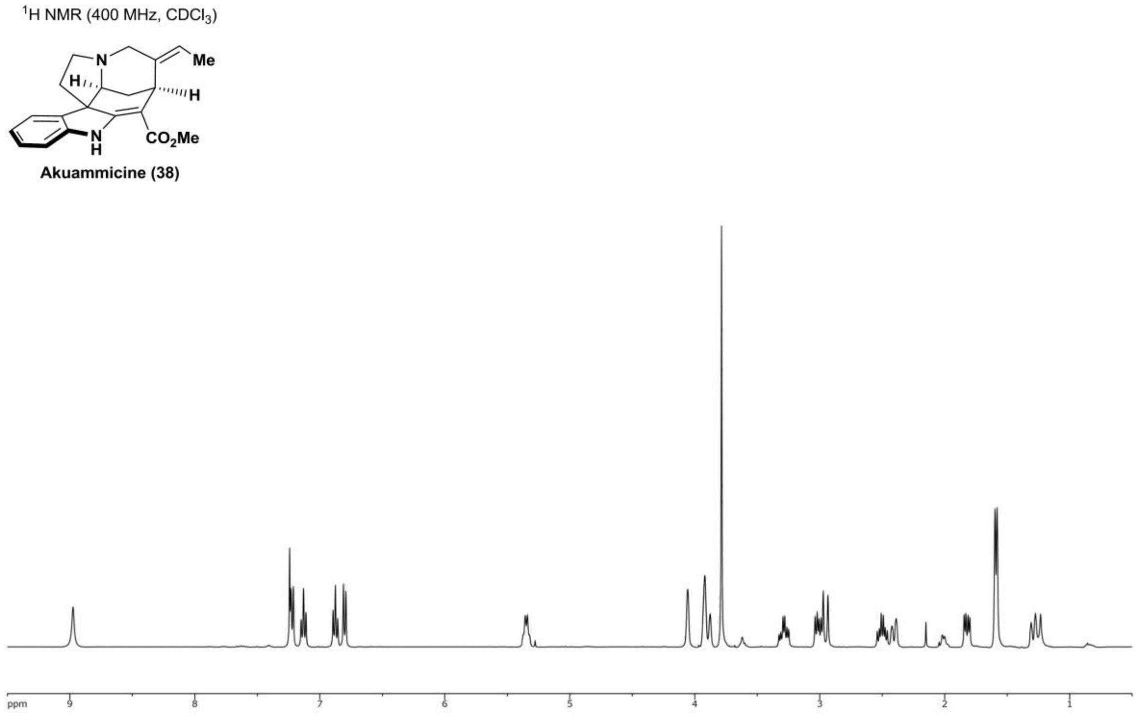

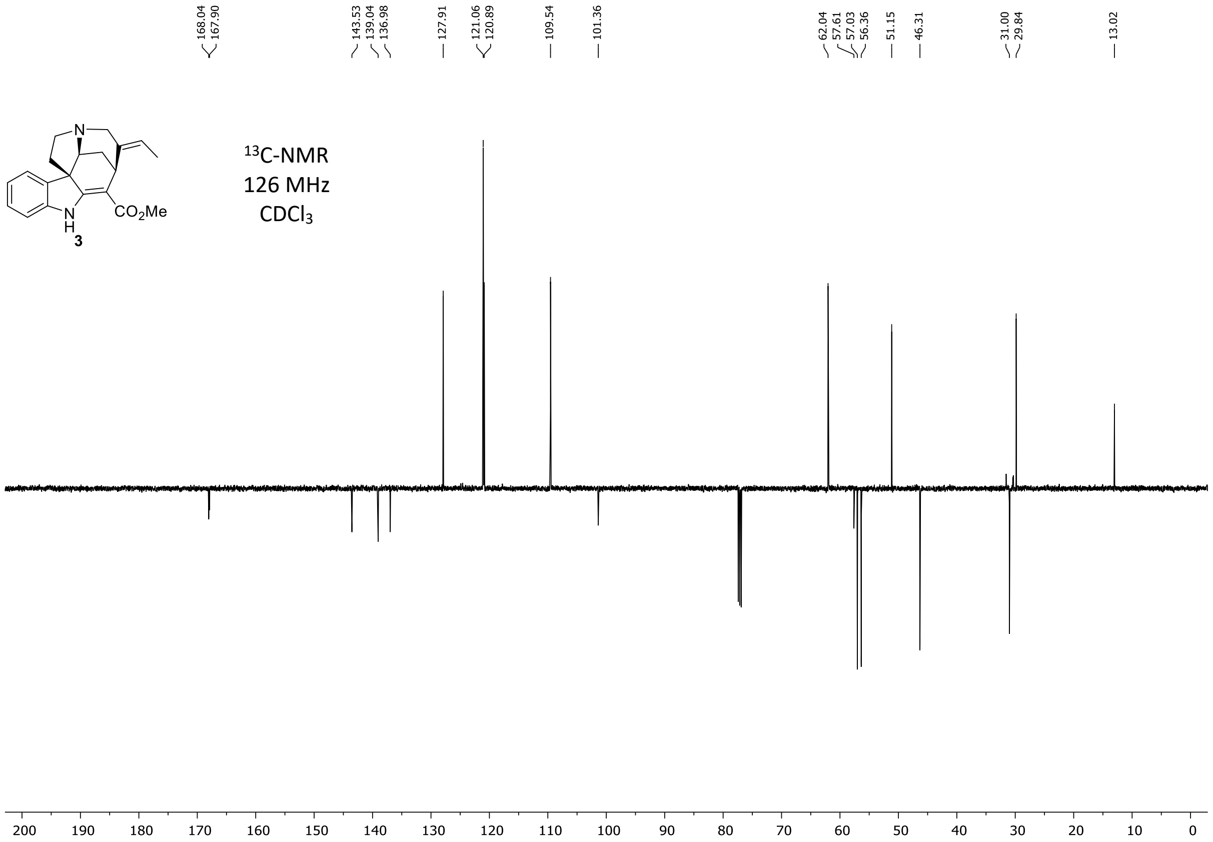

Reference spectrum:[3]

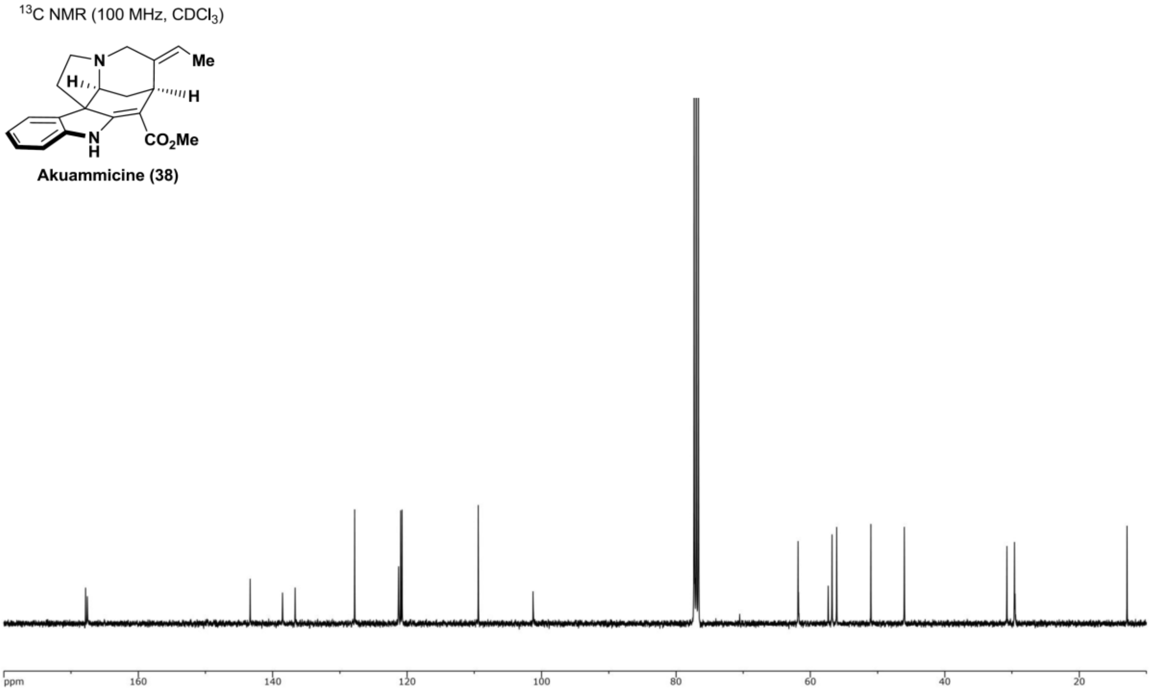

References

[1] P. Liu, J. Wang, J. Zhang, F. G. Qiu, *Org. Lett.*, 2011, **13**, 6426-6428.
[2] L. S. Hutchings-Goetz, C. Yang, J. W. B. Fyfe, T. N. Snaddon*, Angew. Chem. Int. Ed*., 2020, **59**, 17556-17564.
[3] G. S. Lee, G. Namkoong, J. Park and D. Y.-K. Chen, *Chem. Eur. J.*, 2017, **23**, 16189-16193.
